# Supplementary material for: Genetic architecture of brain age and its causal relations with brain and mental disorders
Source: Mol Psychiatry. 2023 May 10;28(7):3111–20. doi: 10.1038/s41380-023-02087-y (PMC10615751; doi:10.1038/s41380-023-02087-y)
Supplement: Supplementary file 1 — Supplementary Information [file 41380_2023_2087_MOESM1_ESM.pdf]

Supporting Information

Genetic architecture of brain age and its casual relations with brain disorders

Leonardsen et al.

Table of Contents

Figure S1. Genetic association to brain age gap in each fold. **4**

Figure S2. QQ plot for the genetic association to brain age gap. **5**

Figure S3. Genic annotations for associated SNPs. **6**

Figure S4. Annotations for associated SNPs. **7**

Figure S5. Gene based association results. **8**

Figure S6. eQTL annotations for rs73185796. **9**

Figure S7. eQTL annotations for rs13132853. **10**

Figure S8. eQTL annotations for rs2790102. **11**

Figure S9. eQTL and sQTL annotations for rs7461069. **12**

Figure S10. eQTL and sQTL annotations for rs4880424. **13**

Figure S11. eQTL and sQTL annotations for rs17203398. **14**

Figure S12. eQTL annotations for rs2106786. **15**

Figure S13. Conditional QQ plot for MDD vs BAG. **16**

Figure S14. Conditional QQ plot for SCZ vs BAG. **17**

Figure S15. Conditional QQ plot for PD vs BAG excluding chr17. **18**

Table S1. Non-UK Biobank model training datasets. **19**

Table S2. Characteristics for the five folds data from UK Biobank. **20**

Table S3. Statistics for lead SNPs of each associated region in each fold. **21**

Table S4. Statistics for lead SNPs of each associated region in each replication samples. **22**

Table S5 Function annotation for genes in Figure 1c. **23**

Table S6. Harmonized instrumental SNPs ($p<5x10^{-8}$) for the BAG to SCZ MR. **31**

Table S7. Harmonized instrumental SNPs ($p<5x10^{-8}$) for the BAG to BIP MR. **32**

Table S8. Harmonized instrumental SNPs ($p<5x10^{-8}$) for the BAG to MDD MR. **33**

Table S9. Harmonized instrumental SNPs ($p<5x10^{-8}$) for the BAG to AD MR. **34**

Table S10. Harmonized instrumental SNPs ($p<5x10^{-8}$) for the BAG to PD MR. **35**

Table S11. Harmonized instrumental SNPs ($p<5x10^{-8}$) for the SCZ to BAG MR. **36**

Table S12. Harmonized instrumental SNPs ($p<5x10^{-8}$) for the BIP to BAG MR. **45**

Table S13. Harmonized instrumental SNPs ($p<5x10^{-8}$) for the MDD to BAG MR. **48**

Table S14. Harmonized instrumental SNPs (p<5x10^{-8}) for the AD to BAG MR. ***49***
Table S15. Harmonized instrumental SNPs (p<5x10^{-8}) for the PD to BAG MR....................... ***51***
Table S16. Statistical finemapping for associated loci. ***52***
Table S17. eQTL statistics for rs73185796 from GTEx v8 portal. ***53***
Table S18. eQTL statistics for rs13132853 from GTEx v8 portal. ***54***
Table S19. eQTL statistics for rs2790102 from GTEx v8 portal. ***55***
Table S20. eQTL statistics for rs17203398 from GTEx v8 portal. ***56***
Table S21. eQTL statistics for rs2106786 from GTEx v8 portal. ***57***
Table S22. sQTL statistics for rs2106786 from GTEx v8 portal. ***75***
Table S23. MR results using BAG as exposure... ***82***
Table S24. MR results using disorders as exposure. ***83***
References ***84***

Replication analysis

Genetic data

We attempted to replicate the eight associations to BAG using three independent datasets, the Alzheimer's Disease Neuroimaging Initiative phase 1 (ADNI I) and 2 (ADNI II) and a healthy control dataset (Local sample) that have been previously published in Ripke et al[1] . Out local sample has been imputed by psychiatry genomic consortium using their standardized pipeline Ricopili[2]. However, for the two ADNI datasets, only genotyped data are publicly available. We imputed these genotypes to the 1000 Genomes Projects reference data, using Genipe[3]. The default parameters of Genipe were used. After imputation, we excluded SNPs having MAF < 0.01, HWE p <10-6, and imputation R-sq <0.3 from subsequent analysis.

Image data

MRI data for ANDI sample were obtained from http://adni.loni.usc.edu/ with permission. Brain scans for our local sample were performed at Oslo University Hospital and published in previous works[4-6]. The same protocols of image preprocessing and BAG estimation used in discovery sample were applied to these replication sample.

Replication association studies

We performed association test for each of the eight SNPs in each of the three replication datasets separately. For each analysis, chronological age, sex and the top ten principal components were included as covariates. Association results are shown in Supplementary Table S4.

Supplementary Acknowledgement

We thank the ADNI consortium for making their data publicly accessible. The ADNI was launched in 2003 as a public-private partnership, led by Principal Investigator Michael W. Weiner, MD. ADNI consists of 4 waves, the later is still ongoing (ADNI 3). A complete listing of ADNI investigators can be found at http://adni.loni.usc.edu/wp-content/uploads/how_to_apply/ADNI_Acknowledgement_List.pdf. Data collection and sharing for this project was funded by the ADNI (NIH Grant U01 AG024904) and DOD ADNI (Department of Defense award number W81XWH-12-2-0012). ADNI is funded by the National Institute on Aging, the National Institute of Biomedical Imaging and Bioengineering, and through generous contributions from the following: AbbVie, Alzheimer's Association; Alzheimer's Drug Discovery Foundation; Araclon Biotech; BioClinica, Inc.; Biogen; Bristol-Myers Squibb Company; CereSpir, Inc.; Cogstate Eisai Inc.; Elan Pharmaceuticals, Inc.; Eli Lilly and Company; EuroImmun; F. Hoffmann-La Roche Ltd and its affiliated company Genentech, Inc.; Fujirebio; GE Healthcare; IXICO Ltd.; Janssen Alzheimer Immunotherapy Research & Development, LLC.; Johnson & Johnson Pharmaceutical Research & Development LLC.; Lumosity; Lundbeck; Merck & Co., Inc.; Meso Scale Diagnostics, LLC.; NeuroRx Research; Neurotrack Technologies; Novartis Pharmaceuticals Corporation; Pfizer Inc.; Piramal Imaging; Servier; Takeda Pharmaceutical Company; and Transition Therapeutics. The Canadian Institutes of Health Research is providing funds to support ADNI clinical sites in Canada and the Saguenay Yout Study. Private sector contributions are facilitated by the Foundation for the National Institutes of Health (http://www.fnih.org). The grantee organization is the Northern California Institute for Research and Education, and the study is coordinated by the Alzheimer's Therapeutic Research Institute at the University of Southern California. ADNI data are disseminated by the Laboratory for Neuro Imaging at the University of Southern California.

Figure S1. Genetic association to brain age gap in each fold.

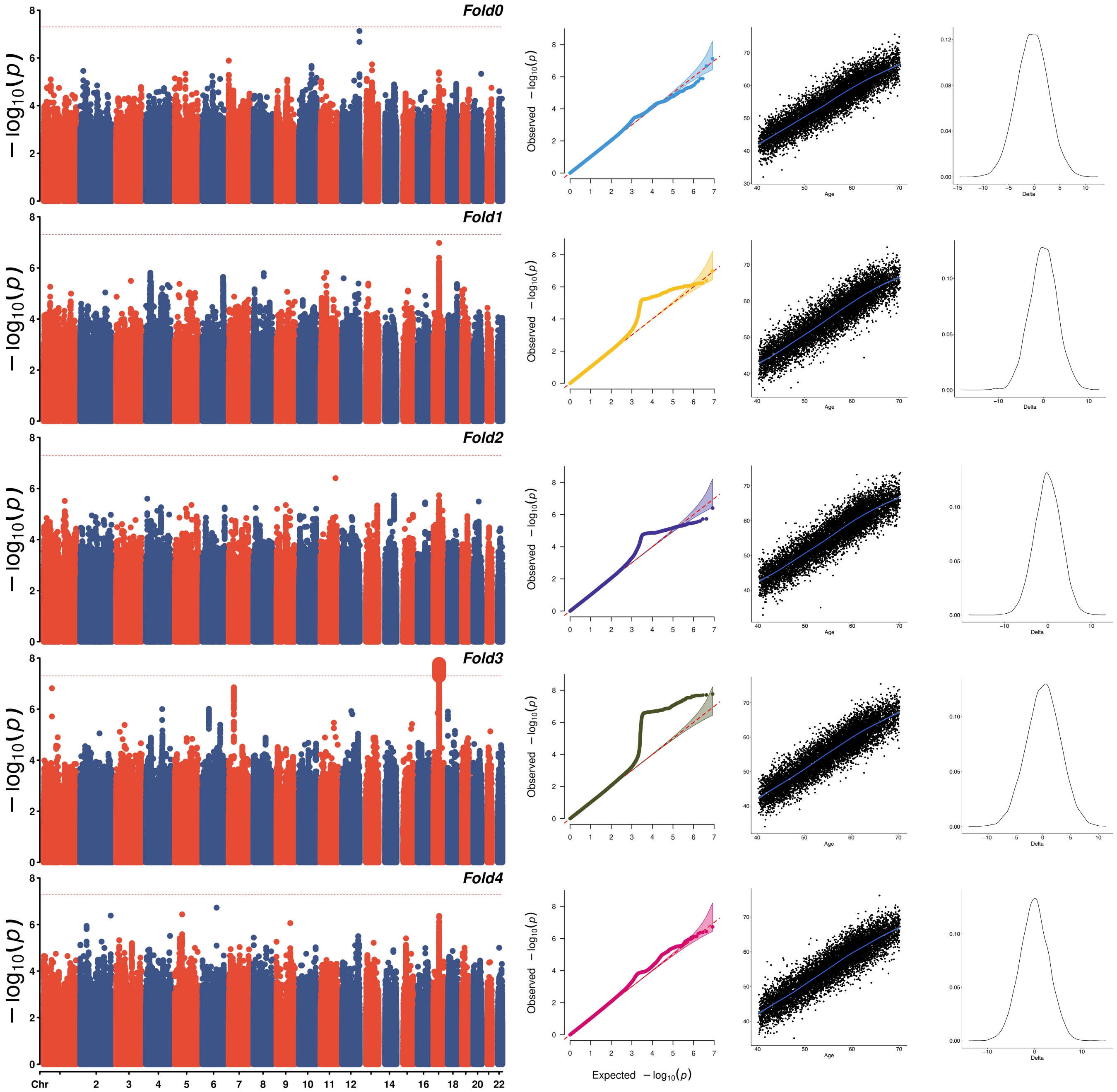

From left to right, GWAS association Manhattan plot and QQ plot, the scatter plot for estimated brain age (y axis) vs. chronological age (x axis), distribution for brain age gap (delta) for each fold of the UKB data (each row). In Manhattan plots, chromosome numbers are on x axes and -log10 association p value on the y axes. In QQ plots, the expected and observed p value on the -log10 scale are shown on the x and y axes. Blue lines in the scatter plots indicated a fitted loess model for each fold.

Figure S2. QQ plot for the genetic association to brain age gap.

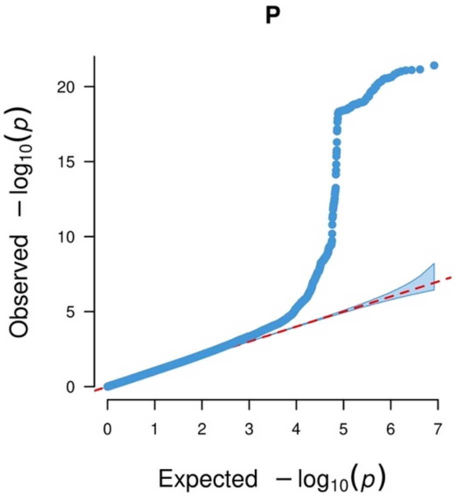

QQ plot for the results of meta-analysis over the GWAS for BAG in each of the five folds of UKB data.

Figure S3. Genic annotations for associated SNPs.

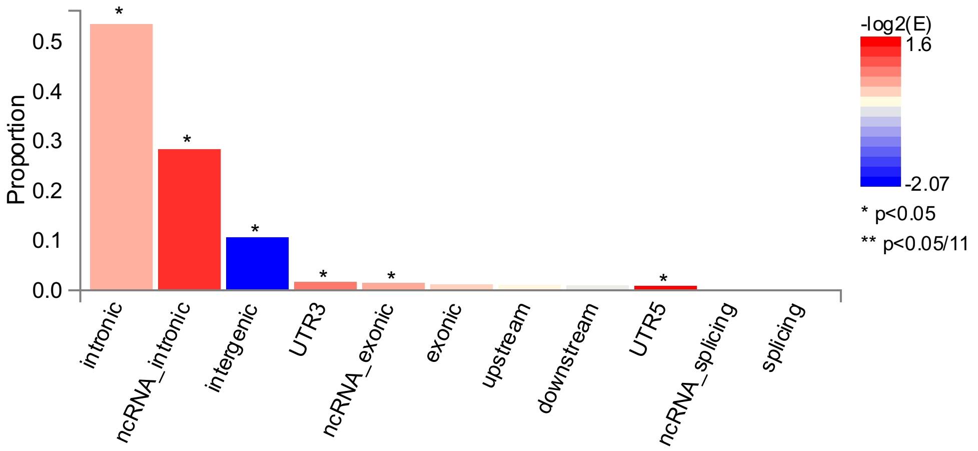

Annotations of associated SNPs to genic elements by FUMA[7]. UTR3, 3 prime untranslated region; ncRNA, non-coding RNA; UTR5, 5 prime untranslated region. Enrichment p values are based on hypergeometric test.

Figure S4. Annotations for associated SNPs.

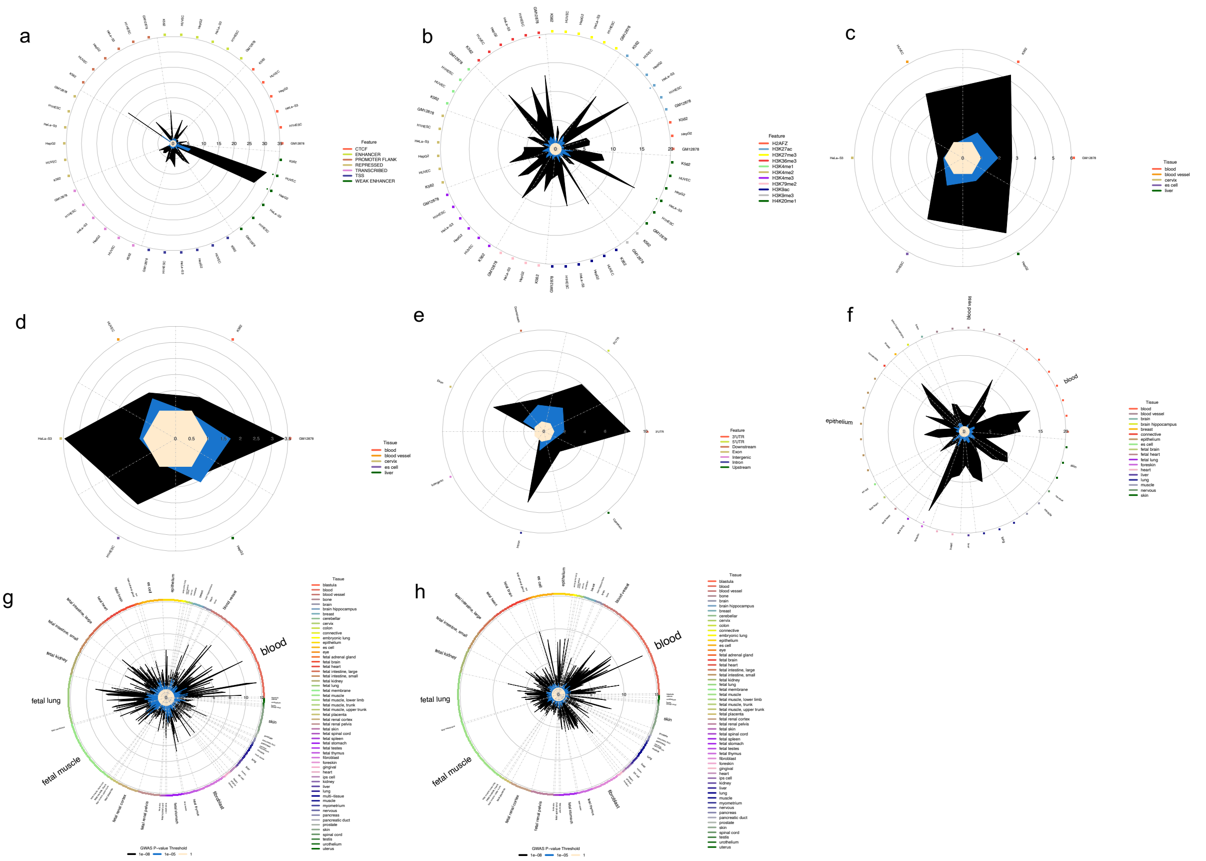

Annotations of associated SNPs to genic elements by Garfield[8].

Figure S5. Gene based association results.

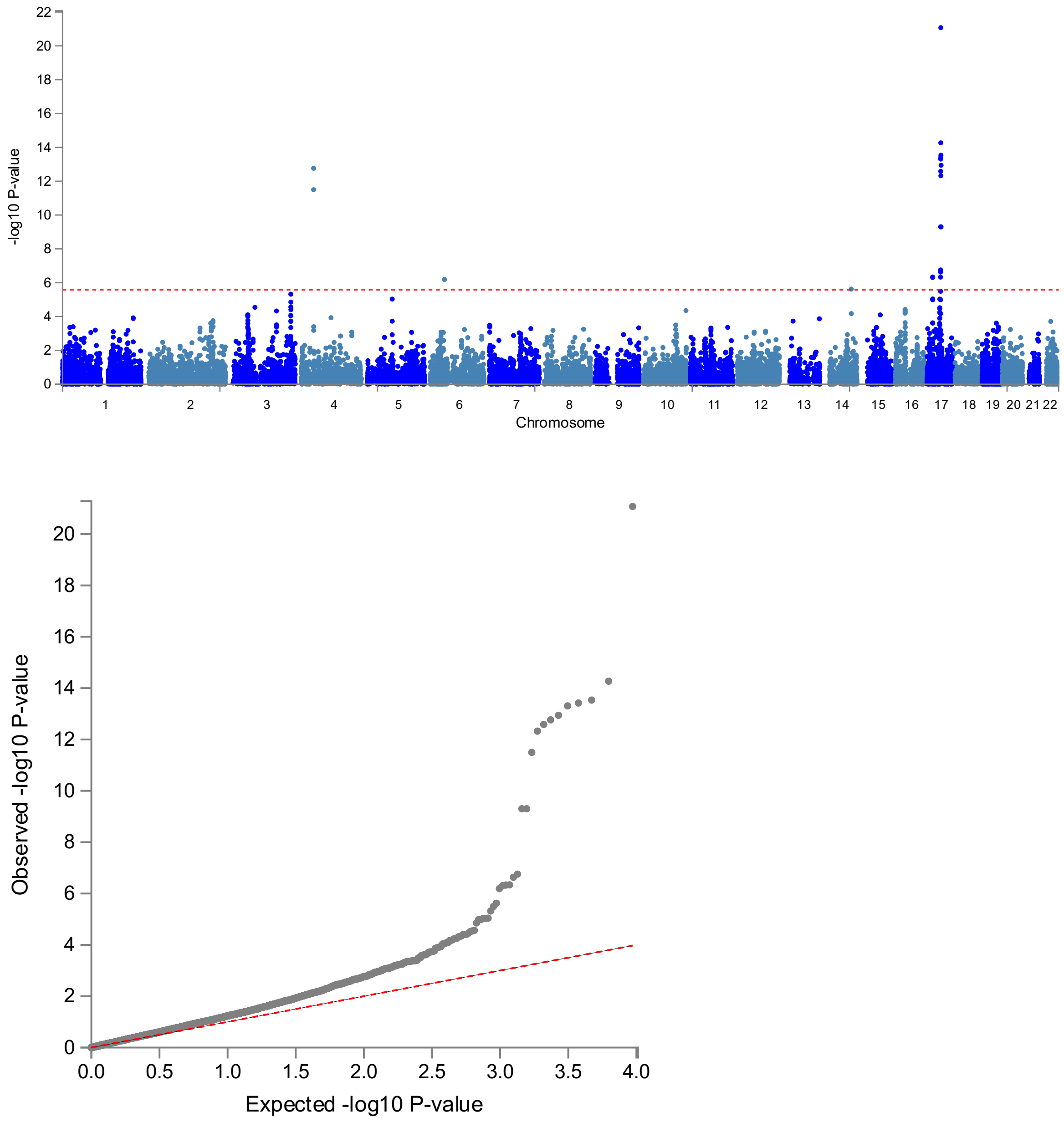

Manhattan plot and QQ plot for gene-based association statistics for BAG. Dashed line in the Manhattan plot indicates the genome wide significant threshold, and, in the QQ plot indicates the null association.

Figure S6. eQTL annotations for rs73185796.

Tissue	Samples	NES	p-value	m-value
Minor Salivary Gland	144	0.366	0.1	0.324
Esophagus - Mucosa	497	0.101	0.5	0.168
Stomach	324	0.0981	0.3	0.0270
Skin - Sun Exposed (Lower leg)	605	0.0977	0.4	0.188
Kidney - Cortex	73	0.0900	0.8	0.699
Adrenal Gland	233	0.0448	0.7	0.179
Prostate	221	0.0447	0.8	0.500
Whole Blood	670	-	-	-
Brain - Cerebellum	209	-0.0535	0.6	0.317
Colon - Sigmoid	318	-0.0670	0.6	0.511
Pituitary	237	-0.0684	0.6	0.588
Cells - EBV-transformed lymphocytes	147	-0.0799	0.7	0.628
Small Intestine - Terminal Ileum	174	-0.0828	0.5	0.558
Testis	322	-0.0908	0.4	0.579
Artery - Coronary	213	-0.0918	0.7	0.696
Brain - Anterior cingulate cortex (BA24)	147	-0.106	0.6	0.653
Adipose - Visceral (Omentum)	469	-0.129	0.3	0.730
Pancreas	305	-0.137	0.1	0.758
Breast - Mammary Tissue	396	-0.139	0.4	0.739
Liver	208	-0.146	0.5	0.707
Skin - Not Sun Exposed (Suprapubic)	517	-0.148	0.3	0.701
Cells - Cultured fibroblasts	483	-0.149	0.3	0.760
Uterus	129	-0.151	0.5	0.709
Colon - Transverse	368	-0.155	0.1	0.848
Heart - Left Ventricle	386	-0.156	0.3	0.766
Lung	515	-0.166	0.1	0.857
Spleen	227	-0.168	0.5	0.742
Ovary	167	-0.169	0.4	0.794
Thyroid	574	-0.171	0.1	0.805
Brain - Cerebellar Hemisphere	175	-0.178	0.1	0.889
Adipose - Subcutaneous	581	-0.217	0.1	0.938
Artery - Tibial	584	-0.232	0.06	0.956
Muscle - Skeletal	706	-0.247	0.01	0.968
Brain - Spinal cord (cervical c-1)	126	-0.277	0.006	0.987
Esophagus - Muscularis	465	-0.283	0.03	0.970
Artery - Aorta	387	-0.307	0.04	0.952
Brain - Caudate (basal ganglia)	194	-0.345	0.01	0.987
Brain - Hippocampus	165	-0.352	0.07	0.925
Heart - Atrial Appendage	372	-0.358	0.03	0.981
Brain - Hypothalamus	170	-0.363	0.02	0.959
Esophagus - Gastroesophageal Junction	330	-0.388	0.03	0.930
Brain - Putamen (basal ganglia)	170	-0.398	0.02	0.971
Brain - Nucleus accumbens (basal ganglia)	202	-0.486	4.2e-4	1.00
Vagina	141	-0.496	0.03	0.971
Nerve - Tibial	532	-0.565	1.9e-5	1.00
Brain - Amygdala	129	-0.572	0.007	0.956
Brain - Cortex	205	-0.671	8.4e-6	1.00
Brain - Substantia nigra	114	-0.680	7.2e-4	0.988
Brain - Frontal Cortex (BA9)	175	-0.738	2.2e-5	0.995

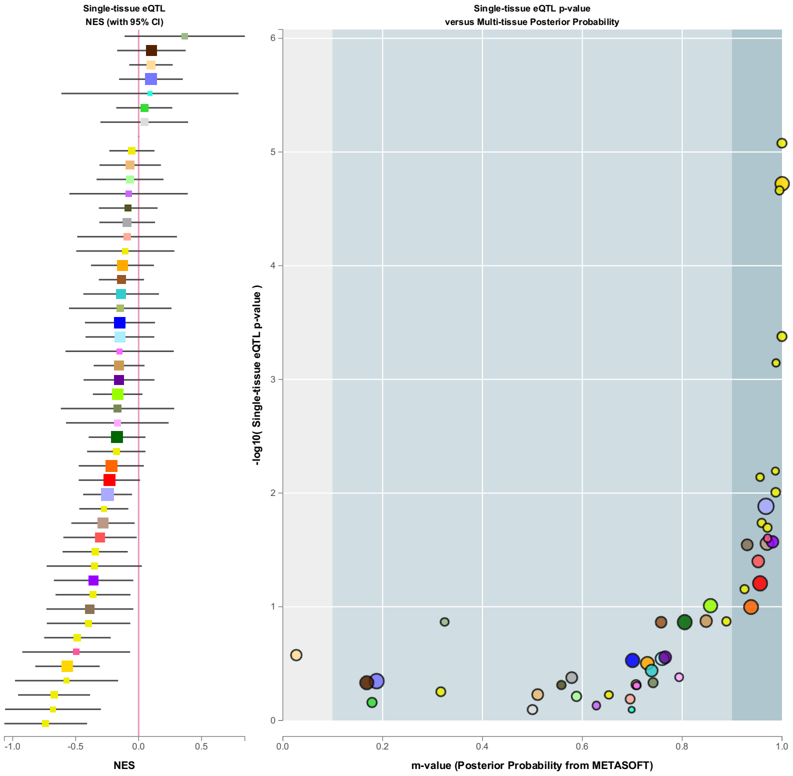

The SNP rs73185796 was search for in the GTEx v8 portal to verify whether it affects the expression level of the nearby gene, *VWA5B2*, in 49 tissues. The tissue types (Tissue), sample sizes (Samples), normalized effect sizes (NES), eQTL pvalues (p-value), and posterior probability that the SNP affect gene expression estimated by METASOFT[9] (m-value) and 95% CI were shown. Colors indicate tissue types. P-value <1.3x10-4 (0.05 / (49*8) is considered as the existence of statistical evidence; m-value >0.9 is additionally considered as the existence of eQTL. Supplementary Tables XXX include numeric values for other eGenes.

Figure S7. eQTL annotations for rs13132853.

Tissue	Samples	NES	p-value	m-value
Pituitary	237	0.172	0.02	0.175
Uterus	129	-0.00561	0.9	0.786
Adrenal Gland	233	-0.00985	0.9	0.784
Ovary	167	-0.0214	0.8	0.894
Thyroid	574	-0.0257	0.4	0.518
Liver	208	-0.0319	0.5	0.886
Colon - Sigmoid	318	-0.0448	0.3	0.829
Pancreas	305	-0.0496	0.4	0.903
Heart - Atrial Appendage	372	-0.0520	0.2	0.965
Small Intestine - Terminal Ileum	174	-0.0553	0.4	0.909
Brain - Amygdala	129	-0.0673	0.5	0.965
Artery - Coronary	213	-0.0690	0.2	0.977
Esophagus - Mucosa	497	-0.0692	0.05	0.948
Prostate	221	-0.0714	0.2	1.00
Colon - Transverse	368	-0.0758	0.09	0.946
Adipose - Visceral (Omentum)	469	-0.0772	0.05	0.993
Lung	515	-0.0782	0.03	0.983
Cells - Cultured fibroblasts	483	-0.0887	0.01	1.00
Breast - Mammary Tissue	396	-0.0892	0.03	1.00
Nerve - Tibial	532	-0.0913	0.01	1.00
Vagina	141	-0.0933	0.1	0.965
Esophagus - Muscularis	465	-0.0933	0.01	1.00
Brain - Frontal Cortex (BA9)	175	-0.0986	0.2	0.973
Brain - Nucleus accumbens (basal ganglia)	202	-0.106	0.1	0.996
Adipose - Subcutaneous	581	-0.114	0.006	1.00
Artery - Aorta	387	-0.114	3.9e-3	1.00
Spleen	227	-0.114	0.1	0.966
Brain - Anterior cingulate cortex (BA24)	147	-0.123	0.1	0.987
Brain - Hypothalamus	170	-0.124	0.09	0.950
Minor Salivary Gland	144	-0.129	0.2	0.978
Brain - Cortex	205	-0.129	0.09	0.987
Heart - Left Ventricle	386	-0.136	9.9e-4	1.00
Skin - Sun Exposed (Lower leg)	605	-0.136	6.8e-4	1.00
Brain - Hippocampus	165	-0.143	0.1	0.988
Testis	322	-0.144	3.5e-3	1.00
Esophagus - Gastroesophageal Junction	330	-0.147	2.2e-3	1.00
Artery - Tibial	584	-0.153	1.4e-5	1.00
Skin - Not Sun Exposed (Suprapubic)	517	-0.155	5.4e-4	1.00
Muscle - Skeletal	706	-0.158	1.4e-4	1.00
Brain - Caudate (basal ganglia)	194	-0.161	0.01	1.00
Brain - Substantia nigra	114	-0.162	0.1	0.982
Whole Blood	670	-0.168	9.1e-6	1.00
Cells - EBV-transformed lymphocytes	147	-0.168	0.1	0.982
Stomach	324	-0.177	5.9e-4	1.00
Kidney - Cortex	73	-0.191	0.08	0.974
Brain - Spinal cord (cervical c-1)	126	-0.198	0.04	0.993
Brain - Cerebellar Hemisphere	175	-0.236	0.006	1.00
Brain - Putamen (basal ganglia)	170	-0.308	6.4e-5	1.00
Brain - Cerebellum	209	-0.360	3.2e-5	1.00

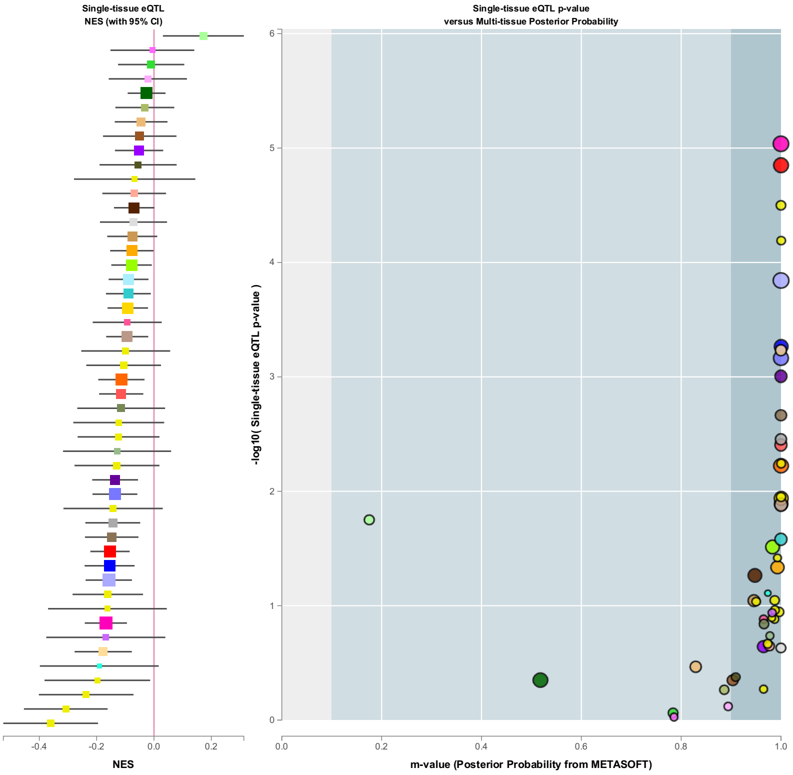

The SNP rs13132853 was search for in the GTEx v8 portal to verify whether it affects the expression level of the nearby gene, *KLF3*, in 49 tissues. The tissue types (Tissue), sample sizes (Samples), normalized effect sizes (NES), eQTL pvalues (p-value), and posterior probability that the SNP affect gene expression estimated by METASOFT[9] (m-value) and 95% CI were shown. Colors indicate tissue types. P-value <1.3x10-4 (0.05 / (49*8) is considered as the existence of statistical evidence; m-value >0.9 is additionally considered as the existence of eQTL. Supplementary Tables XXX include numeric values for other eGenes.

Figure S8. eQTL annotations for rs2790102.

Tissue	Samples	NES	p-value	m-value
Vagina	141	0.129	0.04	0.00
Pituitary	237	0.104	0.03	0.00
Adipose - Visceral (Omentum)	469	0.0921	0.02	0.00
Adrenal Gland	233	0.0663	0.4	0.00
Skin - Not Sun Exposed (Suprapubic)	517	0.0662	0.04	0.00
Liver	208	0.0583	0.4	0.00
Heart - Left Ventricle	386	0.0566	0.3	0.00
Small Intestine - Terminal Ileum	174	0.0553	0.2	0.00
Kidney - Cortex	73	0.0534	0.6	0.00
Colon - Sigmoid	318	0.0509	0.2	0.00
Pancreas	305	0.0490	0.3	0.00
Testis	322	0.0487	0.2	0.00
Esophagus - Gastroesophageal Junction	330	0.0404	0.4	0.00
Breast - Mammary Tissue	396	0.0349	0.2	0.00
Prostate	221	0.0319	0.4	0.00
Nerve - Tibial	532	0.0241	0.3	0.00
Esophagus - Mucosa	497	0.0239	0.5	0.00
Whole Blood	670	0.0182	0.4	0.00
Lung	515	0.0111	0.7	0.00
Muscle - Skeletal	706	0.00831	0.8	0.00
Heart - Atrial Appendage	372	0.00787	0.8	0.00
Artery - Tibial	584	0.00264	0.9	0.00
Stomach	324	-0.0102	0.7	0.00
Skin - Sun Exposed (Lower leg)	605	-0.0140	0.6	0.00
Minor Salivary Gland	144	-0.0224	0.7	0.00
Colon - Transverse	368	-0.0275	0.4	0.00
Adipose - Subcutaneous	581	-0.0368	0.2	0.00
Esophagus - Muscularis	465	-0.0431	0.4	0.00
Spleen	227	-0.0527	0.4	0.00
Cells - Cultured fibroblasts	483	-0.0564	0.08	0.00
Thyroid	574	-0.0584	0.05	0.00
Artery - Coronary	213	-0.0682	0.2	0.00
Brain - Anterior cingulate cortex (BA24)	147	-0.0884	0.4	0.0630
Artery - Aorta	387	-0.111	1.1e-3	0.0100
Uterus	129	-0.135	0.2	0.221
Brain - Nucleus accumbens (basal ganglia)	202	-0.164	0.01	0.453
Brain - Hypothalamus	170	-0.196	4.5e-3	0.883
Ovary	167	-0.200	1.0e-3	1.00
Brain - Cortex	205	-0.215	2.4e-3	0.994
Brain - Cerebellar Hemisphere	175	-0.231	0.007	0.936
Cells - EBV-transformed lymphocytes	147	-0.234	0.02	0.914
Brain - Frontal Cortex (BA9)	175	-0.251	9.1e-4	0.999
Brain - Amygdala	129	-0.285	1.4e-3	0.983
Brain - Substantia nigra	114	-0.286	4.3e-4	0.990
Brain - Spinal cord (cervical c-1)	126	-0.290	7.4e-6	1.00
Brain - Cerebellum	209	-0.302	2.3e-4	1.00
Brain - Hippocampus	165	-0.354	1.1e-6	1.00
Brain - Caudate (basal ganglia)	194	-0.389	6.2e-10	1.00
Brain - Putamen (basal ganglia)	170	-0.540	7.9e-12	1.00

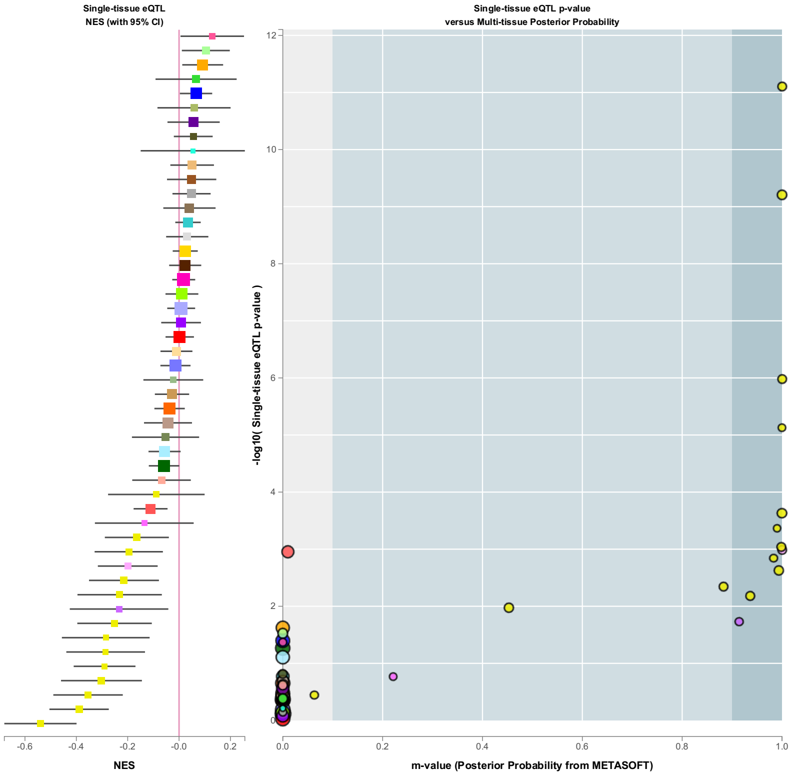

The SNP rs2790102 was search for in the GTEx v8 portal to verify whether it affects the expression level of the nearby gene, *RUNX2*, in 49 tissues. The tissue types (Tissue), sample sizes (Samples), normalized effect sizes (NES), eQTL pvalues (p-value), and posterior probability that the SNP affect gene expression estimated by METASOFT[9] (m-value) and 95% CI were shown. Colors indicate tissue types. P-value <1.3x10-4 (0.05 / (49*8) is considered as the existence of statistical evidence; m-value >0.9 is additionally considered as the existence of eQTL. Supplementary Tables XXX include numeric values for other eGenes.

Figure S9. eQTL and sQTL annotations for rs7461069.

Tissue	Samples	NES	p-value	m-value
Small Intestine - Terminal Ileum	174	0.219	0.01	0.841
Esophagus - Mucosa	497	0.198	7.5e-5	0.989
Minor Salivary Gland	144	0.112	0.2	0.367
Adrenal Gland	233	0.0937	0.2	0.316
Skin - Not Sun Exposed (Suprapubic)	517	0.0809	0.06	0.309
Skin - Sun Exposed (Lower leg)	605	0.0268	0.6	0.0720
Brain - Hypothalamus	170	0.0206	0.8	0.157
Vagina	141	0.00170	1	0.0820
Thyroid	574	-	-	-
Brain - Frontal Cortex (BA9)	175	-	-	-
Breast - Mammary Tissue	396	-	-	-
Adipose - Subcutaneous	581	-	-	-
Heart - Atrial Appendage	372	-	-	-
Brain - Amygdala	129	-	-	-
Stomach	324	-	-	-
Brain - Caudate (basal ganglia)	194	-	-	-
Colon - Transverse	368	-	-	-
Brain - Cerebellum	209	-	-	-
Esophagus - Muscularis	465	-	-	-
Pancreas	305	-	-	-
Nerve - Tibial	532	-	-	-
Spleen	227	-	-	-
Colon - Sigmoid	318	-	-	-
Brain - Anterior cingulate cortex (BA24)	147	-	-	-
Muscle - Skeletal	706	-	-	-
Brain - Hippocampus	165	-	-	-
Brain - Cortex	205	-	-	-
Heart - Left Ventricle	386	-	-	-
Artery - Tibial	584	-	-	-
Uterus	129	-	-	-
Ovary	167	-	-	-
Esophagus - Gastroesophageal Junction	330	-	-	-
Cells - EBV-transformed lymphocytes	147	-	-	-
Artery - Coronary	213	-	-	-
Brain - Spinal cord (cervical c-1)	126	-	-	-
Adipose - Visceral (Omentum)	469	-	-	-
Brain - Nucleus accumbens (basal ganglia)	202	-	-	-
Brain - Cerebellar Hemisphere	175	-	-	-
Cells - Cultured fibroblasts	483	-	-	-
Artery - Aorta	387	-	-	-
Brain - Putamen (basal ganglia)	170	-	-	-
Kidney - Cortex	73	-	-	-
Brain - Substantia nigra	114	-	-	-
Prostate	221	-0.00547	0.9	0.0660
Whole Blood	670	-0.0173	0.7	0.0190
Lung	515	-0.0418	0.5	0.0360
Pituitary	237	-0.0590	0.4	0.0290
Testis	322	-0.0660	0.3	0.0230
Liver	208	-0.140	0.04	0.0130

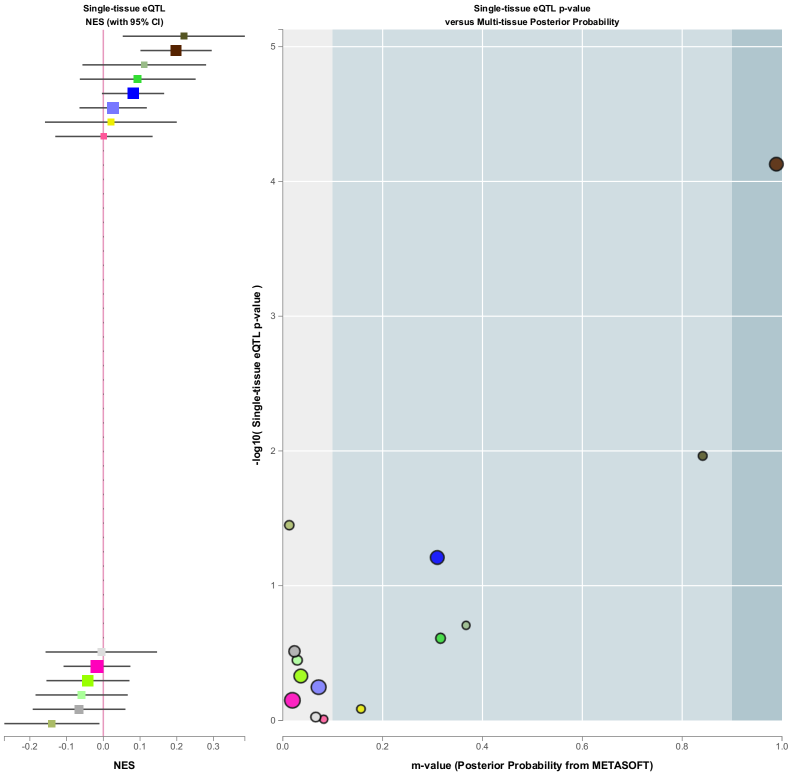

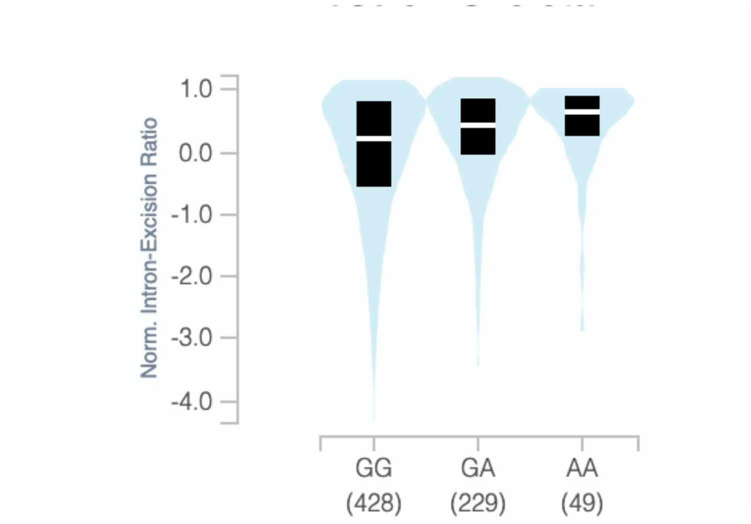

Top: The SNP rs2790102 was search for in the GTEx v8 portal to verify whether it affects the expression level of the nearby gene, *FAM83A-SA1*, in 49 tissues. The tissue types (Tissue), sample sizes (Samples), normalized effect sizes (NES), eQTL pvalues (p-value), and posterior probability that the SNP affect gene expression estimated by METASOFT[9] (m-value) and 95% CI were shown. Colors indicate tissue types. P-value <1.3x10-4 (0.05 / (49*8) is considered as the existence of statistical evidence; m-value >0.9 is additionally considered as the existence of eQTL.

Bottom: The SNP rs2790102 was annotated as splicing QTL to the expression of KLHL38 in the Muscle-skeletal tissue (p=9.7x10-6, NES=0.26).

Figure S10. eQTL and sQTL annotations for rs4880424.

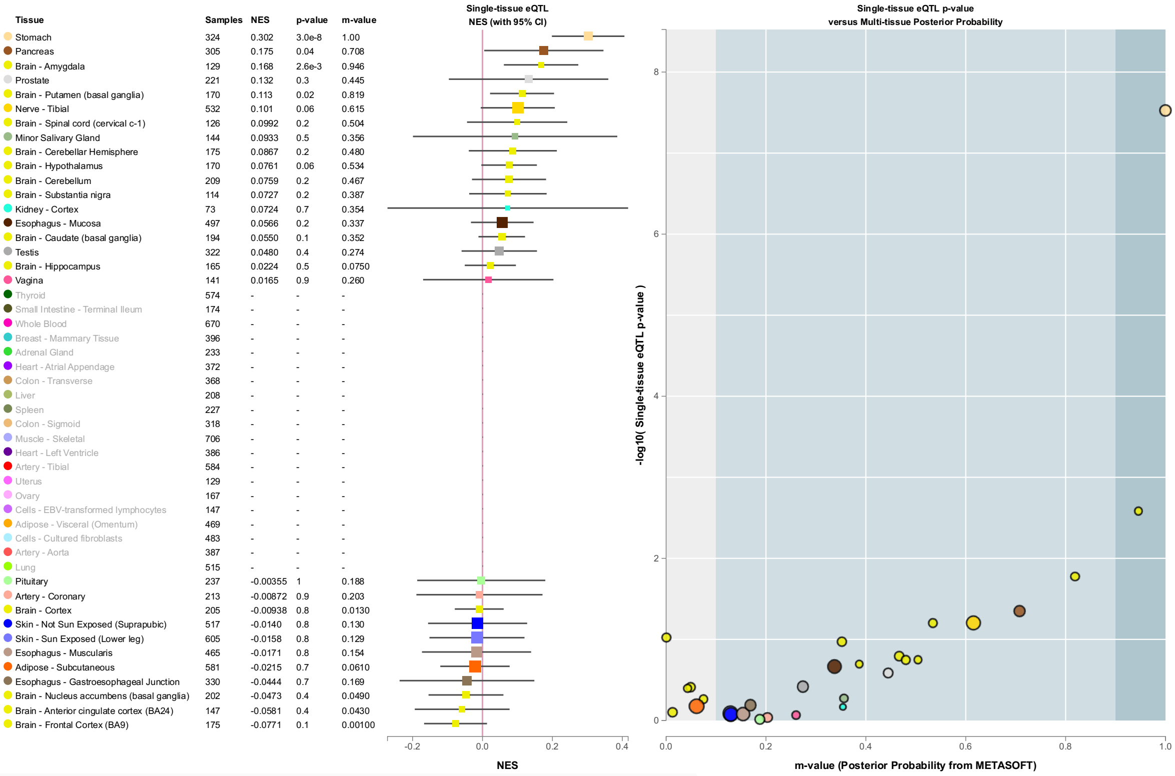

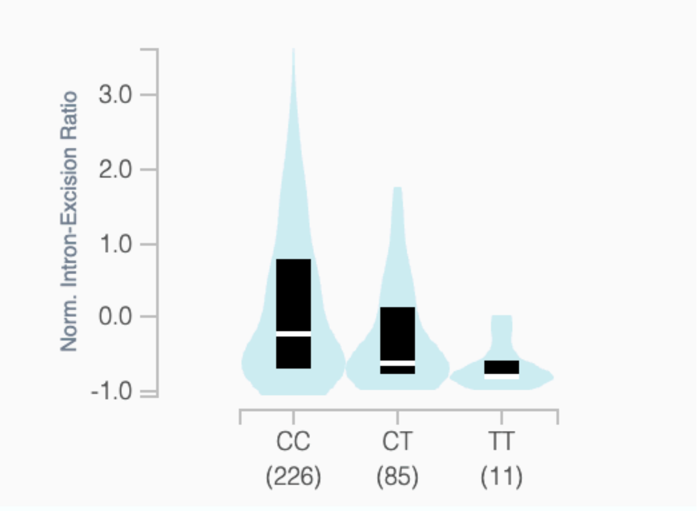

Top: The SNP rs4880424 was search for in the GTEx v8 portal to verify whether it affects the expression level of the nearby gene, *NKX6-2*, in 49 tissues. The tissue types (Tissue), sample sizes (Samples), normalized effect sizes (NES), eQTL pvalues (p-value), and posterior probability that the SNP affect gene expression estimated by METASOFT[9] (m-value) and 95% CI were shown. Colors indicate tissue types. P-value <1.3x10-4 (0.05 / (49*8) is considered as the existence of statistical evidence; m-value >0.9 is additionally considered as the existence of eQTL.

Bottom: The SNP rs4880424 was annotated as splicing QTL to the expression of INPP5A in the Testis tissue (p=6.0x10-7, NES=-0.48).

Figure S11. eQTL and sQTL annotations for rs17203398.

Tissue	Samples	NES	p-value	m-value
Artery - Tibial	584	0.402	3.3e-32	1.00
Kidney - Cortex	73	0.373	3.0e-3	0.974
Artery - Aorta	387	0.299	4.4e-14	1.00
Adipose - Subcutaneous	581	0.290	4.6e-16	1.00
Adrenal Gland	233	0.250	2.9e-6	1.00
Skin - Sun Exposed (Lower leg)	605	0.247	7.7e-12	1.00
Adipose - Visceral (Omentum)	469	0.222	7.1e-9	1.00
Esophagus - Muscularis	465	0.217	2.7e-10	1.00
Heart - Left Ventricle	386	0.213	2.1e-5	1.00
Breast - Mammary Tissue	396	0.209	1.4e-7	1.00
Skin - Not Sun Exposed (Suprapubic)	517	0.196	3.0e-7	1.00
Cells - Cultured fibroblasts	483	0.196	4.1e-10	1.00
Brain - Amygdala	129	0.194	5.7e-4	0.999
Esophagus - Gastroesophageal Junction	330	0.191	9.6e-6	1.00
Artery - Coronary	213	0.184	4.0e-5	1.00
Thyroid	574	0.182	1.3e-8	1.00
Colon - Sigmoid	318	0.171	2.8e-4	1.00
Brain - Anterior cingulate cortex (BA24)	147	0.166	1.7e-3	0.963
Heart - Atrial Appendage	372	0.165	2.8e-5	1.00
Muscle - Skeletal	706	0.155	1.0e-6	1.00
Uterus	129	0.143	0.1	0.769
Brain - Nucleus accumbens (basal ganglia)	202	0.135	4.2e-4	1.00
Colon - Transverse	368	0.134	1.9e-3	0.975
Minor Salivary Gland	144	0.134	0.04	0.801
Vagina	141	0.132	0.05	0.866
Stomach	324	0.123	0.02	0.838
Esophagus - Mucosa	497	0.109	3.2e-3	0.816
Prostate	221	0.0947	0.2	0.418
Brain - Frontal Cortex (BA9)	175	0.0939	0.09	0.355
Testis	322	0.0929	0.08	0.415
Brain - Cortex	205	0.0711	0.07	0.0460
Pituitary	237	0.0683	0.2	0.110
Pancreas	305	0.0496	0.3	0.0180
Brain - Substantia nigra	114	0.0431	0.4	0.0990
Nerve - Tibial	532	0.0427	0.1	0.00
Ovary	167	0.0397	0.4	0.00900
Brain - Caudate (basal ganglia)	194	0.0385	0.3	0.00600
Brain - Putamen (basal ganglia)	170	0.0278	0.5	0.00
Brain - Cerebellar Hemisphere	175	0.0234	0.7	0.0250
Brain - Hypothalamus	170	0.0195	0.7	0.00
Spleen	227	0.00602	0.9	0.00
Brain - Cerebellum	209	0.00204	1	0.0110
Whole Blood	670	0.000675	1	0.00
Brain - Hippocampus	165	-0.000279	1	0.00
Small Intestine - Terminal Ileum	174	-0.00892	0.9	0.00
Lung	515	-0.0204	0.6	0.00
Brain - Spinal cord (cervical c-1)	126	-0.0393	0.4	0.00
Cells - EBV-transformed lymphocytes	147	-0.138	0.2	0.00300
Liver	208	-0.375	7.6e-6	0.00

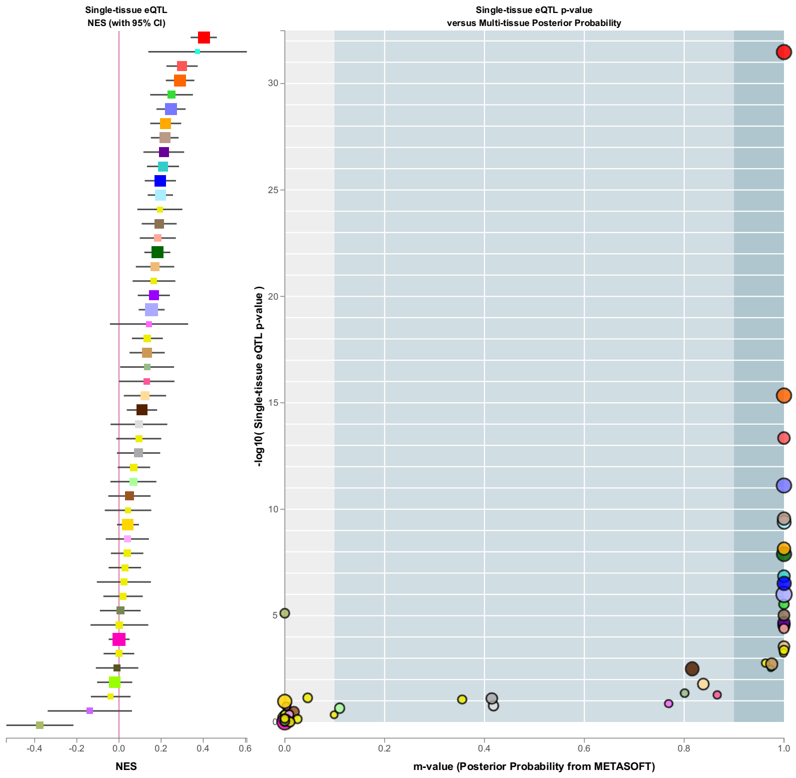

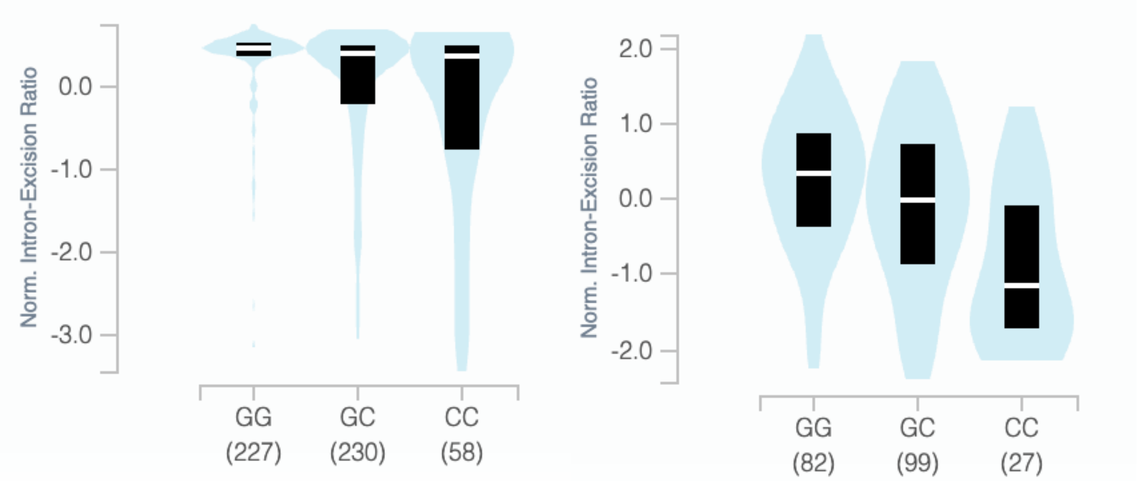

Top: The SNP rs17203398 was search for in the GTEx v8 portal to verify whether it affects the expression level of the nearby gene, *GALC*, in 49 tissues. The tissue types (Tissue), sample sizes (Samples), normalized effect sizes (NES), eQTL pvalues (p-value), and posterior probability that the SNP affect gene expression estimated by METASOFT[9] (m-value) and 95% CI were shown. Colors indicate tissue types. P-value <1.3x10-4 (0.05 / (49*8) is considered as the existence of statistical evidence; m-value >0.9 is additionally considered as the existence of eQTL. Supplementary Tables XXX include numeric values for other eGenes.
Bottom: The SNP rs17203398 was annotated as splicing QTL to the gene *GALC* in the Lung tissue (Left, p=2.7x10-8, NES=-0.27), and the non-coding RNA LINC01146 in the liver tissue (Right, p=3.6x10-7, NES=-0.46).

Figure S12. eQTL annotations for rs2106786.

Tissue	Samples	NES	p-value	m-value
Testis	322	1.22	3.7e-117	1.00
Esophagus - Mucosa	497	0.685	7.5e-73	1.00
Lung	515	0.594	3.4e-32	1.00
Skin - Sun Exposed (Lower leg)	605	0.491	1.1e-21	1.00
Spleen	227	0.464	1.3e-5	0.842
Vagina	141	0.418	2.2e-4	0.193
Skin - Not Sun Exposed (Suprapubic)	517	0.293	2.1e-9	0.00
Brain - Caudate (basal ganglia)	194	0.221	2.2e-4	0.00
Minor Salivary Gland	144	0.210	0.02	0.00
Brain - Putamen (basal ganglia)	170	0.175	0.02	0.00
Adipose - Visceral (Omentum)	469	0.172	1.0e-6	0.00
Adipose - Subcutaneous	581	0.123	3.5e-5	0.00
Breast - Mammary Tissue	396	0.107	0.01	0.00
Brain - Spinal cord (cervical c-1)	126	0.105	0.2	0.00
Nerve - Tibial	532	0.0869	0.08	0.00
Pituitary	237	0.0850	0.03	0.00
Artery - Coronary	213	0.0608	0.3	0.00
Brain - Anterior cingulate cortex (BA24)	147	0.0590	0.09	0.00
Adrenal Gland	233	0.0330	0.6	0.00
Brain - Nucleus accumbens (basal ganglia)	202	0.00728	0.9	0.00
Brain - Cortex	205	0.00618	0.8	0.00
Heart - Atrial Appendage	372	-0.000556	1	0.00
Brain - Amygdala	129	-0.000652	1	0.00
Brain - Substantia nigra	114	-0.00945	0.9	0.00
Brain - Hippocampus	165	-0.0254	0.4	0.00
Brain - Hypothalamus	170	-0.0371	0.2	0.00
Small Intestine - Terminal Ileum	174	-0.0460	0.4	0.00
Muscle - Skeletal	706	-0.0506	0.1	0.00
Brain - Frontal Cortex (BA9)	175	-0.0540	0.07	0.00
Liver	208	-0.0543	0.5	0.00
Kidney - Cortex	73	-0.0576	0.3	0.00
Pancreas	305	-0.0659	0.3	0.00
Whole Blood	670	-0.0791	0.08	0.00
Stomach	324	-0.102	0.03	0.00
Cells - Cultured fibroblasts	483	-0.103	0.009	0.00
Colon - Transverse	368	-0.172	2.3e-7	0.00
Cells - EBV-transformed lymphocytes	147	-0.179	0.3	0.00
Thyroid	574	-0.186	5.4e-5	0.00
Artery - Tibial	584	-0.194	6.8e-7	0.00
Esophagus - Muscularis	465	-0.206	1.1e-5	0.00
Esophagus - Gastroesophageal Junction	330	-0.209	1.3e-3	0.00
Ovary	167	-0.214	0.04	0.00
Brain - Cerebellar Hemisphere	175	-0.234	2.3e-4	0.00
Prostate	221	-0.276	8.2e-6	0.00
Brain - Cerebellum	209	-0.286	7.9e-7	0.00
Artery - Aorta	387	-0.324	3.7e-9	0.00
Uterus	129	-0.474	4.3e-4	0.00
Heart - Left Ventricle	386	-0.479	2.3e-16	0.00
Colon - Sigmoid	318	-0.669	5.4e-25	0.00

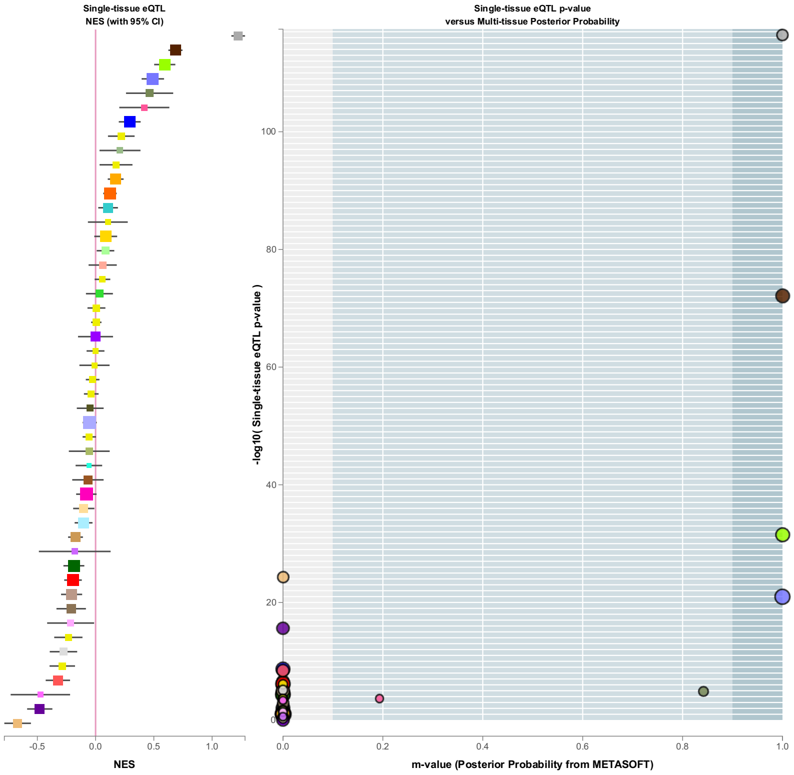

The SNP rs17203398 was search for in the GTEx v8 portal to verify whether it affects the expression level of the nearby gene, *MAPT*, in 49 tissues. The tissue types (Tissue), sample sizes (Samples), normalized effect sizes (NES), eQTL pvalues (p-value), and posterior probability that the SNP affect gene expression estimated by METASOFT[9] (m-value) and 95% CI were shown. Colors indicate tissue types. P-value <1.3x10-4 (0.05 / (49*8) is considered as the existence of statistical evidence; m-value >0.9 is additionally considered as the existence of eQTL. Supplementary Tables XXX include numeric values for other eGenes.

Figure S13. Conditional QQ plot for MDD vs BAG.

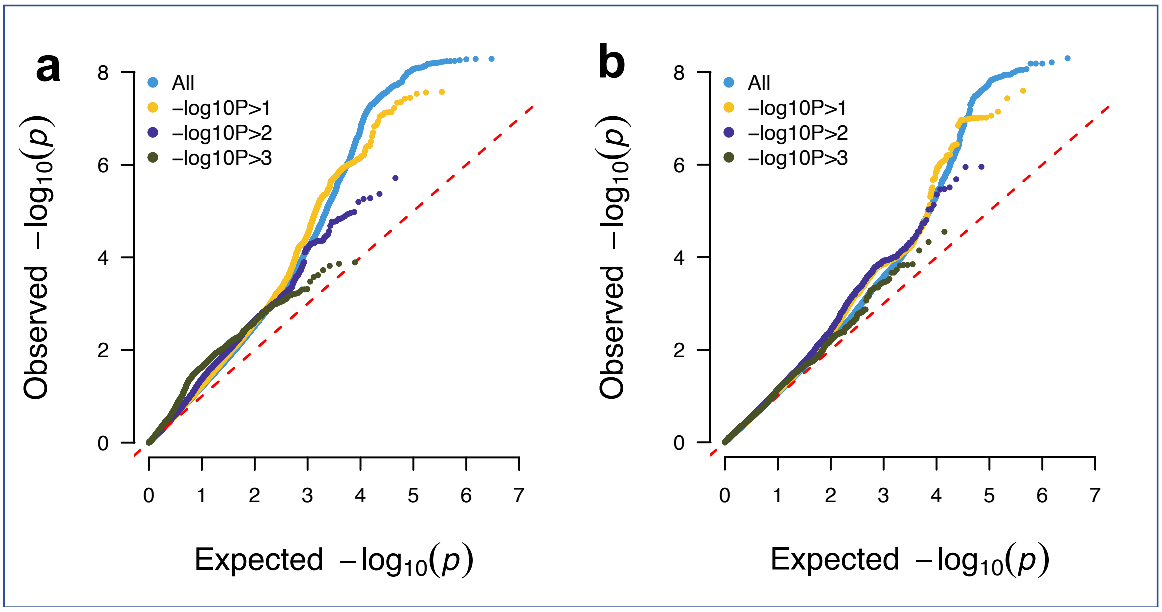

The conditional QQ plots for MDD condition on the levels of association for BAG (**a**), and for BAG condition on the level of association for MDD (**b**). As the association strength to the conditioned disorder increase, a successive leftward deflation in these curves indicates polygenic enrichment.

Figure S14. Conditional QQ plot for SCZ vs BAG.

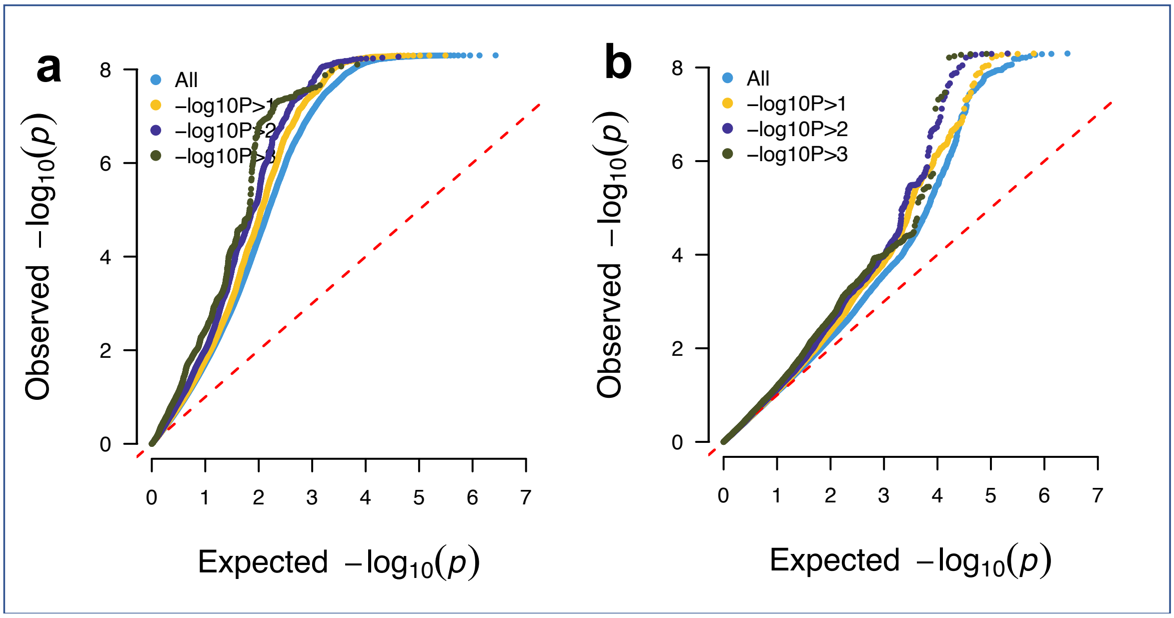

The conditional QQ plots for SCZ condition on the levels of association for BAG (**a**), and for BAG condition on the level of association for SCZ (**b**). As the association strength to the conditioned disorder increase, a successive leftward deflation in these curves indicates polygenic enrichment.

Figure S15. Conditional QQ plot for PD vs BAG excluding chr17.

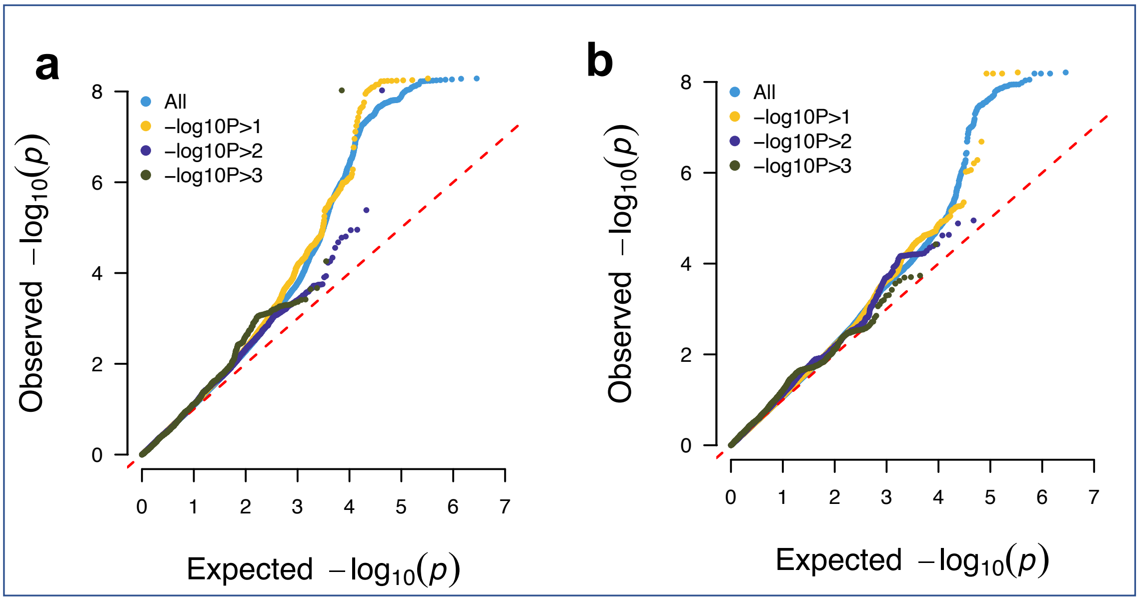

The conditional QQ plots for PD condition on the levels of association for BAG (**a**), and for BAG condition on the level of association for PD (**b**). As the association strength to the conditioned disorder increase, a successive leftward deflation in these curves indicates polygenic enrichment.

Table S1. Non-UK Biobank model training datasets.

Name	Size	Subset	Ages	Train
Autism Brain Imaging Data Exchange	555	Healthy Controls	6-56	356
Autism Brain Imaging Data Exchange II	184	Healthy Controls	8-64	118
ADHD 200	606	Healthy Controls	7-26	388
Amsterdam Open MRI Collection	819	ID1000	19-26	524
Beijing Normal University Enhanced Sample	180	Full	17-28	116
Cambridge Center for Aging and Neuroscience	653	Full	18-89	418
Consortium for Reliability and Reproducibility	1368	Full	6-84	880
Dallas Lifespan Brain Study	314	Full	21-89	201
ds000119	73	Full	8-27	47
ds000202	95	Full	18-30	61
ds000222	79	Full	21-73	51
1000 Functional Connectomes Project	812	Full	8-78	520
Healthy Brain Network	1855	Full	5-22	1188
Human Connectome Project	1113	Full	22-37	712
Max Planck Institute Leipzig Mind-Brain-Body	73	Full	22-68	47
Enhanced Nathan Kline Institute - Rockland Sample	928	Full	6-85	594
Open Access Series of Imaging Studies 3	1264	Healthy Controls	42-95	817
Pediatric, Imaging and Neurocognition	1174	Full	3-21	752
Southwest University Adult Lifespan Dataset	494	Full	19-80	316
Southwest University Longitudinal Imaging Multimodal	573	Full	17-27	367
**Total**	**13212**	-	**3-95**	**8473**

The dataset was compiled from 20 sources, spanning a wide age range and a multitude of scanners and scanning protocols. The number of participants from each source used for training is shown in the respective columns.

Table S2. Characteristics for the five folds data from UK Biobank.

Index	N	Age range (years)	Sex (F)	MAE(years)
Fold 0	5622	40-70	2907	2.49
Fold 1	5601	40-70	2885	2.44
Fold 2	5601	40-70	2888	2.45
Fold 3	5650	40-70	2914	2.44
Fold 4	5630	40-70	2907	2.44

Four folds were used for model training and tuning, and the trained model was used for predicting brain age for the hold-off fold. This procedure was iterated five times until all subjects have a brain age estimated from independent training models. Indexes corresponding to those used in Supplementary Figure 1; N, sample size; MAE, mean absolute error for predicted brain age.

Table S3. Statistics for lead SNPs of each associated region in each fold.

Fold	SNP	BP	A1	t-score	P
fold0	rs2790102	45432214	A	-2.54	1.12e-2
fold1	rs2790102	45432214	A	-1.95	5.09e-2
fold2	rs2790102	45432214	A	-3.56	3.77e-4
fold3	rs2790102	45432214	A	-1.86	6.36e-2
fold4	rs2790102	45432214	A	-2.97	2.98e-3
fold0	rs4880424	134584577	T	3.76	1.7e-4
fold1	rs4880424	134584577	T	3.16	1.61e-3
fold2	rs4880424	134584577	T	3.12	1.79e-3
fold3	rs4880424	134584577	T	-0.54	0.59
fold4	rs4880424	134584577	T	2.75	5.97e-3
fold0	rs17203398	88449847	C	-4.13	3.71e-5
fold1	rs17203398	88449847	C	-1.37	0.17
fold2	rs17203398	88449847	C	-4.35	1.41e-5
fold3	rs17203398	88449847	C	-2.04	4.19e-2
fold4	rs17203398	88449847	C	-2.44	1.47e-2
fold0	rs2106786	43919096	G	3.72	2.05e-4
fold1	rs2106786	43919096	G	4.46	8.42e-6
fold2	rs2106786	43919096	G	4.45	8.59e-6
fold3	rs2106786	43919096	G	5.50	4.01e-8
fold4	rs2106786	43919096	G	4.22	2.52e-5
fold0	rs73185796	183975709	T	-1.21	0.23
fold1	rs73185796	183975709	T	-2.84	4.52e-3
fold2	rs73185796	183975709	T	-3.39	6.93e-4
fold3	rs73185796	183975709	T	-2.32	2.02e-2
fold4	rs73185796	183975709	T	-2.72	6.57e-3
fold0	rs13132853	38680015	G	3.59	3.32e-4
fold1	rs13132853	38680015	G	4.18	2.96e-5
fold2	rs13132853	38680015	G	4.29	1.82e-5
fold3	rs13132853	38680015	G	4.10	4.16e-5
fold4	rs13132853	38680015	G	3.378	7.35e-4
fold0	rs79107704	78388694	A	2.26	2.38e-2
fold1	rs79107704	78388694	A	1.64	0.10
fold2	rs79107704	78388694	A	3.27	1.1e-3
fold3	rs79107704	78388694	A	2.92	3.57e-3
fold4	rs79107704	78388694	A	2.49	1.29e-2
fold0	rs7461069	124669029	A	-3.10	1.94e-3
fold1	rs7461069	124669029	A	-2.64	8.37e-3
fold2	rs7461069	124669029	A	-1.47	0.14
fold3	rs7461069	124669029	A	-2.04	4.13e-2
fold4	rs7461069	124669029	A	-3.39	7.12e-4

BP: base pair position on the hg19 build; A1: effective allele; t-score: effect size divided by standard error; P: association p values. Numeric values were rounded to two decimal places.

Table S4. Statistics for lead SNPs of each associated region in each replication samples.

a.

CHR	BP	SNP	A1	N	t-score	P
4	38680015	rs13132853	G	702	0.26	0.79
6	45432214	rs2790102	A	702	-0.13	0.90
8	124669029	rs7461069	A	702	-2.30	2.19e-2
10	134584577	rs4880424	T	702	-0.72	0.47
14	88449847	rs17203398	C	702	-2.70	7.17e-3
17	43919096	rs2106786	G	702	0.86	0.39

b.

CHR	BP	SNP	A1	N	t-score	P
3	183975709	rs73185796	T	321	0.88	0.38
4	38680015	rs13132853	G	321	-0.44	0.66
6	45432214	rs2790102	A	321	-1.06	0.29
8	124669029	rs7461069	A	321	0.19	0.85
10	134584577	rs4880424	T	321	-0.19	0.85
14	88449847	rs17203398	C	321	0.16	0.87
17	43919096	rs2106786	G	321	0.52	0.60

c.

CHR	BP	SNP	A1	N	t-score	P
3	183975709	rs73185796	T	608	-1.73	8.50e-2
4	38680015	rs13132853	G	600	-0.44	0.66
5	78388694	rs79107704	A	612	1.11	0.27
6	45432214	rs2790102	A	501	-1.22	0.22
8	124669029	rs7461069	A	595	1.004	0.32
10	134584577	rs4880424	T	602	0.35	0.72
14	88449847	rs17203398	C	611	0.66	0.51

a. ADNI I sample; **b.** ADNI II sample; **c.** Local healthy control sample.

The ADNI samples I and II were impudently imputed on two the 1000 Genomes Project Phases3 reference data using the software Genipe[3].

CHR: chromosome number; BP: base pair position on the hg19 build; A1: effective allele; N, number of subjects analyzed; t-score: effect size divided by standard error; P: association p values. Numeric values were rounded to two decimal places.

Table S5 Function annotation for genes in Figure 1c.

1. Symbol: **BHMT2**; Name: **Betaine--Homocysteine S-Methyltransferase 2**

Homocysteine is a sulfur-containing amino acid that plays a crucial role in methylation reactions. Transfer of the methyl group from betaine to homocysteine creates methionine, which donates the methyl group to methylate DNA, proteins, lipids, and other intracellular metabolites. The protein encoded by this gene is one of two methyl transferases that can catalyze the transfer of the methyl group from betaine to homocysteine. Anomalies in homocysteine metabolism have been implicated in disorders ranging from vascular disease to neural tube birth defects such as spina bifida, Orofacial Cleft. Among its related pathways are Metabolism and Sulfur amino acid metabolism.

2. Symbol: **AP2M1**; Name: **Adaptor Related Protein Complex 2 Subunit Mu 1**

This gene encodes a subunit of the heterotetrameric coat assembly protein complex 2 (AP2), which belongs to the adaptor complexes medium subunits family. The encoded protein is required for the activity of a vacuolar ATPase, which is responsible for proton pumping occurring in the acidification of endosomes and lysosomes. The encoded protein may also play an important role in regulating the intracellular trafficking and function of CTLA-4 protein. Among its related pathways are Arf1 pathway and Metabolism. Adaptor protein complexes function in protein transport via transport vesicles in different membrane traffic pathways. AP-2 is involved in clathrin-dependent endocytosis in which cargo proteins are incorporated into vesicles surrounded by clathrin (clathrin-coated vesicles, CCVs) which are destined for fusion with the early endosome. Clathrin-associated adaptor protein (AP) complexes which can bind directly to both the clathrin lattice and to the lipid and protein components of membranes are considered to be the major clathrin adaptors contributing the CCV formation. AP-2 may also play a role in maintaining normal post-endocytic trafficking through the ARF6-regulated, non-clathrin pathway. During long-term potentiation in hippocampal neurons, AP-2 is responsible for the endocytosis of ADAM10. Diseases associated with AP2M1 include *Intellectual Developmental Disorder*, *Autosomal Dominant 60*, *Seizures* and *Epilepsy*, *Myoclonic-Atonic Seizures*.

3. Symbol: **PSMD2**; Name: **Proteasome 26S Subunit Ubiquitin Receptor, Non-ATPase 2**

The 26S proteasome is a multicatalytic proteinase complex with a highly ordered structure composed of 2 complexes, a 20S core and a 19S regulator. Proteasomes are distributed throughout eukaryotic cells at a high concentration and cleave peptides in an ATP/ubiquitin-dependent process in a non-lysosomal pathway. An essential function of a modified proteasome, the immunoproteasome, is the processing of class I MHC peptides. This gene encodes one of the non-ATPase subunits of the 19S regulator lid. In addition to participation in proteasome function, this subunit may also participate in the TNF signalling pathway since it interacts with the tumor necrosis factor type 1 receptor. This complex plays a key role in the maintenance of protein homeostasis by removing misfolded or damaged proteins, which could impair cellular functions, and by removing proteins whose functions are no longer required. Therefore, the proteasome participates in numerous cellular processes, including cell cycle progression, apoptosis, or DNA damage repair.

4. Symbol: **HEXIM1**; Name: **HEXIM P-TEFb Complex Subunit 1**

Expression of this gene is induced by hexamethylene-bis-acetamide in vascular smooth muscle cells. Among its related pathways are Initiation of transcription and translation elongation at the HIV-1 LTR and Chromatin Regulation/Acetylation. It is a transcriptional regulator which functions as a general RNA polymerase II transcription inhibitor. Core component of the 7SK RNP complex: in cooperation with 7SK snRNA sequesters P-TEFb in a large inactive 7SK snRNP complex preventing RNA polymerase II phosphorylation and subsequent transcriptional elongation. It may also regulate NF-kappa-B, ESR1, NR3C1 and CIITA-dependent transcriptional and plays a role in the regulation of DNA virus-mediated innate immune response by assembling into the HDP-RNP complex, a complex that serves as a platform for IRF3 phosphorylation and subsequent innate immune response activation through the cGAS-STING pathway. Diseases associated with HEXIM1 include *Nut Midline Carcinoma* and *Immune Deficiency Disease*.

5. Symbol: **EIF2B5**; Name: **Eukaryotic Translation Initiation Factor 2B Subunit Epsilon**

This gene encodes one of five subunits of eukaryotic translation initiation factor 2B (EIF2B), a GTP exchange factor for eukaryotic initiation factor 2 and an essential regulator for protein synthesis. Among its related pathways are Translational Control and Peptide chain elongation. It catalyzes the exchange of eukaryotic initiation factor 2-bound GDP for GTP. Mutations in this gene and the genes encoding other EIF2B subunits have been associated with *leukoencephalopathy* with vanishing white matter.

6. Symbol: **NMT1**; Name: **N-Myristoyltransferase 1**

Myristate, a rare 14-carbon saturated fatty acid, is cotranslationally attached by an amide linkage to the N-terminal glycine residue of cellular and viral proteins with diverse functions. N-myristoyltransferase catalyzes the transfer of myristate from CoA to proteins. N-myristoylation appears to be irreversible and is required for full expression of the biologic activities of several N-myristoylated proteins, including the alpha subunit of the signal-transducing guanine nucleotide-binding protein (G protein). Among its related pathways are Activation of BH3-only proteins and HIV Life Cycle. It adds a myristoyl group to the N-terminal glycine residue of certain cellular and viral proteins. Diseases associated with NMT1 include *Gallbladder Cancer* and *Noonan Syndrome 11*.

7. Symbol: **DVL3**; Name: **Dishevelled Segment Polarity Protein 3**

This gene is a member of a multi-gene family which shares strong similarity with the Drosophila dishevelled gene, dsh. The Drosophila dishevelled gene encodes a cytoplasmic phosphoprotein that regulates cell proliferation. It also Involved in the signal transduction pathway mediated by multiple Wnt genes.

8. Symbol: **PARL**; Name: **Presenilin Associated Rhomboid Like**

This gene encodes a member of the rhomboid family of intramembrane serine proteases that is localized to the inner mitochondrial membrane. The encoded protein regulates mitochondrial remodeling and apoptosis through regulated substrate proteolysis. Proteolytic processing of the encoded protein results in the release of a small peptide, P-beta, which may transit to the nucleus. This protein is required for the control of apoptosis during postnatal growth and promotes changes in mitochondria morphology regulated by phosphorylation of P-beta domain. Mutations in this gene may be associated with *Parkinson's disease*.

9. Symbol: **DCAKD**; Name: **Dephospho-CoA Kinase Domain Containing**

The protein coded by this gene is predicted to enable dephospho-CoA kinase activity and involved in coenzyme A biosynthetic process. Gene Ontology (GO) annotations related to this gene include dephospho-CoA kinase activity.

10. Symbol: **INPP5A**; Name: **Inositol Polyphosphate-5-Phosphatase A**

The protein encoded by this gene is a membrane-associated type I inositol 1,4,5-trisphosphate (InsP3) 5-phosphatase. InsP3 5-phosphatases hydrolyze Ins(1,4,5)P3, which mobilizes intracellular calcium and acts as a second messenger mediating cell responses to various stimulation. Among its related pathways are Metabolism and superpathway of D-myo-inositol (1,4,5)-trisphosphate metabolism. Diseases associated with INPP5A include *Alternating Esotropia* and *Lowe Oculocerebrorenal Syndrome*.

11. Symbol: **FAM114A1**; Name: **Family With Sequence Similarity 114 Member A1**

The protein encoded by this gene belongs to the FAM114 family and may play a role in *neuronal cell development*. It is a phosphatase that specifically hydrolyzes the 5-phosphate of inositol 1,4,5-trisphosphate to inositol 1,4-bisphosphate, and inositol 1,3,4,5-tetrasphosphate to inositol 1,3,4-trisphosphate. It also may play a crucial role in the survival of cerebellar Purkinje cells.

12. Symbol: **ALG3**; Name: **ALG3 Alpha-1,3- Mannosyltransferase**

This gene encodes a member of the ALG3 family. The encoded protein catalyses the addition of the first dol-P-Man derived mannose in an alpha 1,3 linkage to Man5GlcNAc2-PP-Dol. Defects in this gene have been associated with c*ongenital disorder of glycosylation type Id* (CDG-Id) characterized by abnormal N-glycosylation, and Congenital Disorders Of N-Linked Glycosylation. Among its related pathways are Synthesis of substrates in N-glycan biosythesis and Metabolism of proteins.

14. Symbol: **KLF3**; Name: **Kruppel Like Factor 3**

The protein coded by this gene enables sequence-specific double-stranded DNA binding activity. It is predicted to be involved in regulation of transcription by RNA polymerase II. The protein binds to the CACCC box of erythroid cell-expressed genes and may play a role in hematopoiesis.

15. Symbol: **ARHGAP27**; Name: **Rho GTPase Activating Protein 27**

This gene encodes a member of a large family of proteins that activate Rho-type guanosine triphosphate (GTP) metabolizing enzymes. The encoded protein may pay a role in clathrin-mediated endocytosis. GTPase activators for the Rho-type GTPases act by converting them to an inactive GDP-bound state.

16. Symbol: **ABCC5**; Name: **ATP Binding Cassette Subfamily C Member 5**

The protein encoded by this gene is a member of the superfamily of ATP-binding cassette (ABC) transporters. ABC proteins transport various molecules across extra- and intra-cellular membranes. This protein is a member of the MRP subfamily which is involved in multi-drug resistance. It functions in the cellular export of its substrate, cyclic nucleotides. This export contributes to the degradation of phosphodiesterases and possibly an elimination pathway for cyclic nucleotides. Studies show that this protein provides resistance to thiopurine anticancer drugs, 6-mercatopurine and thioguanine, and the *anti-HIV drug 9*-(2-phosphonylmethoxyethyl) adenine. Among its related pathways are Metabolism and Glycosaminoglycan metabolism. It acts as a heme transporter required for the translocation of cytosolic heme to the secretory pathway and may play a role in energy metabolism by regulating the glucagon-like peptide 1 (GLP-1) secretion from enteroendocrine cells. This protein may be involved in resistance to thiopurines in *acute lymphoblastic leukemia* and *antiretroviral* nucleoside analogs in HIV-infected patients. Diseases associated with ABCC5 include *Primary Angle-Closure Glaucoma* and *Episodic Kinesigenic Dyskinesia 1*.

17. Symbol: **NSF**; Name: **N-Ethylmaleimide Sensitive Factor, Vesicle Fusing ATPase**

The protein coded by this gene enables PDZ domain binding activity and ionotropic glutamate receptor binding activity. It is involved in intracellular protein transport, positive regulation of protein catabolic process, and positive regulation of receptor recycling. Among its related pathways are Neuroscience and Vesicle-mediated transport. Diseases associated with NSF include *Developmental And Epileptic Encephalopathy 96* and *Tetanus*.

18. Symbol: **FMNL1**; Name: **Formin Like 1**

This gene encodes a formin-related protein. Formin-related proteins have been implicated in morphogenesis, cytokinesis, and cell polarity. The protein may play a role in the control of cell motility, survival of macrophages, the regulation of cell morphology and cytoskeletal organization. It is required in the cortical actin filament dynamics and cell shape.

19. Symbol: **ACBD4**; Name: **Acyl-CoA Binding Domain Containing 4**

This gene encodes a member of the acyl-coenzyme A binding domain containing protein family. All family members contain the conserved acyl-Coenzyme A binding domain, which binds acyl-CoA thiol esters. They are thought to play roles in acyl-CoA dependent lipid metabolism by binding to medium- and long-chain acyl-CoA esters and may function as an intracellular carrier of acyl-CoA esters.

20. Symbol: **GALC**; Name: **Galactosylceramidase**

This gene encodes a lysosomal protein which hydrolyzes the galactose ester bonds of galactosylceramide, galactosylsphingosine, lactosylceramide, and monogalactosyldiglyceride. Enzyme with very low activity responsible for the lysosomal catabolism of galactosylceramide, a major lipid in myelin, kidney and epithelial cells of small intestine and colon. Mutations in this gene have been associated with *Krabbe disease*, also known as globoid cell leukodystrophy.

21. Symbol: **KANSL1**; Name: **KAT8 Regulatory NSL Complex Subunit 1**

This gene encodes a nuclear protein that is a subunit of two protein complexes involved with histone acetylation, the MLL1 complex and the NSL1 complex. As part of the NSL complex it is involved in acetylation of nucleosomal histone H4 on several lysine residues and therefore may be involved in the regulation of transcription.

22. Symbol: **PLEKHM1**; Name: **Pleckstrin Homology And RUN Domain Containing M1**
The protein encoded by this gene is essential for bone resorption, and may play a critical role in vesicular transport in the osteoclast. It acts as a multivalent adapter protein that regulates Rab7-dependent and HOPS complex-dependent fusion events in the endolysosomal system and couples autophagic and the endocytic trafficking pathways. PLEKGN1 is a dual effector of RAB7A and ARL8B that simultaneously binds these GTPases, bringing about clustering and fusion of late endosomes and lysosomes. It is required for late stages of endolysosomal maturation, facilitating both endocytosis-mediated degradation of growth factor receptors and autophagosome clearance. For example, in case of infection, it contributes to Salmonella typhimurium pathogenesis by supporting the integrity of the Salmonella-containing vacuole (SCV) probably in concert with the HOPS complex and Rab7. Mutations in this gene are associated with *autosomal recessive osteopetrosis type 6* (OPTB6).
23. Symbol: **FBXO32**; Name: **F-Box Protein 32**
This gene encodes a member of the F-box protein family which is characterized by an approximately 40 amino acid motif, the F-box. The F-box proteins constitute one of the four subunits of the ubiquitin protein ligase complex called SCFs (SKP1-cullin-F-box), which function in phosphorylation-dependent ubiquitination. The protein probably recognizes and binds to phosphorylated target proteins during skeletal muscle atrophy. It is highly expressed during *muscle atrophy*, whereas mice deficient in this gene were found to be resistant to atrophy. This protein is thus a potential drug target for the treatment of *muscle atrophy.*
24. Symbol: **PLCD3**; Name: **1-Phosphatidylinositol 4,5-Bisphosphate Phosphodiesterase Delta-3**
This gene encodes a member of the phospholipase C family, which catalyze the hydrolysis of phosphatidylinositol 4,5-bisphosphate to generate the second messengers diacylglycerol and inositol 1,4,5-trisphosphate (IP3). Diacylglycerol and IP3 mediate a variety of cellular responses to extracellular stimuli by inducing protein kinase C and increasing cytosolic Ca(2+) concentrations. Its activity is inhibited by spermine, sphingosine, and several phospholipids. It is essential for trophoblast and placental development and may participate in cytokinesis by hydrolyzing PIP2 at the cleavage furrow; It also regulates *neurite outgrowth* through the inhibition of RhoA/Rho kinase signaling.

GROUP 2
25. Symbol: **TLR10**; Name: **Toll Like Receptor 10**
The protein encoded by this gene is a member of the Toll-like receptor (TLR) family which plays a fundamental role in pathogen recognition and activation of innate immunity. TLRs are highly conserved from Drosophila to humans and share structural and functional similarities. They recognize pathogen-associated molecular patterns (PAMPs) that are expressed on infectious agents, and mediate the production of cytokines necessary for the development of *effective immunity*. It acts via MYD88 and TRAF6, leading to NF-kappa-B activation, cytokine secretion and the inflammatory response.
26. Symbol: **GPR65**; Name: **G Protein-Coupled Receptor 65**
The protein encoded by this gene enables G protein-coupled receptor activity and is involved in several processes, including actin cytoskeleton reorganization, activation of GTPase activity, and positive regulation of stress fiber assembly. It is a receptor for the glycosphingolipid psychosine (PSY) and several related glycosphingolipids. It also plays a role in *immune response* by maintaining lysosome function and supporting phagocytosis-mediated intracellular *bacteria clearance* and may have a role in activation-induced cell death or differentiation of T-cells.
27. Symbol: **TLR1**; Name: **Toll Like Receptor 1**
The protein encoded by this gene is a member of the Toll-like receptor (TLR) family which plays a fundamental role in pathogen recognition and activation of innate immunity. This gene is ubiquitously expressed, and at higher levels than other TLR genes. This protein participates in the innate immune response to microbial agents, specifically recognizes diacylated and triacylated lipopeptides. It cooperates with TLR2 to mediate the innate immune response to bacterial

lipoproteins or lipopeptides by forming the activation cluster TLR2:TLR1:CD14 in response to triacylated lipopeptides. This cluster triggers signaling from the cell surface and subsequently is targeted to the Golgi in a lipid-raft dependent pathway, acting via MYD88 and TRAF6, leading to NF-kappa-B activation, cytokine secretion and the inflammatory response.

28. Symbol: **KLHL38**; Name: **Kelch Like Family Member 38**

KLHL38 is a Protein Coding gene. Diseases associated with KLHL38 include *Posterior Myocardial Infarction.*

29. Symbol: **SPATA32**; Name: **Spermatogenesis Associated 32**

The protein encoded by this gene is predicted to enable actin binding activity, to be involved in spermatogenesis and active in perinuclear region of cytoplasm.

30. Symbol: **C17orf104**

This is an RNA Gene and is affiliated with the lncRNA class; Its function is unclear.

31. Symbol: **SPPL2C**; Name: **Signal Peptide Peptidase Like 2C**

The protein encoded by this gene enables protein homodimerization activity. And, it is predicted to be involved in membrane protein proteolysis. SPPL2C is integral component of cytoplasmic side of endoplasmic reticulum membrane and integral component of lumenal side of endoplasmic reticulum membrane. Diseases associated with SPPL2C include *chromosome 17Q21.31 Duplication Syndrome* and *Caplan's Syndrome*.

32. Symbol: **LRRC37A**; Name: **Leucine Rich Repeat Containing 37A**

The protein encoded by this gene is predicted to be integral component of membrane. Diseases associated with LRRC37A include *Koolen-De Vries Syndrome* and *Supranuclear Palsy, Progressive, 1*.

33. Symbol: **ARL17A**; Name: **ADP Ribosylation Factor Like GTPase 17A**

The protein encoded by this gene is predicted to enable GTP binding activity, involved in intracellular protein transport and vesicle-mediated transport. It functions as an allosteric activator of the cholera toxin catalytic subunit, an ADP-ribosyltransferase. Diseases associated with ARL17A include *Bardet-Biedl Syndrome* and *Retinitis Pigmentosa*.

34. Symbol: **ARL17B**; Name: **ADP Ribosylation Factor Like GTPase 17B**

The protein encoded by this gene is predicted to enable GTP binding activity, involved in intracellular protein transport and vesicle-mediated transport. It functions as an allosteric activator of the cholera toxin catalytic subunit, an ADP-ribosyltransferase.

35. Symbol: **STH**; Name: **Microtubule-Associated Protein Tau (MAPT) Intronic Transcript**

The protein encoded by this gene is involved in positive regulation of mRNA splicing via spliceosome. It is located in nucleus and perinuclear region of cytoplasm. Diseases associated with STH include *Frontotemporal Dementia* and *Chromosome 17Q21.31 Duplication Syndrome*.

36. Symbol: **C10orf91** (**LINC02870**); Name: **Long Intergenic Non-Protein Coding RNA 2870**

No functions have been annotated to this non-coding RNA yet.

37. Symbol: **WNT9B**; Name: **Wnt Family Member 9B**

This gene is a member of the WNT gene family. The WNT gene family consists of structurally related genes that encode secreted signaling proteins. These proteins have been implicated in oncogenesis and in several developmental processes, including regulation of cell fate and patterning during embryogenesis. Study of its expression in the teratocarcinoma cell line NT2 suggests that it may be implicated in the early process of *neuronal* differentiation of NT2 cells induced by retinoic acid. This gene is clustered with WNT3, another family member, in the chromosome 17q21 region. It belongs to the canonical Wnt/beta-catenin signaling pathway. It activates a signaling cascade in the metanephric mesenchyme that induces tubulogenesis and plays a role in cranofacial development and is required for normal fusion of the palate during embryonic.

38. Symbol: **RUNX2**; Name: **RUNX Family Transcription Factor 2**

This gene is a member of the RUNX family of transcription factors and encodes a nuclear protein with an Runt DNA-binding domain. This protein is essential for osteoblastic differentiation and

skeletal morphogenesis and acts as a scaffold for nucleic acids and regulatory factors involved in skeletal gene expression. The protein can bind DNA both as a monomer or, with more affinity, as a subunit of a heterodimeric complex. Two regions of potential trinucleotide repeat expansions are present in the N-terminal region of the encoded protein, and these and other mutations in this gene have been associated with the bone development disorder cleidocranial dysplasia (CCD). Transcript variants that encode different protein isoforms result from the use of alternate promoters as well as alternate splicing. It is essential for the maturation of osteoblasts and both intramembranous and endochondral ossification. In osteoblasts, it supports transcription activation: synergizes with SPEN/MINT to enhance FGFR2-mediated activation of the osteocalcin FGF-responsive element (OCFRE).

39. Symbol: **C17orf53** (HROB); Name: **Homologous Recombination Factor With OB-Fold**

The protein encoded by this gene is predicted to enable single-stranded DNA binding activity and involved in DNA synthesis, DNA repair and interstrand cross-link repair. It functions by recruiting the MCM8-MCM9 helicase complex to sites of DNA damage to promote DNA repair synthesis.

40. Symbol: **EFCAB13**; Name: **EF-Hand Calcium Binding Domain 13**

EFCAB13 is a Protein Coding gene, and its annotations include calcium ion binding.

41. Symbol: **GJC1**; Name: **Gap Junction Protein Gamma 1**

This gene is a member of the connexin gene family. The encoded protein is a component of gap junctions, which are composed of arrays of intercellular channels that provide a route for the diffusion of low molecular weight materials from cell to cell. One gap junction consists of a cluster of closely packed pairs of transmembrane channels, the connexons, through which materials of low MW diffuse from one cell to a neighboring cell. Gene Ontology annotations related to this gene include ion channel activity and gap junction channel activity. They allow passive diffusion of molecules up to 1 kDa, including nutrients, metabolites (glucose), ions (K+, Ca2+) and second messengers (IP3, cAMP).

42. Symbol: **DBF4B**; Name: **DBF4 Zinc Finger B**

This gene encodes a regulator of the cell division cycle 7 homolog (S. cerevisiae) protein, a serine-threonine kinase which links cell cycle regulation to genome duplication. It is a regulatory subunit for CDC7 which activates its kinase activity thereby playing a central role in DNA replication and cell proliferation and is required for progression of S and M phases. The complex CDC7-DBF4B selectively phosphorylates MCM2 subunit at 'Ser-40' and then is involved in regulating the initiation of DNA replication during cell cycle.

43. Symbol: **HEXIM2**; Name: **HEXIM P-TEFb Complex Subunit 2**

This gene encodes a member of the HEXIM family of proteins. This protein is a component of the 7SK small nuclear ribonucleoprotein. This protein has been found to negatively regulate the kinase activity of the cyclin-dependent kinase P-TEFb, which phosphorylates multiple target proteins to promote transcriptional elongation. This gene is located approximately 7 kb downstream from related family member HEXIM1 on chromosome 17. It is a transcriptional regulator which functions as a general RNA polymerase II transcription inhibitor and is a core component of the 7SK RNP complex: in cooperation with 7SK snRNA sequesters P-TEFb in a large inactive 7SK snRNP complex preventing RNA polymerase II phosphorylation and subsequent transcriptional.

44. Symbol: **LRRC37A2**; Name: **leucine rich repeat containing 37 member A2**

The protein encoded by this gene is predicted to be an integral component of membrane. Diseases associated with LRRC37A2 include *Epilepsy*, *Progressive Myoclonic 6* and *Developmental And Epileptic Encephalopathy 96*.

45. Symbol: **DMGDH**; Name: **dimethylglycine dehydrogenase**

This gene encodes an enzyme involved in the catabolism of choline, catalyzing the oxidative demethylation of dimethylglycine to form sarcosine. The enzyme is found as a monomer in the mitochondrial matrix, and uses flavin adenine dinucleotide and folate as cofactors. Mutation in this gene causes *dimethylglycine dehydrogenase deficiency*, characterized by a fishlike body odor, chronic muscle fatigue, and elevated

levels of the muscle form of creatine kinase in serum.

GROUP 3

46. Symbol: **WNT3**; Name: **Wnt family member 3**

The WNT gene family consists of structurally related genes which encode secreted signaling proteins. These proteins have been implicated in oncogenesis and in several developmental processes, including regulation of cell fate and patterning during embryogenesis. Studies of the gene expression suggest that this gene may play a key role in some cases of human breast, rectal, lung, and gastric cancer through activation of the WNT-beta-catenin-TCF signaling pathway. The WNT3 protein functions in the canonical Wnt signaling pathway that results in activation of transcription factors of the TCF/LEF family. And it is required for normal gastrulation, formation of the primitive streak, and for the formation of the mesoderm during early embryogenesis.

47. Symbol: **C1QL1**; Name: **complement C1q like 1**

The protein encoded by this gene is predicted to enable signaling receptor binding activity, act upstream of or within maintenance of synapse structure, motor learning, and *neuron remodeling*. It is predicted to be located in several cellular components, including climbing fiber; presynapse; and synaptic cleft. It may also regulate the number of excitatory synapses that are formed on hippocampus neurons but has no effect on inhibitory synapses.

48. Symbol: **MAPT**; Name: **microtubule associated protein tau**

This gene encodes the microtubule-associated protein tau (MAPT) whose transcript undergoes complex, regulated alternative splicing, giving rise to several mRNA species. MAPT transcripts are differentially expressed in the nervous system, depending on stage of neuronal maturation and neuron type. This protein promotes microtubule assembly and stability, and might be involved in the establishment and maintenance of neuronal polarity. The C-terminus binds axonal microtubules while the N-terminus binds neural plasma membrane components, suggesting that tau functions as a linker protein between both. Axonal polarity is predetermined by TAU/MAPT localization (in the neuronal cell) in the domain of the cell body defined by the centrosome. The short isoforms allow plasticity of the cytoskeleton whereas the longer isoforms may preferentially play a role in its stabilization. MAPT gene mutations have been associated with several neurodegenerative disorders such as *Alzheimer's disease*, *Pick's disease*, *frontotemporal dementia*, *cortico-basal degeneration* and *progressive supranuclear palsy*.

49. Symbol: **CRHR1**; Name: **corticotropin releasing hormone receptor 1**

This gene encodes a G-protein coupled receptor that binds neuropeptides of the corticotropin releasing hormone family that are major regulators of the *hypothalamic-pituitary-adrenal pathway*. The encoded protein is essential for the activation of signal transduction pathways that regulate diverse physiological processes including stress, reproduction, immune response and obesity. This G-protein coupled receptor for CRH (corticotropin-releasing factor) and UCN (urocortin) has high affinity for CRH and UCN: Ligand binding causes a conformation change that triggers signaling via guanine nucleotide-binding proteins (G proteins) and down-stream effectors, such as adenylate cyclase. It promotes the activation of adenylate cyclase, leading to increased intracellular cAMP levels, inhibits the activity of the calcium channel CACNA1H. CRHR1 is required for normal embryonic development of the adrenal gland and for normal hormonal responses to stress.

50. Symbol: **ECE2**; Name: **Endothelin Converting Enzyme 2**

The enzym coded by this gene enables metalloendopeptidase activity. It is involved in peptide hormone processing, converts big endothelin-1 to endothelin-1. And it is also involved in the processing of various neuroendocrine peptides, including neurotensin, angiotensin I, substance P, proenkephalin-derived peptides, and prodynorphin-derived peptides.

51. Symbol: **CAMK2N2**; Name: **Calcium/Calmodulin Dependent Protein Kinase II Inhibitor 2**

This gene encodes a protein that is highly similar to the rat CaM-KII inhibitory protein, an inhibitor of calcium/calmodulin-dependent protein kinase II (CAMKII). CAMKII regulates numerous physiological

functions, including neuronal synaptic plasticity through the phosphorylation of alpha-amino-3-hydroxy-5-methyl-4-isoxazolepropionic acid-type glutamate (AMPA) receptors. Studies of the similar protein in rat suggest that this protein may function as a negative regulator of CaM-KII and may act to inhibit the phosphorylation of AMPA receptors. Gene Ontology (GO) annotations related to this gene include protein kinase binding and calcium-dependent protein kinase inhibitor activity. Diseases associated with CAMK2N2 include *Amyotrophic Lateral Sclerosis 21* and *Amyotrophic Lateral Sclerosis Type 6*.

52. Symbol: **VWA5B2**; Name: **Von Willebrand Factor A Domain Containing 5B2**

Variations in this gene have been associated with Mathemtaics ability. Somatic mutations in it have been reported in various cancers.

53. Symbol: **GFAP**; Name: **Glial fibrillary acidic protein**

This gene encodes one of the major intermediate filament proteins of mature astrocytes. It is used as a marker to distinguish astrocytes from other glial cells during development. Mutations in this gene cause Alexander disease, a rare disorder of astrocytes in the central nervous system. GFAP, a class-III intermediate filament, is a cell-specific marker that distinguishes astrocytes from other glial cells during the development of the central nervous system. Defects in GFAP are a cause of *Alexander disease* (ALEXD), which is a rare disorder of the central nervous system.

54. Symbol: **NKX6-2**; Name: **NK6 homeobox 2**

The protein encoded by this gene enables sequence-specific double-stranded DNA binding activity and is Predicted to be involved in cell differentiation, regulation of myelination and transcription. It acts upstream of or within several processes, including negative regulation of transcription by RNA polymerase II, neurogenesis; and neuromuscular process controlling balance. Diseases associated with mutations in this gene include *Spastic Ataxia 8*.

Table S6. Harmonized instrumental SNPs ($p<5x10^{-8}$) for the BAG to SCZ MR.

SNP	CHR	POS	A1	A2	A1_exp_frq	Beta_exp	SE_exp	N_exp	P_exp	A1_out_frq	Beta_out	SE_out	N_out	P_out
rs10137195	14	88444882	C	A	0.36	-0.16	0.0257	28104	1.42e-10	0.44	0.0056	0.0082	73173	0.49
rs2239923	17	43176804	T	C	0.29	-0.15	0.0269	28104	2.18e-8	0.28	0.0251	0.0086	73173	3.33e-3
rs2242189	4	38691024	C	T	0.38	0.21	0.0257	28104	1.24e-16	0.33	-0.0032	0.0084	73173	0.70
rs2790102	6	45432214	A	G	0.35	-0.15	0.0260	28104	8.92e-9	0.32	-0.0063	0.0084	73173	0.45
rs4880424	10	134584577	T	C	0.22	0.16	0.0296	28104	3.69e-8	0.20	-0.0121	0.0098	73173	0.22
rs62063276	17	44036408	G	T	0.21	0.28	0.0293	28104	2.72e-22	0.20	-0.0588	0.0108	60448	5.92e-8
rs7209501	17	43503294	C	A	0.18	0.29	0.0315	28104	5.33e-20	0.16	-0.0482	0.0114	64002	2.50e-5
rs7461069	8	124669029	A	G	0.21	-0.17	0.0309	28104	1.57e-8	0.18	-0.0149	0.01	73173	0.14

Instrumental SNPs select for the BAG to SCZ classical Mendelian randomization analysis. CHR: chromosome; POS: hg19 genomic position; A1: effective allele; A2: the other allele; A1_exp_frq: A1 frequency in the exposure dataset; Beta_exp, effect of A1 for the exposure; SE_exp; standard error for Beta_exp; N_exp: sample size for exposure; P_exp, association p value for exposure; A1_out_frq: A1 frequency in the outcome dataset; Beta_out: effect size of A1 on outcome; SE_out: standard error of Beta_out; N_out: sample size for outcome; P_out: association p value for outcome.

Table S7. Harmonized instrumental SNPs ($p<5x10^{-8}$) for the BAG to BIP MR.

SNP	CHR	POS	A1	A2	A1_exp_frq	Beta_exp	SE_exp	N_exp	P_exp	A1_out_frq	Beta_out	SE_out	N_out	P_out
rs10137195	14	88444882	C	A	0.36	-0.16	0.0257	28104	1.42e-10	0.39	-0.0151	0.0098	50981	0.13
rs2239923	17	43176804	T	C	0.29	-0.15	0.0269	28104	2.18e-8	0.28	-0.0333	0.0104	50981	1.3e-3
rs2242189	4	38691024	C	T	0.38	0.21	0.0257	28104	1.24e-16	0.34	-0.0021	0.0098	50981	0.83
rs2790102	6	45432214	A	G	0.35	-0.15	0.0260	28104	8.92e-9	0.35	0.0017	0.0098	50981	0.87
rs4880424	10	134584577	T	C	0.22	0.16	0.0296	28104	3.69e-8	0.21	-0.0045	0.0115	50981	0.69
rs62063276	17	44036408	G	T	0.21	0.28	0.0293	28104	2.72e-22	0.19	0.0215	0.0116	50981	0.06
rs7209501	17	43503294	C	A	0.18	0.29	0.0315	28104	5.34e-20	0.16	0.0218	0.0125	50981	0.08
rs7461069	8	124669029	A	G	0.21	-0.17	0.0309	28104	1.57e-8	0.20	0.0134	0.0118	50981	0.26

Instrumental SNPs select for the BAG to BIP classical Mendelian randomization analysis. CHR: chromosome; POS: hg19 genomic position; A1: effective allele; A2: the other allele; A1_exp_frq: A1 frequency in the exposure dataset; Beta_exp, effect of A1 for the exposure; SE_exp; standard error for Beta_exp; N_exp: sample size for exposure; P_exp, association p value for exposure; A1_out_frq: A1 frequency in the outcome dataset; Beta_out: effect size of A1 on outcome; SE_out: standard error of Beta_out; N_out: sample size for outcome; P_out: association p value for outcome.

Table S8. Harmonized instrumental SNPs ($p<5x10^{-8}$) for the BAG to MDD MR.

SNP	CHR	POS	A1	A2	A1_exp_frq	Beta_exp	SE_exp	N_exp	P_exp	A1_out_frq	Beta_out	SE_out	N_out	P_out
rs10137195	14	88444882	C	A	0.36	-0.16	0.0257	28104	1.421e-10	0.3596	0.0051	0.0082	69115	0.53
rs11079732	17	44161581	T	C	0.45	-0.14	0.0247	28104	4.67e-9	0.4537	-0.0076	0.0092	52547	0.41
rs1879581	17	43545893	C	T	0.18	0.29	0.0315	28104	3.23e-20	0.1766	0.0214	0.0105	69115	0.04
rs2239923	17	43176804	T	C	0.29	-0.15	0.0269	28104	2.18e-08	0.284	0.002	0.0088	69115	0.82
rs2242189	4	38691024	C	T	0.38	0.21	0.0257	28104	1.24e-16	0.3681	-0.0115	0.0082	69115	0.16
rs2790102	6	45432214	A	G	0.35	-0.15	0.0260	28104	8.92e-9	0.3487	0.0041	0.0084	69115	0.62
rs4880424	10	134584577	T	C	0.22	0.16	0.0296	28104	3.69e-8	0.2144	-0.0175	0.0103	69115	0.09
rs7461069	8	124669029	A	G	0.21	-0.17	0.0309	28104	1.57e-8	0.1953	0.0008	0.0099	69115	0.93

Instrumental SNPs select for the BAG to MDD classical Mendelian randomization analysis. CHR: chromosome; POS: hg19 genomic position; A1: effective allele; A2: the other allele; A1_exp_frq: A1 frequency in the exposure dataset; Beta_exp, effect of A1 for the exposure; SE_exp; standard error for Beta_exp; N_exp: sample size for exposure; P_exp, association p value for exposure; A1_out_frq: A1 frequency in the outcome dataset; Beta_out: effect size of A1 on outcome; SE_out: standard error of Beta_out; N_out: sample size for outcome; P_out: association p value for outcome.

Table S9. Harmonized instrumental SNPs ($p<5x10^{-8}$) for the BAG to AD MR.

SNP	CHR	POS	A1	A2	A1_exp_frq	Beta_exp	SE_exp	N_exp	P_exp	A1_out_frq	Beta_out	SE_out	N_out	P_out
rs10137195	14	88444882	C	A	0.36	-0.1648	0.0257	28104	1.421e-10	0.37	0.0008	0.0022	429826.3	0.71
rs2239923	17	43176804	T	C	0.29	-0.1507	0.0269	28104	4.67e-9	0.28	-0.0032	0.0024	428474.56	0.19
rs2242189	4	38691024	C	T	0.38	0.2126	0.0257	28104	3.23e-20	0.34	-0.0025	0.0023	417305.82	0.27
rs2790102	6	45432214	A	G	0.35	-0.1493	0.0260	28104	2.18e-08	0.35	-0.0056	0.0023	428230.78	0.01
rs4880424	10	134584577	T	C	0.22	0.1632	0.0296	28104	1.24e-16	0.22	0.0003	0.0026	426375.66	0.92
rs62063276	17	44036408	G	T	0.21	0.2842	0.0293	28104	8.92e-9	0.21	-0.0054	0.0027	427814.95	0.04
rs7209501	17	43503294	C	A	0.18	0.2888	0.0315	28104	3.69e-8	0.17	-0.0029	0.0028	429246.56	0.30
rs7461069	8	124669029	A	G	0.21	-0.1745	0.0309	28104	1.57e-8	0.18	-0.0011	0.0028	429105.83	0.69

Instrumental SNPs select for the BAG to AD classical Mendelian randomization analysis. CHR: chromosome; POS: hg19 genomic position; A1: effective allele; A2: the other allele; A1_exp_frq: A1 frequency in the exposure dataset; Beta_exp, effect of A1 for the exposure; SE_exp; standard error for Beta_exp; N_exp: sample size for exposure; P_exp, association p value for exposure; A1_out_frq: A1 frequency in the outcome dataset; Beta_out: effect size of A1 on outcome; SE_out: standard error of Beta_out; N_out: sample size for outcome; P_out: association p value for outcome.

Table S10. Harmonized instrumental SNPs ($p<5x10^{-8}$) for the BAG to PD MR.

SNP	CHR	POS	A1	A2	A1_exp_frq	Beta_exp	SE_exp	N_exp	P_exp	A1_out_frq	Beta_out	SE_out	N_out	P_out
rs10137195	14	88444882	C	A	0.36	-0.1648	0.0257	28104	1.421e-10	0.37	0.0602	0.0174	62649	5e-4
rs2239923	17	43176804	T	C	0.29	-0.1507	0.0269	28104	4.67e-9	0.31	0.0446	0.0214	62649	0.04
rs2242189	4	38691024	C	T	0.38	0.2126	0.0257	28104	3.23e-20	0.36	0.0151	0.0233	49863	0.52
rs2790102	6	45432214	A	G	0.35	-0.1493	0.0260	28104	2.18e-08	0.34	-0.0111	0.0238	49863	0.64
rs4880424	10	134584577	T	C	0.22	0.1632	0.0296	28104	1.24e-16	0.22	0.0078	0.0274	49863	0.77
rs62063276	17	44036408	G	T	0.21	0.2842	0.0293	28104	8.92e-9	0.22	-0.2507	0.027	60613	1.46e-20
rs7209501	17	43503294	C	A	0.18	0.2888	0.0315	28104	3.69e-8	0.19	-0.2225	0.0274	60613	4.66e-16
rs7461069	8	124669029	A	G	0.21	-0.1745	0.0309	28104	1.57e-8	0.19	0.0252	0.0256	62649	0.33

Instrumental SNPs select for the BAG to PD classical Mendelian randomization analysis. CHR: chromosome; POS: hg19 genomic position; A1: effective allele; A2: the other allele; A1_exp_frq: A1 frequency in the exposure dataset; Beta_exp, effect of A1 for the exposure; SE_exp; standard error for Beta_exp; N_exp: sample size for exposure; P_exp, association p value for exposure; A1_out_frq: A1 frequency in the outcome dataset; Beta_out: effect size of A1 on outcome; SE_out: standard error of Beta_out; N_out: sample size for outcome; P_out: association p value for outcome.

Table S11. Harmonized instrumental SNPs ($p<5x10^{-8}$) for the SCZ to BAG MR.

SNP	CHR	POS	A1	A2	A1_exp_frq	Beta_exp	SE_exp	N_exp	P_exp	A1_out_frq	Beta_out	SE_out	N_out	P_out
rs10098869	8	4208761	T	C	0.85	-0.07	0.0108	73173	2.85e-10	0.86	-0.0265	0.0358	28104	0.4
7rs10127983	1	153923276	C	T	0.70	-0.05	0.0084	73173	3.11e-8	0.70	-0.0233	0.0271	28104	0.39
rs10167385	2	37238824	C	T	0.68	-0.05	0.0086	73173	7.94e-9	0.75	0.0046	0.0287	28104	0.87
rs10238960	7	70773271	C	T	0.32	-0.05	0.0084	73173	7.65e-9	0.30	-0.021	0.0270	28104	0.44
rs10243922	7	138599980	G	A	0.73	-0.05	0.0089	73173	1.02e-8	0.69	0.0496	0.0267	28104	0.06
rs1034326	6	30084446	A	G	0.88	0.12	0.0126	66018	1.92e-22	0.84	0.0664	0.0326	28104	0.04
rs10447226	5	139028810	G	T	0.75	-0.05	0.0092	73173	1.99e-8	0.72	-0.0208	0.0272	28104	0.45
rs10520313	4	176744539	G	A	0.92	-0.09	0.014	73173	8.73e-11	0.93	-0.0424	0.0477	28104	0.37
rs10736470	11	113418371	G	A	0.29	-0.07	0.009	73173	1.11e-14	0.36	0.0169	0.0256	28104	0.51
rs10774034	12	2330458	C	T	0.66	-0.08	0.0085	73173	7.10e-23	0.61	-0.054	0.0251	28104	0.03
rs10774545	12	110564659	A	C	0.28	0.06	0.0086	73173	6.98e-13	0.28	-0.0109	0.0274	28104	0.69
rs10861176	12	104631552	G	A	0.27	-0.05	0.0086	73173	5.23e-9	0.27	5,003-4	0.0280	28104	0.99
rs10861879	12	108609634	G	A	0.64	-0.05	0.0083	73173	1.23e-8	0.69	0.0397	0.0264	28104	0.13
rs10873538	14	104255569	T	G	0.66	-0.06	0.0082	73173	9.59e-15	0.66	0.0409	0.0258	28104	0.11
rs10894308	11	130891895	G	A	0.54	0.05	0.0079	73173	1.36e-9	0.55	-0.0263	0.0247	28104	0.29
rs10906984	15	83366296	A	C	0.39	0.05	0.0079	73173	4.84e-10	0.38	-0.0228	0.0255	28104	0.37
rs10985811	9	101070487	T	C	0.80	-0.05	0.0098	73173	2.53e-8	0.83	0.0114	0.0333	28104	0.73
rs11038906	11	46536442	G	A	0.85	-0.09	0.0108	73173	6.43e-16	0.83	-0.0047	0.0327	28104	0.89
rs11122119	1	6768856	C	A	0.65	-0.05	0.0081	73173	2.31e-8	0.68	-0.0419	0.0262	28104	0.11
rs11159633	14	84670453	T	G	0.37	-0.05	0.008	73173	1.89e-8	0.38	0.0589	0.0251	28104	0.02
rs11167584	5	152004727	A	C	0.78	0.05	0.0095	73173	5.47e-9	0.73	0.0045	0.0269	28104	0.87
rs11210203	1	73775513	A	G	0.36	0.06	0.0082	73173	2.31e-15	0.37	0.0146	0.0255	28104	0.57
rs1121296	1	72174197	T	C	0.59	0.05	0.008	73173	3.74e-9	0.63	-0.0245	0.0255	28104	0.34
rs112205523	19	50114350	C	T	0.71	0.06	0.0088	73173	1.82e-10	0.69	0.0613	0.0266	28104	0.02

rs1143702	1	44086831	C	T	0.33	0.07	0.0082	73173	2.76e-16	0.3296	-0.0012	0.0259	28104	0.9631
rs1157635	11	24404596	A	G	0.52	-0.04	0.0078	73173	2.00e-8	0.4917	0.05	0.0246	28104	0.04242
rs11696755	20	48105317	T	C	0.83	-0.07	0.0104	73173	2.99e-10	0.824	-0.0148	0.0318	28104	0.6422
rs11715134	3	50552866	G	A	0.89	-0.07	0.0123	73173	2.52e-28	0.9105	-0.0361	0.0418	28104	0.3878
rs11807834	1	230272624	G	A	0.76	-0.05	0.0093	73173	1.12e-8	0.7399	-0.0294	0.0281	28104	0.2962
rs11890137	2	198816662	G	A	0.36	0.06	0.0081	73173	7.34e-12	0.3253	-0.0883	0.0262	28104	7.61e-4
rs11899740	2	73631212	G	A	0.79	-0.06	0.0103	63155	9.94e-9	0.7783	-0.0209	0.0295	28104	0.479
rs1191551	14	30000405	T	G	0.18	0.06	0.0103	73173	4.72e-9	0.2229	-0.0089	0.0291	28104	0.7598
rs11956576	5	91001001	A	C	0.78	-0.05	0.0093	73173	1.00e-8	0.7968	-0.0334	0.0310	28104	0.2821
rs12126806	1	200963825	C	T	0.75	0.05	0.009	73173	2.89e-8	0.7062	0.0392	0.0267	28104	0.1434
rs12131135	1	200418613	G	T	0.55	0.04	0.0081	73173	3.68e-8	0.4924	-0.0274	0.0244	28104	0.2632
rs12244388	10	104640052	G	A	0.61	0.07	0.008	73173	1.59e-18	0.6684	4,00E-04	0.0279	28104	0.9886
rs12277680	11	134586708	A	G	0.51	-0.05	0.0081	73173.9	3.84e-11	0.5707	0.0132	0.0248	28104	0.5947
rs12301769	12	72231313	A	C	0.91	-0.08	0.014	72630.42	1.92e-9	0.90081	-0.0052	0.0418	28104	0.9011
rs12311848	12	124486851	A	G	0.70	-0.05	0.0087	73173.9	1.65e-8	0.6713	0.0486	0.0259	28104	0.06069
rs12614183	2	105005181	C	T	0.78	-0.05	0.0095	73173.9	4.17e-8	0.8232	-0.0128	0.0320	28104	0.6898
rs12652777	5	155775075	T	C	0.43	0.	0.0079	73173.9	1.06e-8	0.4916	0.0137	0.0246	28104	0.5781
rs12668848	7	2020995	G	A	0.56	0.08	0.0078	73173.9	3.21e-26	0.5709	0.0043	0.0247	28104	0.8618
rs12713008	2	48503561	G	A	0.55	0.04	0.0078	73173.9	3.05e-8	0.5428	0.001	0.0239	28104	0.9667
rs12883788	14	33303540	C	T	0.57	-0.05	0.0079	73173.9	8.43e-12	0.547	-0.0085	0.0244	28104	0.7281
rs12905223	15	85114268	T	C	0.75	0.067	0.009	73173.9	1.65e-10	0.7288	-0.0252	0.0273	28104	0.3569
rs12950148	17	12872802	A	G	0.83	-0.06	0.0105	73173.9	1.48e-09	0.8189	-0.0467	0.0316	28104	0.1406
rs12969453	18	52751708	A	G	0.56	0.05	0.0078	73173.9	3.53e-12	0.5989	0.0059	0.02509	28104	0.8141
rs13090130	3	161777035	G	A	0.62	0.05	0.0079	73173.9	9.92e-11	0.6343	-0.0143	0.02538	28104	0.5732
rs13107325	4	103188709	C	T	0.93	-0.16	0.0168	58439.19	2.9e-21	0.9263	0.0877	0.0482	28104	0.06889
rs13115045	4	143866615	G	A	0.78	0.05	0.0094	73173.9	4.36e-8	0.7626	0.0301	0.0288	28104	0.2976
rs13211901	6	27187612	G	A	0.84	0.11	0.011	71487.15	9.02e-26	0.798	-0.018	0.0299	28104	0.5476

rs13219424	6	128332084	C	T	0.67	0.05	0.0084	73173	4.52e-8	0.6986	-0.0287	0.0268	28104	0.285
rs13230189	7	137073259	C	T	0.668	0.07	0.0081	73173	1.04e-18	0.6427	0.0358	0.0258	28104	0.166
rs133293	22	42382797	G	A	0.45	-0.05	0.0081	73173	1.85e-11	0.5284	-0.0394	0.0246	28104	0.1106
rs1401988	12	103561799	C	T	0.67	0.058	0.0085	73173	4.63e-8	0.631	0.0145	0.0251	28104	0.564
rs1480380	6	32913246	C	T	0.92	0.15	0.0159	59322	4.75e-22	0.899	0.1107	0.0394	28104	5.07e-3
rs1553933	2	60509080	C	T	0.38	0.04	0.008	73173	3.89e-8	0.4104	-0.0162	0.0249	28104	0.5157
rs1600295	3	181234906	C	T	0.17	-0.07	0.0109	73173	1.29e-11	0.2058	-0.0061	0.0304	28104	0.8413
rs16825349	2	146425531	A	G	0.83	-0.07	0.0104	73173	1.32e-11	0.8161	0.0219	0.0314	28104	0.4856
rs169738	6	33537546	A	G	0.42	-0.05	0.0079	73173	4.39e-11	0.4003	-0.0234	0.0250	28104	0.3508
rs17194490	3	2547786	G	T	0.84	-0.08	0.0116	60303	1.85e-11	0.8454	-0.0316	0.0330	28104	0.3394
rs17693963	6	27710165	A	C	0.92	0.19	0.0151	64563	1.32e-37	0.8858	0.0283	0.0368	28104	0.4431
rs1784223	11	65380248	T	C	0.70	-0.05	0.0086	73173	2.43e-8	0.6536	0.0329	0.0257	28104	0.2017
rs1924378	13	38361744	T	G	0.48	0.05	0.0081	73173	9.31e-9	0.4139	-0.0011	0.0249	28104	0.9648
rs1939514	11	132572894	T	C	0.52	0.06	0.0077	73173	1.06e-12	0.4942	-0.0443	0.0246	28104	0.0721
rs209180	6	28793605	C	T	0.61	0.05	0.0083	73173	6.93e-10	0.6087	0.0618	0.0251	28104	0.01366
rs2095401	6	43124720	C	T	0.83	0.06	0.0106	73173	4.61e-8	0.8643	-0.0097	0.0361	28104	0.7884
rs2119242	10	21344773	G	A	0.83	-0.06	0.0106	73173	1.34e-8	0.8686	-0.012	0.0354	28104	0.7351
rs215483	4	23377121	G	A	0.69	-0.05	0.0084	73173	1.59e-9	0.6638	0.02	0.02620	28104	0.4453
rs2161203	5	88696876	T	C	0.89	0.08	0.0135	62066	2.49e-9	0.8772	0.0063	0.03664	28104	0.8635
rs216221	17	2155430	G	A	0.32	-0.05	0.0082	73173	8.65e-10	0.3494	-0.0409	0.0256	28104	0.1115
rs217291	6	84393942	T	C	0.57	0.05	0.0078	73173	5.47e-10	0.5632	-0.0072	0.02479	28104	0.7715
rs2190864	14	72416219	T	C	0.39	0.07	0.008	73173	1.12e-16	0.4004	-0.0316	0.0248	28104	0.2044
rs2224086	1	115309590	C	A	0.20	-0.06	0.0103	73173	2.09e-8	0.2276	-0.0215	0.02936	28104	0.464
rs2252074	7	104594253	T	G	0.59	-0.07	0.0078	73173	1.27e-14	0.6017	0.0252	0.0250	28104	0.314
rs2319280	1	150014017	A	C	0.15	-0.07	0.0118	61788	1.37e-8	0.1617	-0.0177	0.03266	28104	0.5879
rs2381411	9	36319928	T	C	0.57	-0.05	0.0079	73173.9	1.28e-8	0.6055	0.0055	0.0253	28104	0.8283
rs2455415	13	38860697	C	T	0.6	-0.05	0.0081	73173.9	3.431e-09	0.5697	0.0366	0.02490	28104	0.1417

rs246024	5	140333952	C	T	0.47	0.05	0.0077	73173.9	1.71e-09	0.4789	0.0513	0.02454	28104	0.03659
rs2470578	3	17330092	G	A	0.60	0.05	0.0085	73173.9	3.49e-08	0.5221	0.0114	0.0246	28104	0.644
rs2686386	12	121639657	C	T	0.21	0.05	0.0096	73173.9	1.26e-08	0.1837	0.0028	0.0320	28104	0.9303
rs2710323	3	52815905	T	C	0.53	0.07	0.0077	73173.9	5.92e-22	0.533	-0.0034	0.02483	28104	0.8911
rs2802535	1	98508258	C	T	0.17	-0.10	0.0105	73173.9	3.54e-20	0.1852	0.0628	0.0313	28104	0.04486
rs2815731	6	73155285	C	A	0.64	0.05	0.0081	73173.9	3.41e-10	0.6654	-0.0408	0.0259	28104	0.1153
rs281774	2	200814572	C	T	0.20	0.08	0.0096	73173.9	3.68e-17	0.1881	0.0772	0.0308	28104	0.01223
rs28669908	15	78910267	C	A	0.80	0.08	0.0098	73173.9	9.25e-15	0.7851	0.0296	0.02996	28104	0.3233
rs28758902	18	53408187	C	T	0.54	-0.05	0.0077	73173.9	7.69e-12	0.569	0.0239	0.02499	28104	0.339
rs28768122	12	123885974	T	C	0.26	0.08	0.009	73173.9	3.71e-18	0.2508	0.0511	0.02892	28104	0.07733
rs28865701	18	77620911	A	G	0.62	-0.06	0.0079	73173.9	4.7e-16	0.6344	-0.0274	0.02548	28104	0.2824
rs2909457	2	162845855	G	A	0.38	0.05	0.0083	73173.9	3.09e-8	0.4546	0.0599	0.02459	28104	0.01488
rs2910032	5	152540354	C	T	0.42	0.06	0.008	73173.9	4.52e-12	0.4826	0.0368	0.02456	28104	0.1341
rs2914983	2	198314568	A	G	0.35	0.06	0.0081	73173.9	1.1e-14	0.3244	-0.0706	0.0260	28104	6.78e-3
rs2968533	7	71787660	G	A	0.60	0.05	0.008	73173.9	4.593e-9	0.5627	-0.0411	0.02453	28104	0.09387
rs308697	3	161487491	C	A	0.60	0.056	0.0079	73173.9	3.35e-9	0.5715	-0.0306	0.02453	28104	0.2123
rs312477	3	53515136	G	A	0.746	0.06	0.0089	73173.9	1.37e-10	0.7758	-0.0624	0.02916	28104	0.03237
rs3129963	6	32380208	A	G	0.84	0.09	0.0113	70162.94	8.34e-15	0.8193	0.1026	0.0308	28104	8.97e-4
rs3131063	6	30763756	G	A	0.55	0.05	0.0082	69694.12	6.50e-9	0.5554	0.0617	0.0246	28104	0.01238
rs3134797	6	32186050	G	A	0.59	0.07	0.0083	70162.94	2.42e-11	0.5667	0.0499	0.02464	28104	0.0429
rs322128	19	11402416	C	T	0.78	-0.06	0.0095	73173.9	2.08e-09	0.8021	0.008	0.0309	28104	0.7962
rs337718	18	69774278	T	C	0.30	0.05	0.0084	73173.9	4.39e-09	0.2937	-0.035	0.02695	28104	0.1942
rs34829140	2	155904379	C	A	0.80	-0.05	0.0098	73173.9	4.04e-08	0.7834	0.0216	0.02947	28104	0.4637
rs35045093	7	127738471	A	C	0.826	0.06	0.0103	73173.9	3.36e-08	0.8248	-0.0379	0.03322	28104	0.254
rs35339313	2	145139727	C	T	0.61	-0.06	0.008	73173.9	1.31e-12	0.6544	0.0248	0.02558	28104	0.3324
rs353547	3	52268866	T	C	0.39	-0.06	0.0079	73173.9	5.17e-14	0.3695	-0.0028	0.02553	28104	0.9127
rs35531336	7	133438132	A	G	0.89	0.07	0.013	73173.9	1.46e-08	0.8765	-0.0251	0.0370	28104	0.4978

rs35734242	4	706700	T	C	0.57	-0.045	0.008	73173.9	3.927e-10	0.5683	9,00E-04	0.0251	28104	0.9714
rs35844949	2	58380167	G	A	0.44	-0.07	0.0078	73173.9	6.203e-21	0.4404	-0.0058	0.0249	28104	0.8162
rs36013966	8	89302448	G	A	0.78	-0.06	0.0093	73173.9	7.548e-10	0.7662	-0.0403	0.029	28104	0.1678
rs36109883	6	26133070	G	A	0.93	0.18	0.0174	58248.36	6.789e-24	0.91004	0.0362	0.0407	28104	0.3741
rs3732386	3	36871993	C	T	0.70	-0.06	0.0087	73173.9	1.464e-10	0.6418	0.0433	0.025	28104	0.09107
rs3739554	9	129940645	A	G	0.83	-0.06	0.0103	73173.9	2.257e-08	0.8385	-0.0034	0.033	28104	0.9194
rs3765971	1	8445360	C	T	0.28	0.05	0.0088	73173.9	1.975e-09	0.3382	-0.0131	0.025	28104	0.6132
rs3779870	8	65519166	A	G	0.37	-0.05	0.008	73173.9	7.63e-09	0.3731	0.0588	0.0255	28104	0.02144
rs3808581	8	26250047	G	A	0.79	-0.07	0.0097	73173.9	3.823e-12	0.8335	0.0564	0.033	28104	0.08788
rs3929747	10	104214267	C	T	0.75	0.06	0.0091	73173.9	3.127e-12	0.7905	0.0041	0.03071	28104	0.8938
rs39967	5	57744788	T	C	0.16	-0.06	0.0107	73173.9	1.869e-08	0.1771	-0.0376	0.03223	28104	0.2434
rs4144848	2	200001233	G	A	0.54	0.07	0.0079	73173.9	2.328e-16	0.5789	0.0307	0.0248	28104	0.2163
rs41533650	4	176869252	G	A	0.80	-0.07	0.0097	73173.9	8.691e-14	0.7906	-0.0196	0.0300	28104	0.5139
rs4283241	16	29988349	G	A	0.57	0.06	0.0078	73173.9	2.531e-14	0.541	-0.0886	0.0244	28104	2.91e-4
rs431982	5	106732144	T	C	0.24	0.05	0.0093	73173.9	1.558e-08	0.1817	0.0593	0.0322	28104	0.06609
rs4327046	16	9942377	G	A	0.73	-0.06	0.0088	73173.9	4.1e-10	0.7061	-0.0198	0.0274	28104	0.47
rs4510068	17	44184828	G	T	0.60	0.05	0.0082	73173.9	1.757e-09	0.608	-0.1634	0.0254	28104	1.28e-10
rs459391	21	22120508	T	C	0.19	0.06	0.01	73173.9	1.543e-08	0.1932	0.0408	0.0308	28104	0.1863
rs4712938	6	25449269	T	C	0.21	-0.06	0.0097	73173.9	4.446e-09	0.237	0.0031	0.02914	28104	0.9153
rs4793888	17	55737740	G	A	0.79	-0.06	0.0097	73173.9	3.634e-10	0.8478	-0.0307	0.03431	28104	0.371
rs4808955	19	19552413	T	C	0.66	-0.06	0.0081	73173.9	1.967e-13	0.646	0.0502	0.02578	28104	0.05159
rs4936215	11	133852684	A	G	0.81	0.08	0.0104	72178.92	1.857e-15	0.7972	0.006	0.0303	28104	0.8431
rs496108	5	49532628	C	T	0.45	0.05	0.0082	73173.9	8.617e-10	0.4659	0.0152	0.0247	28104	0.539
rs4975957	5	46265302	A	G	0.46	0.05	0.0084	72359.83	3.581e-09	0.4686	0.0162	0.02479	28104	0.5135
rs4990036	6	31323506	C	T	0.89	0.15	0.0135	64853.91	1.034e-27	0.8577	0.075	0.03397	28104	0.02726
rs500102	9	77358745	T	C	0.43	0.04	0.0079	73173.9	4.153e-08	0.3944	0.0151	0.0250	28104	0.5467
rs505061	9	22767164	C	A	0.51	-0.05	0.0077	73173.9	1.025e-10	0.4962	-0.0162	0.02430	28104	0.5051

rs55704727	3	117616431	C	T	0.90	0.07	0.013	72630.42	3.296e-08	0.8757	0.0246	0.0365	28104	0.5014
rs56086712	10	3800240	T	C	0.50	0.04	0.0081	73173.9	4.389e-08	0.4377	0.0199	0.0251	28104	0.4294
rs5757730	22	39967430	A	G	0.39	-0.05	0.0085	73173.9	1.185e-10	0.4986	0.0427	0.0245	28104	0.08162
rs578470	12	50463325	T	C	0.62	-0.05	0.0083	73173.9	2.327e-08	0.5828	-0.0189	0.0248	28104	0.4467
rs58946679	1	2372321	C	T	0.61	-0.05	0.0084	73173.9	1.721e-10	0.6475	0.0067	0.0262	28104	0.7985
rs6010045	22	51103091	T	C	0.32	-0.05	0.0085	72992.88	1.815e-08	0.3176	0.0192	0.0264	28104	0.4681
rs6065094	20	37453194	A	G	0.32	-0.06	0.0082	73173.9	1.409e-14	0.3203	0.0074	0.0259	28104	0.7751
rs61405217	4	170322312	C	T	0.42	0.05	0.0079	73173.9	5.387e-11	0.4588	0.0035	0.0247	28104	0.8876
rs61789528	1	97157335	G	A	0.81	-0.06	0.0098	73173.9	8.782e-09	0.8214	-0.0191	0.0325	28104	0.5573
rs61828917	1	173580303	C	T	0.85	0.07	0.011	73173.9	7.951e-10	0.8812	0.0594	0.0374	28104	0.1125
rs61896127	11	47099867	C	T	0.91	-0.10	0.0151	59339.13	1.238e-10	0.91695	0.0297	0.04353	28104	0.4951
rs61937595	12	57682956	C	T	0.92	0.12	0.0158	69922.48	1.317e-14	0.9149	0.0096	0.0436	28104	0.8259
rs62176172	2	185499482	C	T	0.64	0.05	0.0081	73173.9	5.03e-10	0.6814	0.0152	0.02636	28104	0.5642
rs62184960	2	172974789	C	T	0.88	0.07	0.0122	73173.9	1.082e-08	0.8459	0.0027	0.0337	28104	0.9363
rs634940	6	93077500	G	T	0.79	-0.06	0.0098	73173.9	2.884e-11	0.7453	-0.0659	0.0278	28104	0.018
rs6482437	10	18726326	A	C	0.09	-0.10	0.0135	73173.9	1.05e-14	0.103	0.0296	0.03876	28104	0.4451
rs6500602	16	4497451	T	C	0.30	0.05	0.0084	73173.9	4.769e-08	0.2975	0.012	0.02699	28104	0.6567
rs6504163	17	61545779	C	T	0.37	-0.05	0.0082	73173.9	1.869e-09	0.3626	0.0028	0.02545	28104	0.9124
rs6538539	12	95195293	G	T	0.47	0.05	0.0077	73173.9	5.634e-10	0.4501	0.001	0.0252	28104	0.9684
rs6588168	1	66324118	C	T	0.48	-0.059	0.0079	73173.9	5.941e-10	0.4433	-0.0096	0.02488	28104	0.6997
rs6670165	1	177280121	C	T	0.81	-0.07	0.0098	73173.9	1.078e-11	0.81	-0.0547	0.03119	28104	0.07949
rs668437	3	135941739	G	A	0.42	0.05	0.0081	73173.9	9.253e-10	0.4735	-0.0461	0.0247	28104	0.06267
rs6713590	2	193937515	C	T	0.47	0.04	0.0077	73173.9	2.694e-08	0.481	-0.0335	0.0245	28104	0.1721
rs6715366	2	2327295	G	A	0.77	-0.06	0.0094	73173.9	4.225e-09	0.7258	0.0401	0.02728	28104	0.1416
rs67627854	2	76246645	T	G	0.82	-0.06	0.0101	73173.9	2.202e-08	0.7936	0.0167	0.0303	28104	0.5821
rs6839635	4	103872854	C	A	0.50	-0.04	0.0079	73173.9	3.874e-08	0.5082	0.0606	0.0245	28104	0.01339
rs6848123	4	80203425	A	C	0.56	0.05	0.0081	73173.9	2.054e-09	0.4971	-0.0035	0.02454	28104	0.8866

rs6919146	6	165059966	T	G	0.31	-0.05	0.0085	73173.9	8.423e-09	0.3404	-0.0242	0.02601	28104	0.3522
rs6925079	6	64946311	T	C	0.64	-0.04	0.0081	73173.9	3.581e-08	0.6802	-0.0154	0.02621	28104	0.5569
rs6926151	6	96476028	A	G	0.94	0.10	0.0176	59292.61	3.327e-08	0.92955	0.0647	0.04989	28104	0.1947
rs6943762	7	86403263	T	C	0.87	0.10	0.0124	73173.9	6.296e-17	0.8791	-0.0507	0.03707	28104	0.1715
rs6969410	7	110069015	T	G	0.66	0.06	0.0083	73173.9	1.899e-11	0.6211	-8.00e-04	0.02492	28104	0.9744
rs6984242	8	60700469	G	A	0.40	0.05	0.0078	73173.9	1.5e-11	0.4009	0.0127	0.0249	28104	0.6103
rs7002992	8	103676605	T	C	0.67	0.05	0.0083	73173.9	4.483e-09	0.6297	0.0116	0.02540	28104	0.6479
rs7007361	8	111621265	C	T	0.84	-0.07	0.0104	73173.9	4.429e-11	0.8274	-0.0144	0.0323	28104	0.6564
rs708228	11	57585662	C	T	0.70	-0.05	0.0085	73173.9	2.062e-09	0.6864	-0.0129	0.02617	28104	0.6221
rs7112912	11	134246900	C	T	0.41	0.05	0.0084	73173.9	2.09e-10	0.4674	-0.0502	0.02473	28104	0.04241
rs7113068	11	124633529	T	C	0.86	0.08	0.0112	73173.9	4.605e-12	0.8367	0.0313	0.03369	28104	0.3529
rs715170	18	53795514	C	T	0.75	0.06	0.009	73173.9	7.4e-13	0.743	-0.0392	0.0277	28104	0.1584
rs7177338	15	91428636	G	A	0.42	-0.07	0.0082	73173.9	2.024e-19	0.4703	-8.00e-04	0.0243	28104	0.9738
rs7178152	15	89787055	T	G	0.53	0.05	0.0078	73173.9	2.628e-10	0.5362	-0.0302	0.0244	28104	0.2177
rs717947	4	33653757	C	T	0.87	-0.07	0.0116	73173.9	3.096e-09	0.90095	-0.0537	0.0430	28104	0.2121
rs7207904	17	1267857	G	A	0.61	0.05	0.0079	73173.9	1.645e-09	0.6194	0.0155	0.0252	28104	0.5394
rs7238071	18	77579812	A	G	0.69	-0.0	0.0084	73173.9	9.292e-14	0.7263	-0.0452	0.02797	28104	0.1062
rs72728416	1	97834691	A	G	0.73	-0.06	0.0087	73173.9	4.99e-12	0.7566	-0.0042	0.0285	28104	0.8829
rs72761691	9	134786548	A	C	0.86	-0.07	0.0116	73173.9	1.07e-08	0.8598	0.0053	0.03540	28104	0.881
rs72769124	1	239210058	C	A	0.91	-0.10	0.0143	73173.9	4.36e-13	0.90654	0.0167	0.04214	28104	0.6919
rs72974269	2	225454907	C	T	0.68	0.05	0.0083	73173.9	2.76e-10	0.695	-0.0494	0.02648	28104	0.06219
rs7312697	12	29933069	T	C	0.36	-0.05	0.0081	73173.9	4.85e-11	0.3745	-0.0139	0.0253	28104	0.5838
rs73229090	8	27442127	C	A	0.89	0.10	0.0142	58749.13	4.34e-13	0.8933	0.0578	0.03875	28104	0.1359
rs74695911	7	131541469	T	C	0.82	0.06	0.0103	73173.9	4.897e-08	0.784	0.0373	0.02998	28104	0.2135
rs7563610	2	37566376	A	G	0.58	0.05	0.0079	73173.9	4.48e-10	0.6052	-0.0307	0.02504	28104	0.2202
rs758749	19	57189718	C	T	0.86	-0.06	0.0112	73173.9	4.66e-8	0.8696	0.0116	0.03733	28104	0.756
rs7600146	2	22543347	G	T	0.63	0.05	0.0083	73173.9	8.311e-09	0.569	-0.013	0.02485	28104	0.601

rs7609876	3	176790116	T	C	0.72	-0.05	0.0089	73173.9	9.398e-9	0.7932	0.0313	0.03031	28104	0.3018
rs7632834	3	17886678	T	C	0.42	-0.05	0.0085	73173.9	2.091e-9	0.5062	-0.0099	0.02461	28104	0.6875
rs7647398	3	180733150	C	T	0.82	0.09	0.01	73173.9	2.207e-17	0.8063	0.0122	0.03067	28104	0.6908
rs7703010	5	137711716	C	A	0.37	0.05	0.0082	73173.9	8.278e-09	0.4127	-0.0028	0.0247	28104	0.9101
rs7703618	5	44914579	G	A	0.44	0.05	0.008	73173.9	3.408e-09	0.3793	-0.0051	0.02550	28104	0.8415
rs7706116	5	45742624	G	T	0.56	-0.06	0.0078	73173.9	6.786e-13	0.5612	-0.0115	0.02502	28104	0.6458
rs77154381	2	27982119	A	G	0.68	0.05	0.0086	73173.9	2.6e-08	0.7468	0.0184	0.02833	28104	0.5161
rs778371	2	233743109	A	G	0.746	-0.05	0.0089	73173.9	1.103e-16	0.7248	0.016	0.02743	28104	0.5597
rs7811417	7	21534152	T	C	0.35	0.05	0.0081	73173.9	2.166e-09	0.33	-8,00E-04	0.02681	28104	0.9762
rs7816998	8	38257506	G	A	0.77	0.06	0.0092	73173.9	3.105e-10	0.7789	0.019	0.02893	28104	0.5114
rs7915131	10	64418656	C	T	0.42	0.04	0.0078	73173.9	4.943e-08	0.4319	0.051	0.02469	28104	0.03894
rs79210963	7	24717969	T	C	0.90	-0.09	0.0129	73173.9	2.584e-11	0.8922	-0.0214	0.03880	28104	0.5813
rs7927176	11	123395864	A	G	0.66	-0.05	0.0081	73173.9	6.604e-11	0.6617	0.0139	0.02606	28104	0.5938
rs8055219	16	13753384	G	A	0.79	-0.07	0.0095	73173.9	1.565e-12	0.7785	-0.0525	0.0295	28104	0.07584
rs8104557	19	31030189	T	C	0.82	-0.06	0.011	61674.31	3.764e-08	0.8023	-0.0594	0.03067	28104	0.05282
rs8180995	8	143326237	A	G	0.53	0.06	0.0078	73173.9	2.19e-15	0.5216	0.0405	0.0246	28104	0.1005
rs832195	3	63864612	T	C	0.34	0.05	0.0083	73173.9	2.969e-09	0.2972	0.0593	0.0271	28104	0.02872
rs9257802	6	29343355	C	T	0.83	0.09	0.0107	70162.94	1.182e-15	0.8034	0.0493	0.0306	28104	0.108
rs9287971	2	174931752	G	A	0.60	-0.05	0.0083	73173.9	3.824e-08	0.671	0.0407	0.026	28104	0.131
rs9304548	18	27500959	C	A	0.26	0.06	0.0089	73173.9	1.895e-11	0.2623	0.0041	0.0278	28104	0.8829
rs9318638	13	79986153	T	C	0.43	-0.05	0.0079	73173.9	2.044e-09	0.477	0.0169	0.0245	28104	0.4914
rs9393741	6	26654189	A	G	0.82	0.09	0.0102	73173.9	5.897e-19	0.7878	-0.0024	0.029	28104	0.9351
rs9454727	6	70003389	A	G	0.77	0.05	0.0098	70865.66	1.93e-08	0.7358	0.0164	0.02774	28104	0.5544
rs9569820	13	58702746	G	T	0.84	-0.07	0.0109	73173.9	6.563e-10	0.8836	0.0254	0.03889	28104	0.5137
rs9597388	13	56928696	G	A	0.82	0.07	0.0101	73173.9	3.239e-11	0.8067	0.0044	0.03113	28104	0.8876
rs9623320	22	41478482	G	A	0.76	-0.06	0.0092	73173.9	2.371e-10	0.7122	-0.045	0.0270	28104	0.09665
rs967005	6	28210688	C	T	0.83	0.07	0.0102	73173.9	1.3e-11	0.8046	0.0183	0.03081	28104	0.5526

rs9803993	1	243672125	G	A	0.78	0.05	0.0093	73173.9	1.497e-08	0.7811	0.0242	0.02959	28104	0.4136
rs9831201	3	80913228	T	G	0.72	0.054	0.0087	73173.9	6.7e-10	0.6993	-0.0132	0.02657	28104	0.6194
rs9882532	3	16865845	T	C	0.6	-0.0	0.0087	73173.9	8.574e-10	0.6518	-0.0494	0.02587	28104	0.05623
rs9891739	17	19942177	C	T	0.54	-0.04	0.008	73173.9	2.181e-08	0.4702	-0.0815	0.02449	28104	0.0008751
rs9926296	16	89818089	A	G	0.44	0.05	0.0086	71743.28	2.374e-08	0.49	0.0386	0.02457	28104	0.1162
rs9975024	21	16439883	A	G	0.53	-0.05	0.008	73173.9	1.777e-09	0.5254	0.012	0.02445	28104	0.6237
rs999494	2	73157395	C	T	0.82	0.06	0.0101	73173.9	1.044e-08	0.7975	-0.0032	0.03070	28104	0.917

Instrumental SNPs select for the SCZ to BAG classical Mendelian randomization analysis. CHR: chromosome; POS: hg19 genomic position; A1: effective allele; A2: the other allele; A1_exp_frq: A1 frequency in the exposure dataset; Beta_exp, effect of A1 for the exposure; SE_exp; standard error for Beta_exp; N_exp: sample size for exposure; P_exp, association p value for exposure; A1_out_frq: A1 frequency in the outcome dataset; Beta_out: effect size of A1 on outcome; SE_out: standard error of Beta_out; N_out: sample size for outcome; P_out: association p value for outcome.

Table S12. Harmonized instrumental SNPs ($p<5x10^{-8}$) for the BIP to BAG MR.

SNP	CHR	POS	A1	A2	A1_exp_frq	Beta_exp	SE_exp	N_exp	P_exp	A1_out_frq	Beta_out	SE_out	N_out	P_out
rs10043984	5	137712121	C	T	0.76	-0.06	0.0108	50981.48	3.709e-08	0.7468	-0.0202	0.0277	28104	0.467
rs10994299	10	62076628	C	A	0.95	-0.13	0.0201	50862.93	9.587e-11	0.94999	0.0062	0.0577	28104	0.9145
rs113779084	7	11871787	G	A	0.70	-0.08	0.0102	50981.48	1.423e-13	0.7099	-0.0301	0.0273	28104	0.2716
rs11647445	16	9926966	T	G	0.67	-0.05	0.0098	50981.48	2.152e-08	0.6472	-0.0169	0.0257	28104	0.5109
rs11764361	7	105043229	A	G	0.67	0.06	0.0104	50981.48	3.471e-09	0.6565	-0.0357	0.0258	28104	0.1674
rs11856299	15	83534421	C	T	0.75	0.06	0.011	50981.48	1.528e-08	0.7649	-0.0026	0.0295	28104	0.9298
rs12289486	11	79092527	C	T	0.89	-0.08	0.0149	50981.48	3.297e-08	0.892	0.0176	0.0389	28104	0.6517
rs1235162	6	29537224	A	G	0.91	0.11	0.0164	49000.67	6.899e-12	0.8804	0.0822	0.03623	28104	0.02328
rs12575685	11	70517927	G	A	0.67	-0.07	0.0101	50981.48	1.243e-10	0.7012	-0.0301	0.02643	28104	0.2549
rs12668848	7	2020995	G	A	0.58	0.06	0.0095	50981.48	1.902e-09	0.5709	0.0043	0.0247	28104	0.8618
rs12672003	7	24647222	A	G	0.89	-0.09	0.0155	46163.71	2.718e-09	0.8924	-0.0139	0.03892	28104	0.721
rs13044225	20	60865815	A	G	0.56	-0.05	0.0095	50186.29	8.498e-09	0.5319	0.0124	0.0244	28104	0.6127
rs13195401	6	26463574	G	T	0.92	0.14	0.0175	50615.04	6.979e-15	0.90102	0.039	0.0389	28104	0.3163
rs1487445	6	98565211	C	T	0.51	-0.07	0.0093	50981.48	1.484e-15	0.5324	0.0098	0.02467	28104	0.6913
rs17183814	2	166152389	G	A	0.92	0.10	0.0185	50772.41	2.678e-08	0.92605	0.0694	0.0464	28104	0.1354
rs17202645	2	169399768	C	T	0.81	0.07	0.0121	50981.48	2.494e-08	0.8177	-0.0273	0.0319	28104	0.3923
rs174581	11	61606683	G	A	0.63	-0.07	0.0097	50981.48	4.944e-13	0.6495	-0.0509	0.0256	28104	0.04681
rs17526722	6	25918855	G	A	0.93	0.10	0.0181	50615.04	1.759e-08	0.91019	0.0409	0.04075	28104	0.3156
rs17693963	6	27710165	A	C	0.91	0.12	0.0161	50772.41	5.836e-14	0.8858	0.0283	0.036	28104	0.4431
rs1894401	15	91429042	G	A	0.46	-0.05	0.0096	50035.72	2.799e-08	0.4709	1,00E-04	0.01899	28104	0.9958
rs1998821	10	18751242	G	A	0.89	0.08	0.0152	50981.48	4.25e-08	0.8948	-0.0299	0.038	28104	0.4395
rs2019611	15	85122620	C	A	0.75	0.07	0.0108	50981.48	1.437e-10	0.7509	-0.0423	0.0282	28104	0.1338
rs2069772	4	123373133	T	C	0.73	-0.06	0.0103	50981.48	1.567e-08	0.7318	-0.0386	0.0275	28104	0.1616
rs2126180	1	61105668	G	A	0.54	-0.06	0.0094	50981.48	1.621e-09	0.5298	-3,00E-04	0.0232	28104	0.9897
rs2159100	12	2346393	C	T	0.67	-0.08	0.0098	50981.48	1.872e-15	0.6682	-0.04	0.02614	28104	0.126

rs237475	20	48049506	T	C	0.52	-0.05	0.0093	50981.48	5.171e-09	0.5201	0.0134	0.02457	28104	0.5856
rs2577831	3	52628056	C	A	0.51	0.07	0.0093	50981.48	3.834e-13	0.5291	-0.003	0.02414	28104	0.9011
rs2719164	2	194437889	A	G	0.57	0.05	0.0095	50981.48	4.847e-08	0.5763	-0.011	0.02488	28104	0.6585
rs28565152	5	7542911	G	A	0.767	-0.07	0.0112	48268.74	1.963e-09	0.7557	-0.0354	0.02874	28104	0.2181
rs2899059	15	42844361	C	T	0.927	-0.1	0.017	50981.48	1.243e-08	0.91467	0.0753	0.04554	28104	0.09826
rs2953928	8	34152492	G	A	0.937	-0.12	0.02	50615.04	6.249e-09	0.94541	-0.0462	0.05104	28104	0.3654
rs3094035	6	30363136	G	T	0.907	0 1	0.0153	49000.67	4.168e-10	0.8645	0.0588	0.03447	28104	0.0881
rs3115674	6	31799076	T	G	0.897	0.08	0.0154	48295.99	3.871e-08	0.8713	0.0882	0.03535	28104	0.01261
rs3130557	6	31094703	C	T	0.89	0.09	0.0147	50862.93	9.734e-11	0.8564	0.074	0.033624	28104	0.02775
rs35741362	6	27007687	T	C	0.89	0.10	0.0148	50862.93	1.366e-12	0.8638	0.0135	0.0347	28104	0.6978
rs35958438	15	38973793	G	A	0.77	0.0	0.0117	47077.45	3.83e-08	0.7742	0.0192	0.0293	28104	0.5131
rs3856207	1	163747095	A	G	0.45	0.05	0.0095	50186.29	1.156e-08	0.4603	-0.0053	0.0244	28104	0.8284
rs39824	5	169255522	G	A	0.33	-0.06	0.0099	50981.48	9.568e-09	0.343	0.0217	0.0258	28104	0.4018
rs4072458	3	36862680	T	C	0.46	-0.08	0.0094	50981.48	5.233e-17	0.4529	0.0167	0.0246	28104	0.4973
rs41315395	6	32201469	C	A	0.86	-0.07	0.0127	49209.73	1.476e-08	0.8502	0.0314	0.03501	28104	0.3698
rs4619651	2	97416153	G	A	0.67	0.07	0.0101	50981.48	4.776e-11	0.6917	-0.0239	0.02633	28104	0.3642
rs4672	11	64009879	G	A	0.92	-0.10	0.0172	50035.72	3.42e-09	0.92068	-0.1075	0.04518	28104	0.01735
rs4790841	17	1835482	C	T	0.85	-0.07	0.0132	50035.72	3.141e-08	0.8462	0.0367	0.03379	28104	0.2775
rs531490	11	66324360	T	G	0.46	0.05	0.0093	50981.48	6.016e-09	0.456	-0.0145	0.02441	28104	0.5526
rs5758064	22	41153879	T	C	0.52	0.05	0.0093	50981.48	2.011e-08	0.5221	0.029	0.02474	28104	0.2413
rs61554907	17	38220432	G	T	0.88	-0.09	0.0154	50035.72	1.636e-08	0.8947	-0.0842	0.0395	28104	0.03331
rs67712855	20	43682551	T	G	0.69	0.07	0.0103	50186.29	4.221e-11	0.7129	0.0039	0.02710	28104	0.8856
rs6806239	3	70488207	T	G	0.80	0.07	0.0119	50981.48	2.641e-08	0.8163	-0.0315	0.03145	28104	0.3166
rs6865469	5	78849505	G	T	0.72	-0.06	0.0103	50981.48	1.65e-08	0.7309	-0.0366	0.02798	28104	0.1909
rs6887473	5	80961069	G	A	0.74	0.06	0.0105	50981.48	8.814e-09	0.7178	-0.0204	0.0272	28104	0.4546
rs6954854	7	21492589	G	A	0.43	0.06	0.0094	50981.48	5.936e-10	0.426	-0.0075	0.0249	28104	0.7634
rs7461753	8	144981498	A	C	0.6	-0.06	0.0098	50981.48	5.24e-09	0.5842	-0.0182	0.02501	28104	0.4668

rs763193	6	166996127	A	G	0.51	-0.0	0.0094	50981.48	1.192e-08	0.5185	-0.0142	0.0248	28104	0.5676
rs7813581	8	9882652	G	T	0.83	-0.08	0.0119	50981.48	9.911e-11	0.8286	0.0275	0.0325	28104	0.3988
rs9393926	6	28682725	G	A	0.09	-0.11	0.0165	49000.67	7.501e-12	0.1176	-0.031	0.0367	28104	0.3987

Instrumental SNPs select for the BIP to BAG classical Mendelian randomization analysis. CHR: chromosome; POS: hg19 genomic position; A1: effective allele; A2: the other allele; A1_exp_frq: A1 frequency in the exposure dataset; Beta_exp, effect of A1 for the exposure; SE_exp; standard error for Beta_exp; N_exp: sample size for exposure; P_exp, association p value for exposure; A1_out_frq: A1 frequency in the outcome dataset; Beta_out: effect size of A1 on outcome; SE_out: standard error of Beta_out; N_out: sample size for outcome; P_out: association p value for outcome.

Table S13. Harmonized instrumental SNPs ($p<5x10^{-8}$) for the MDD to BAG MR.

SNP	CHR	POS	A1	A2	A1_exp_frq	Beta_exp	SE_exp	N_exp	P_exp	A1_out_frq	Beta_out	SE_out	N_out	P_out
rs12658032	5	103904226	A	G	0.36	0.05	0.0083	69115.85	1.181e-10	0.3497	0.0225	0.0257	28104	0.3804
rs1460943	1	72813129	T	C	0.61	0.04	0.0081	69115.85	4.27e-8	0.609	-0.0145	0.0249	28104	0.5611
rs148696809	6	28934352	T	C	0.90	0.08	0.0132	69115.85	4.355e-9	0.8902	0.0412	0.037	28104	0.2712
rs2799077	6	28234597	T	C	0.15	-0.07	0.0112	69115.85	2.864e-9	0.1637	-0.0213	0.0327	28104	0.5153
rs4593766	1	73773043	T	C	0.38	0.05	0.0081	69115.85	1.705e-8	0.3694	0.0147	0.0254	28104	0.563
rs58825580	6	26365679	T	G	0.88	0.07	0.012	69115.85	1.258e-8	0.8664	0.0422	0.0349	28104	0.2272
rs6934329	6	27158033	A	G	0.82	0.06	0.0104	69115.85	1.416e-9	0.8128	-0.008	0.0308	28104	0.7952
rs6940116	6	27708732	A	G	0.84	0.06	0.0107	69115.85	4.912e-8	0.8228	-0.0048	0.0316	28104	0.8795
rs8013655	14	42089908	T	C	0.47	0.04	0.0079	69115.85	2.672e-8	0.4774	0.0518	0.0247	28104	0.03578

Instrumental SNPs select for the MDD to BAG classical Mendelian randomization analysis. CHR: chromosome; POS: hg19 genomic position; A1: effective allele; A2: the other allele; A1_exp_frq: A1 frequency in the exposure dataset; Beta_exp, effect of A1 for the exposure; SE_exp; standard error for Beta_exp; N_exp: sample size for exposure; P_exp, association p value for exposure; A1_out_frq: A1 frequency in the outcome dataset; Beta_out: effect size of A1 on outcome; SE_out: standard error of Beta_out; N_out: sample size for outcome; P_out: association p value for outcome.

Table S14. Harmonized instrumental SNPs ($p<5x10^{-8}$) for the AD to BAG MR.

SNP	CHR	POS	A1	A2	A1_exp_frq	Beta_exp	SE_exp	N_exp	P_exp	A1_out_frq	Beta_out	SE_out	N_out	P_out
rs10412413	19	45327309	T	C	0.3	0.0526	0.0023	429332.77	6.97e-115	0.32	0.0203	0.0264	28104	0.44
rs10808026	7	143099133	A	C	0.202	-0.0161	0.0027	425571.98	2.82e-9	0.22	-0.0219	0.03	28104	0.47
rs11257240	10	11719074	G	T	0.3	0.0129	0.0023	423921.06	1.28e-8	0.36	-0.0021	0.0252	28104	0.93
rs113260531	17	5138980	A	G	0.13	0.02	0.0033	429194.03	7.91e-10	0.12	0.0018	0.0376	28104	0.96
rs11672748	19	45490192	G	A	0.34=	0.023	0.0023	427777.91	7.25e-24	0.32	-0.026	0.0263	28104	0.32
rs117618017	15	63569902	T	C	0.12=	0.018	0.0033	428753.23	3.44e-8	0.14	0.0015	0.0345	28104	0.97
rs12539172	7	100091795	T	C	0.31=	-0.0167	0.0023	429961.08	5.85e-13	0.32	-0.0605	0.0261	28104	2.06e-2
rs12590654	14	92938855	A	G	0.34	-0.0148	0.0023	420451.85	1.32e-10	0.34	-0.0367	0.0263	28104	0.16
rs1532278	8	27466315	T	C	0.38	-0.0201	0.0022	428741.91	2.07e-19	0.39	0.0092	0.0251	28104	0.714
rs2081545	11	59958380	A	C	0.3	-0.0179	0.0022	425881.39	1.11e-15	0.40	-0.0624	0.025	28104	1.23e-2
rs2647066	6	32571122	T	C	0.13	-0.0208	0.0032	428954.21	8.55e-11	0.18	0.0101	0.0314	28104	0.75
rs28394864	17	47450775	A	G	0.45	0.0123	0.0022	424326.36	1.68e-8	0.45	0.0749	0.0248	28104	2.50e-3
rs28469095	19	45655333	C	T	0.1	0.0466	0.0036	426787.79	1.07e-38	0.09	0.0769	0.0427	28104	7.17e-2
rs28834970	8	27195121	C	T	0.37	0.0153	0.0022	428248.62	8.49e-12	0.36	0.0151	0.0254	28104	0.55
rs3752231	19	1043638	T	C	0.25	0.0159	0.0025	424712.83	2.38e-10	0.25	0.0665	0.0284	28104	1.93e-2
rs3810140	19	45316807	T	C	0.07	-0.0286	0.0043	424090.94	4.54e-11	0.06	0.0105	0.0562	28104	0.85
rs412776	19	45379516	A	G	0.11	0.02834	0.0035	428640.06	8.10e-16	0.12	-0.023	0.0379	28104	0.54
rs4575098	1	161155392	A	G	0.23	0.01641	0.0026	427808.62	1.90e-10	0.23	0.0763	0.0291	28104	8.69e-3
rs4663105	2	127891427	C	A	0.43	0.0311	0.0022	418699.97	1.45e-44	0.42	-0.0275	0.0252	28104	0.28
rs4803750	19	45247627	G	A	0.06	-0.0491	0.0044	427688.71	1.61e-28	0.07	0.0258	0.0481	28104	0.59
rs59007384	19	45396665	T	G	0.2	0.1389	0.0027	427829.43	1,00e-200	0.21	0.103	0.0306	28104	7.57e-4
rs593742	15	59045774	G	A	0.31	-0.0138	0.0023	426926.64	3.73e-9	0.31	0.0281	0.0267	28104	0.29
rs6014724	20	54998544	G	A	0.1	-0.0229	0.0037	427344.83	5.38e-10	0.09	0.0096	0.0433	28104	0.82
rs6448453	4	11026028	A	G	0.26	0.0147	0.0025	429961.08	1.98e-9	0.26	0.032	0.0276	28104	0.25
rs679515	1	207750568	T	C	0.17	0.0254	0.0027	429226.74	6.83e-19	0.17	0.0781	0.032	28104	1.47e-2

rs74862042	19	45217784	C	A	0.161	0.0281	0.0029	429642.96	9.11e-22	0.15	0.0142	0.0342	28104	0.68
rs846881	19	45078553	C	A	0.21	-0.0174	0.0027	422334.58	9.89e-11	0.21	-0.0278	0.0301	28104	0.36
rs867611	11	85776544	G	A	0.32	-0.0204	0.0023	427263.57	1.48e-18	0.31	0.0186	0.0263	28104	0.48
rs9381563	6	47432637	C	T	0.36	0.0145	0.0023	422732.93	1.99e-10	0.37	0.0211	0.0257	28104	0.41
rs9653111	19	45592475	T	C	0.11	-0.0194	0.0034	427366.64	1.22e-8	0.12	0.0228	0.0364	28104	0.53

Instrumental SNPs select for the AD to BAG classical Mendelian randomization analysis. CHR: chromosome; POS: hg19 genomic position; A1: effective allele; A2: the other allele; A1_exp_frq: A1 frequency in the exposure dataset; Beta_exp, effect of A1 for the exposure; SE_exp; standard error for Beta_exp; N_exp: sample size for exposure; P_exp, association p value for exposure; A1_out_frq: A1 frequency in the outcome dataset; Beta_out: effect size of A1 on outcome; SE_out: standard error of Beta_out; N_out: sample size for outcome; P_out: association p value for outcome.

Table S15. Harmonized instrumental SNPs ($p<5x10^{-8}$) for the PD to BAG MR.

SNP	CHR	POS	A1	A2	A1_exp_frq	Beta_exp	SE_exp	N_exp	P_exp	A1_out_frq	Beta_out	SE_out	N_out	P_out
rs10513789	3	182760073	T	G	0.82	0.1596	0.0219	62649	3.185e-13	0.80	0.0217	0.0312	28104	0.4874
rs11942911	4	90601310	A	G	0.92	-0.1872	0.0331	62649	1.566e-08	0.92	0.0745	0.0474	28104	0.1162
rs12817488	12	123296294	A	G	0.46	0.1032	0.0178	62649	7.111e-09	0.46	0.0452	0.0248	28104	0.06877
rs1372518	4	90757294	A	C	0.21	-0.2334	0.0209	62649	5.354e-29	0.22	0.0511	0.0299	28104	0.08718
rs1474055	2	169110394	T	C	0.13	0.1763	0.0248	62649	1.144e-12	0.12	-0.0547	0.0385	28104	0.1559
rs329652	11	133769699	A	G	0.36	0.1082	0.0174	62649.	5.027e-10	0.35	-0.0014	0.0256	28104	0.9565
rs34311866	4	951947	T	C	0.80	-0.2272	0.0231	62649	7.974e-23	0.83	-0.0409	0.0324	28104	0.207
rs35118762	6	32560631	T	C	0.16	-0.2042	0.0312	60613	6.026e-11	0.18	0.0123	0.0312	28104	0.6943
rs356175	4	90630814	T	C	0.66	-0.1538	0.0181	62649	1.607e-17	0.68	-0.0033	0.0261	28104	0.8997
rs4566208	17	16010920	A	G	0.43	0.0957	0.0174	62649	3.884e-08	0.44	0.0318	0.0246	28104	0.1967
rs4588066	18	40672964	A	G	0.33	0.1046	0.0178	62649	4.453e-09	0.32	0.0147	0.0264	28104	0.5781
rs4631042	4	15712550	A	C	0.53	0.1213	0.0169	62649	6.64e-13	0.52	0.0055	0.0249	28104	0.8254
rs4774417	15	61993702	A	G	0.74	0.1052	0.0192	62649	4.626e-08	0.73	-0.0355	0.0282	28104	0.2081
rs56236914	17	43483551	T	C	0.19	-0.2147	0.0258	62649	8.739e-17	0.17	0.2877	0.0316	28104	9.633e-20
rs620490	8	16697579	T	G	0.72	0.1174	0.019	62649	6.456e-10	0.752	0.0072	0.0270	28104	0.79
rs7210219	17	44018519	T	C	0.78	0.2607	0.0275	60613	2.657e-21	0.79	-0.2681	0.0301	28104	5.582e-19
rs76339698	5	60372588	A	G	0.89	-0.1818	0.0263	62649	5.00e-12	0.90	0.0423	0.0422	28104	0.3171
rs76904798	12	40614434	T	C	0.15	0.1352	0.0235	62649	9.224e-09	0.15	-0.0563	0.0348	28104	0.1059
rs7695720	4	77183300	A	C	0.79	0.1255	0.0208	62649	1.528e-09	0.78	0.0567	0.0294	28104	0.05426
rs858290	7	23248036	A	G	0.61	0.1036	0.0177	62649	4.443e-09	0.60	0.0166	0.0251	28104	0.5095

Instrumental SNPs select for the PD to BAG classical Mendelian randomization analysis. CHR: chromosome; POS: hg19 genomic position; A1: effective allele; A2: the other allele; A1_exp_frq: A1 frequency in the exposure dataset; Beta_exp, effect of A1 for the exposure; SE_exp; standard error for Beta_exp; N_exp: sample size for exposure; P_exp, association p value for exposure; A1_out_frq: A1 frequency in the outcome dataset; Beta_out: effect size of A1 on outcome; SE_out: standard error of Beta_out; N_out: sample size for outcome; P_out: association p value for outcome.

Table S16. Statistical finemapping for associated loci.

Locus	Lead SNP	PP_1	PP_2	PPI_1	SNP2	PPI_2
Chr3:183892867-183975709	rs73185796	0.51	0.46	0.7	rs11402585	0.07
Chr4:38591172-38779512	rs13132853	0.74	0.26	0.16	rs11727467	0.11
Chr5:78388694-78451813	rs79107704	0.75	0.25	0.48	rs185486757	0.03
Chr6:45407654-45511945	rs2790102	0.84	0.16	0.05	rs1934327	0.04
Chr8:124661974-124682971	rs7461069	0.82	0.18	0.1	rs13340533	0.09
Chr10:134544247-134597265	rs4880424	0.85	0.15	0.08	rs12767391	0.04
Chr14: 88391116-88556525	rs17203398	0.5	0.5	0.04	rs10137195	0.04
Chr17: 43101281-44863413	rs2106786	0.85	0.15	0.01	rs62062797	0.01

PP_1: posterior probability for the causal structural assuming only one causal variant in the locus. PP_2: posterior probability for the causal structural assuming only two causal variants. PPI_1: posterior probability of inclusion to the one-causal variant structure; PPI_2: posterior probability of inclusion for the ranked second SNP to the one-causal variant structure.

Table S17. eQTL statistics for rs73185796 from GTEx v8 portal.

Gencode Id	Gene Symbol	Variant Id	P-Value	NES	Tissue
ENSG00000145198.14	VWA5B2	chr3_184257921_G_T_b38	8.4e-6	-0.67	Brain - Cortex
ENSG00000161202.17	DVL3	chr3_184257921_G_T_b38	1.2e-5	-0.23	Thyroid
ENSG00000145198.14	VWA5B2	chr3_184257921_G_T_b38	1.9e-5	-0.57	Nerve - Tibial
ENSG00000145198.14	VWA5B2	chr3_184257921_G_T_b38	2.2e-5	-0.74	Brain - Frontal Cortex (BA9)
ENSG00000161202.17	DVL3	chr3_184257921_G_T_b38	2.9e-5	-0.23	Cells - Cultured fibroblasts
ENSG00000161202.17	DVL3	chr3_184257921_G_T_b38	3.3e-5	-0.23	Colon - Transverse
ENSG00000161203.13	AP2M1	chr3_184257921_G_T_b38	5.1e-5	-0.15	Whole Blood
ENSG00000161204.11	ABCF3	chr3_184257921_G_T_b38	7.2e-5	0.21	Skin - Sun Exposed (Lower leg)

p-value, eQTL association p value; NES, normalized effect size.

Table S18. eQTL statistics for rs13132853 from GTEx v8 portal.

Gencode Id	Gene Symbol	Variant Id	P-Value	NES	Tissue
ENSG00000231160.9	KLF3-AS1	chr4_38678394_A_G_b38	9.1e-6	-0.17	Whole Blood
ENSG00000231160.9	KLF3-AS1	chr4_38678394_A_G_b38	1.4e-5	-0.15	Artery - Tibial
ENSG00000231160.9	KLF3-AS1	chr4_38678394_A_G_b38	3.2e-5	-0.36	Brain - Cerebellum
ENSG00000174125.7	TLR1	chr4_38678394_A_G_b38	3.5e-5	0.15	Esophagus - Mucosa
ENSG00000231160.9	KLF3-AS1	chr4_38678394_A_G_b38	1.4e-4	-0.16	Muscle - Skeletal

p-value, eQTL association p value; NES, normalized effect size.

Table S19. eQTL statistics for rs2790102 from GTEx v8 portal.

Gencode Id	Gene Symbol	Variant Id	P-Value	NES	Tissue
ENSG00000124813.20	RUNX2	chr6_45464477_G_A_b38	7.9e-12	-0.54	Brain - Putamen (basal ganglia)
ENSG00000124813.20	RUNX2	chr6_45464477_G_A_b38	6.2e-10	-0.39	Brain - Caudate (basal ganglia)
ENSG00000124813.20	RUNX2	chr6_45464477_G_A_b38	1.1e-6	-0.35	Brain - Hippocampus
ENSG00000124813.20	RUNX2	chr6_45464477_G_A_b38	7.4e-6	-0.29	Brain - Spinal cord (cervical c-1)

p-value, eQTL association p value; NES, normalized effect size.

Table S20. eQTL statistics for rs17203398 from GTEx v8 portal.

Gencode Id	Gene Symbol	Variant Id	P-Value	NES	Tissue
ENSG00000054983.16	GALC	chr14_87983503_G_C_b38	3.3e-32	0.40	Artery - Tibial
ENSG00000054983.16	GALC	chr14_87983503_G_C_b38	4.6e-16	0.29	Adipose - Subcutaneous
ENSG00000054983.16	GALC	chr14_87983503_G_C_b38	4.4e-14	0.30	Artery - Aorta
ENSG00000054983.16	GALC	chr14_87983503_G_C_b38	7.7e-12	0.25	Skin - Sun Exposed (Lower leg)
ENSG00000054983.16	GALC	chr14_87983503_G_C_b38	2.7e-10	0.22	Esophagus - Muscularis
ENSG00000054983.16	GALC	chr14_87983503_G_C_b38	4.1e-10	0.20	Cells - Cultured fibroblasts
ENSG00000054983.16	GALC	chr14_87983503_G_C_b38	7.1e-9	0.22	Adipose - Visceral (Omentum)
ENSG00000054983.16	GALC	chr14_87983503_G_C_b38	1.3e-8	0.18	Thyroid
ENSG00000054983.16	GALC	chr14_87983503_G_C_b38	1.4e-7	0.21	Breast - Mammary Tissue
ENSG00000054983.16	GALC	chr14_87983503_G_C_b38	3.0e-7	0.20	Skin - Not Sun Exposed (Suprapubic)
ENSG00000054983.16	GALC	chr14_87983503_G_C_b38	1.0e-6	0.16	Muscle - Skeletal
ENSG00000054983.16	GALC	chr14_87983503_G_C_b38	2.9e-6	0.25	Adrenal Gland
ENSG00000054983.16	GALC	chr14_87983503_G_C_b38	7.6e-6	-0.38	Liver
ENSG00000054983.16	GALC	chr14_87983503_G_C_b38	9.6e-6	0.19	Esophagus - Gastroesophageal Junction
ENSG00000054983.16	GALC	chr14_87983503_G_C_b38	2.1e-5	0.21	Heart - Left Ventricle
ENSG00000054983.16	GALC	chr14_87983503_G_C_b38	2.8e-5	0.17	Heart - Atrial Appendage
ENSG00000054983.16	GALC	chr14_87983503_G_C_b38	4.0e-5	0.18	Artery - Coronary

p-value, eQTL association p value; NES, normalized effect size.

Table S21. eQTL statistics for rs2106786 from GTEx v8 portal.

Gencode Id	Gene Symbol	P-Value	NES	Tissue
ENSG00000204650.14	LINC02210	2.5e-219	1.2	Skin - Sun Exposed (Lower leg)
ENSG00000204650.14	LINC02210	1.4e-207	1.3	Adipose - Subcutaneous
ENSG00000214425.7	LRRC37A4P	2.2e-193	-1.2	Whole Blood
ENSG00000204650.14	LINC02210	1.3e-190	1.2	Nerve - Tibial
ENSG00000204650.14	LINC02210	1.1e-182	1.3	Artery - Tibial
ENSG00000204650.14	LINC02210	2.3e-180	1.2	Skin - Not Sun Exposed (Suprapubic)
ENSG00000204650.14	LINC02210	6.4e-178	1.1	Adipose - Visceral (Omentum)
ENSG00000214425.7	LRRC37A4P	2.3e-172	-1.3	Thyroid
ENSG00000204650.14	LINC02210	3.9e-169	1.0	Thyroid
ENSG00000204650.14	LINC02210	7.9e-166	1.3	Cells - Cultured fibroblasts
ENSG00000204650.14	LINC02210	4.3e-164	1.2	Lung
ENSG00000214425.7	LRRC37A4P	5.8e-162	-1.2	Skin - Sun Exposed (Lower leg)
ENSG00000204650.14	LINC02210	1.7e-161	1.3	Esophagus - Muscularis
ENSG00000214401.4	KANSL1-AS1	6.2e-158	1.2	Muscle - Skeletal
ENSG00000262539.1	RP11-259G18.3	5.0e-154	1.3	Whole Blood
ENSG00000263503.1	MAPK8IP1P2	1.5e-153	1.2	Muscle - Skeletal
ENSG00000214425.7	LRRC37A4P	3.4e-151	-1.2	Lung
ENSG00000214401.4	KANSL1-AS1	1.3e-150	1.3	Whole Blood
ENSG00000214425.7	LRRC37A4P	7.3e-150	-1.2	Adipose - Subcutaneous
ENSG00000263503.1	MAPK8IP1P2	1.2e-145	1.2	Whole Blood
ENSG00000214425.7	LRRC37A4P	1.2e-144	-1.3	Nerve - Tibial
ENSG00000204650.14	LINC02210	1.8e-144	1.3	Artery - Aorta
ENSG00000214401.4	KANSL1-AS1	9.6e-144	1.2	Artery - Tibial
ENSG00000214425.7	LRRC37A4P	8.8e-141	-1.2	Skin - Not Sun Exposed (Suprapubic)
ENSG00000204650.14	LINC02210	1.9e-140	0.83	Muscle - Skeletal
ENSG00000262539.1	RP11-259G18.3	1.9e-138	1.3	Artery - Tibial
ENSG00000264070.1	DND1P1	1.3e-136	1.3	Muscle - Skeletal
ENSG00000214401.4	KANSL1-AS1	2.4e-134	1.3	Skin - Sun Exposed (Lower leg)
ENSG00000262539.1	RP11-259G18.3	1.5e-131	1.3	Skin - Sun Exposed (Lower leg)
ENSG00000262539.1	RP11-259G18.3	8.6e-130	1.2	Muscle - Skeletal
ENSG00000204650.14	LINC02210	3.8e-129	0.99	Breast - Mammary Tissue
ENSG00000262500.1	MAPK8IP1P1	1.9e-126	1.1	Whole Blood
ENSG00000262539.1	RP11-259G18.3	3.1e-126	1.3	Adipose - Subcutaneous
ENSG00000214401.4	KANSL1-AS1	1.3e-125	1.2	Thyroid
ENSG00000262539.1	RP11-259G18.3	4.6e-123	1.2	Cells - Cultured fibroblasts
ENSG00000214425.7	LRRC37A4P	2.7e-121	-1.3	Esophagus - Muscularis
ENSG00000214401.4	KANSL1-AS1	5.8e-121	1.2	Adipose - Subcutaneous
ENSG00000214401.4	KANSL1-AS1	8.6e-121	1.3	Skin - Not Sun Exposed (Suprapubic)
ENSG00000262500.1	MAPK8IP1P1	3.6e-119	1.1	Skin - Sun Exposed (Lower leg)
ENSG00000262539.1	RP11-259G18.3	3.7e-119	1.3	Thyroid

ENSG00000263503.1	MAPK8IP1P2	4.9e-119	1.2	Skin - Sun Exposed (Lower leg)
ENSG00000214425.7	LRRC37A4P	5.7e-119	-1.1	Artery - Tibial
ENSG00000238083.7	LRRC37A2	1.6e-118	1.2	Adipose - Subcutaneous
ENSG00000204650.14	LINC02210	2.7e-117	0.88	Colon - Transverse
ENSG00000186868.15	MAPT	3.7e-117	1.2	Testis
ENSG00000214425.7	LRRC37A4P	2.2e-116	-1.1	Cells - Cultured fibroblasts
ENSG00000264070.1	DND1P1	1.9e-115	1.3	Skin - Sun Exposed (Lower leg)
ENSG00000263503.1	MAPK8IP1P2	1.7e-114	1.2	Adipose - Subcutaneous
ENSG00000214425.7	LRRC37A4P	1.1e-113	-1.2	Adipose - Visceral (Omentum)
ENSG00000238083.7	LRRC37A2	1.2e-113	1.1	Thyroid
ENSG00000214425.7	LRRC37A4P	1.3e-113	-1.2	Colon - Transverse
ENSG00000214401.4	KANSL1-AS1	2.6e-113	1.2	Nerve - Tibial
ENSG00000264070.1	DND1P1	9.7e-113	1.3	Whole Blood
ENSG00000214401.4	KANSL1-AS1	1.8e-112	1.3	Adipose - Visceral (Omentum)
ENSG00000204650.14	LINC02210	1.9e-112	1.2	Colon - Sigmoid
ENSG00000214401.4	KANSL1-AS1	2.9e-112	1.2	Cells - Cultured fibroblasts
ENSG00000262539.1	RP11-259G18.3	1.2e-110	1.3	Skin - Not Sun Exposed (Suprapubic)
ENSG00000214401.4	KANSL1-AS1	1.9e-110	1.3	Lung
ENSG00000263503.1	MAPK8IP1P2	4.9e-110	1.2	Artery - Tibial
ENSG00000263503.1	MAPK8IP1P2	9.3e-110	1.2	Thyroid
ENSG00000263503.1	MAPK8IP1P2	2.3e-109	1.2	Cells - Cultured fibroblasts
ENSG00000263503.1	MAPK8IP1P2	4.9e-109	1.2	Skin - Not Sun Exposed (Suprapubic)
ENSG00000238083.7	LRRC37A2	5.9e-108	1.2	Artery - Tibial
ENSG00000204650.14	LINC02210	1.2e-107	0.58	Whole Blood
ENSG00000204650.14	LINC02210	8.9e-107	1.1	Heart - Atrial Appendage
ENSG00000262500.1	MAPK8IP1P1	2.2e-106	1.2	Skin - Not Sun Exposed (Suprapubic)
ENSG00000214401.4	KANSL1-AS1	4.4e-104	1.2	Esophagus - Muscularis
ENSG00000280022.1	RP11-707O23.1	9.1e-104	1.1	Muscle - Skeletal
ENSG00000262539.1	RP11-259G18.3	1.1e-103	1.2	Esophagus - Mucosa
ENSG00000262539.1	RP11-259G18.3	1.8e-103	1.3	Nerve - Tibial
ENSG00000204650.14	LINC02210	6.5e-102	1.2	Esophagus - Gastroesophageal Junction
ENSG00000262500.1	MAPK8IP1P1	9.7e-102	1.1	Thyroid
ENSG00000264070.1	DND1P1	1.8e-101	1.3	Artery - Tibial
ENSG00000264070.1	DND1P1	1.3e-100	1.3	Adipose - Subcutaneous
ENSG00000262539.1	RP11-259G18.3	5.6e-99	1.3	Adipose - Visceral (Omentum)
ENSG00000264070.1	DND1P1	4.3e-98	1.2	Thyroid
ENSG00000263503.1	MAPK8IP1P2	8.1e-97	1.2	Adipose - Visceral (Omentum)
ENSG00000214401.4	KANSL1-AS1	3.6e-96	1.2	Esophagus - Mucosa
ENSG00000263503.1	MAPK8IP1P2	4.0e-96	1.2	Nerve - Tibial
ENSG00000238083.7	LRRC37A2	7.3e-96	1.2	Nerve - Tibial
ENSG00000264070.1	DND1P1	8.7e-96	1.3	Skin - Not Sun Exposed (Suprapubic)
ENSG00000263503.1	MAPK8IP1P2	6.1e-95	1.2	Esophagus - Mucosa

ENSG00000214425.7	LRRC37A4P	5.1e-94	-1.1	Breast - Mammary Tissue
ENSG00000214425.7	LRRC37A4P	3.0e-93	-1.2	Artery - Aorta
ENSG00000204650.14	LINC02210	4.4e-93	0.59	Esophagus - Mucosa
ENSG00000262539.1	RP11-259G18.3	6.7e-93	1.2	Lung
ENSG00000262500.1	MAPK8IP1P1	1.7e-92	1.1	Lung
ENSG00000262539.1	RP11-259G18.3	1.4e-91	1.2	Esophagus - Muscularis
ENSG00000238083.7	LRRC37A2	6.8e-91	1.1	Esophagus - Muscularis
ENSG00000264070.1	DND1P1	3.5e-90	1.2	Nerve - Tibial
ENSG00000263503.1	MAPK8IP1P2	2.1e-89	1.2	Lung
ENSG00000204650.14	LINC02210	8.7e-89	1.1	Heart - Left Ventricle
ENSG00000214425.7	LRRC37A4P	2.5e-87	-1.3	Esophagus - Gastroesophageal Junction
ENSG00000214401.4	KANSL1-AS1	2.6e-86	1.2	Artery - Aorta
ENSG00000204650.14	LINC02210	3.3e-86	1.1	Stomach
ENSG00000214425.7	LRRC37A4P	6.7e-86	-1.0	Esophagus - Mucosa
ENSG00000280022.1	RP11-707O23.1	8.4e-86	1.2	Adipose - Subcutaneous
ENSG00000214425.7	LRRC37A4P	1.0e-85	-1.2	Colon - Sigmoid
ENSG00000238083.7	LRRC37A2	1.4e-85	0.99	Adipose - Visceral (Omentum)
ENSG00000264070.1	DND1P1	1.4e-85	1.4	Adipose - Visceral (Omentum)
ENSG00000263503.1	MAPK8IP1P2	2.0e-85	0.97	Testis
ENSG00000264070.1	DND1P1	2.1e-85	1.3	Lung
ENSG00000263503.1	MAPK8IP1P2	2.7e-85	1.2	Esophagus - Muscularis
ENSG00000280022.1	RP11-707O23.1	2.2e-84	1.2	Artery - Tibial
ENSG00000264070.1	DND1P1	5.2e-84	1.2	Cells - Cultured fibroblasts
ENSG00000214401.4	KANSL1-AS1	6.3e-84	1.2	Breast - Mammary Tissue
ENSG00000204650.14	LINC02210	6.4e-83	1.2	Pancreas
ENSG00000214425.7	LRRC37A4P	9.4e-83	-1.2	Heart - Atrial Appendage
ENSG00000264070.1	DND1P1	1.2e-82	1.3	Breast - Mammary Tissue
ENSG00000238083.7	LRRC37A2	2.3e-82	0.95	Lung
ENSG00000214401.4	KANSL1-AS1	8.1e-82	1.2	Esophagus - Gastroesophageal Junction
ENSG00000238083.7	LRRC37A2	1.4e-81	0.98	Skin - Sun Exposed (Lower leg)
ENSG00000262500.1	MAPK8IP1P1	3.2e-81	1.1	Adipose - Visceral (Omentum)
ENSG00000214401.4	KANSL1-AS1	1.3e-80	1.2	Heart - Left Ventricle
ENSG00000214401.4	KANSL1-AS1	5.1e-80	1.2	Colon - Transverse
ENSG00000262539.1	RP11-259G18.3	7.9e-80	1.3	Breast - Mammary Tissue
ENSG00000262539.1	RP11-259G18.3	1.6e-79	1.2	Artery - Aorta
ENSG00000264070.1	DND1P1	8.4e-79	1.2	Esophagus - Muscularis
ENSG00000262539.1	RP11-259G18.3	9.5e-79	1.2	Heart - Left Ventricle
ENSG00000263503.1	MAPK8IP1P2	3.4e-76	1.2	Colon - Transverse
ENSG00000214425.7	LRRC37A4P	6.3e-76	-1.3	Pituitary
ENSG00000262500.1	MAPK8IP1P1	1.3e-75	0.98	Esophagus - Mucosa
ENSG00000214401.4	KANSL1-AS1	1.7e-75	1.2	Heart - Atrial Appendage
ENSG00000214425.7	LRRC37A4P	1.9e-75	-0.95	Muscle - Skeletal

ENSG00000238083.7	LRRC37A2	2.8e-75	1.1	Artery - Aorta
ENSG00000204650.14	LINC02210	6.5e-74	0.72	Testis
ENSG00000262539.1	RP11-259G18.3	2.1e-73	1.3	Heart - Atrial Appendage
ENSG00000263503.1	MAPK8IP1P2	2.9e-73	1.2	Heart - Left Ventricle
ENSG00000264070.1	DND1P1	3.0e-73	1.3	Colon - Transverse
ENSG00000280022.1	RP11-707O23.1	4.3e-73	1.1	Skin - Sun Exposed (Lower leg)
ENSG00000238083.7	LRRC37A2	5.2e-73	0.98	Muscle - Skeletal
ENSG00000186868.15	MAPT	7.5e-73	0.69	Esophagus - Mucosa
ENSG00000262539.1	RP11-259G18.3	8.4e-73	1.4	Testis
ENSG00000263503.1	MAPK8IP1P2	9.0e-73	1.2	Breast - Mammary Tissue
ENSG00000214401.4	KANSL1-AS1	1.3e-72	1.2	Colon - Sigmoid
ENSG00000214425.7	LRRC37A4P	3.6e-72	-1.3	Pancreas
ENSG00000263503.1	MAPK8IP1P2	8.4e-71	1.1	Artery - Aorta
ENSG00000280022.1	RP11-707O23.1	1.1e-70	0.96	Whole Blood
ENSG00000204650.14	LINC02210	3.0e-70	1.1	Prostate
ENSG00000262539.1	RP11-259G18.3	1.7e-69	1.3	Colon - Sigmoid
ENSG00000204650.14	LINC02210	6.5e-69	1.3	Adrenal Gland
ENSG00000214425.7	LRRC37A4P	1.2e-68	-1.1	Heart - Left Ventricle
ENSG00000238083.7	LRRC37A2	1.4e-68	1.1	Breast - Mammary Tissue
ENSG00000262539.1	RP11-259G18.3	1.4e-68	1.2	Colon - Transverse
ENSG00000280022.1	RP11-707O23.1	1.6e-68	1.1	Skin - Not Sun Exposed (Suprapubic)
ENSG00000214425.7	LRRC37A4P	1.9e-68	-1.0	Stomach
ENSG00000204650.14	LINC02210	7.7e-68	1.1	Pituitary
ENSG00000204650.14	LINC02210	1.3e-67	1.3	Spleen
ENSG00000214425.7	LRRC37A4P	6.6e-67	-1.1	Testis
ENSG00000280022.1	RP11-707O23.1	6.8e-67	1.1	Esophagus - Muscularis
ENSG00000238083.7	LRRC37A2	7.2e-67	0.95	Esophagus - Mucosa
ENSG00000262539.1	RP11-259G18.3	1.1e-66	1.2	Esophagus - Gastroesophageal Junction
ENSG00000214401.4	KANSL1-AS1	1.2e-66	1.3	Stomach
ENSG00000263503.1	MAPK8IP1P2	1.7e-66	1.2	Heart - Atrial Appendage
ENSG00000262500.1	MAPK8IP1P1	2.6e-66	1.1	Colon - Transverse
ENSG00000214401.4	KANSL1-AS1	4.8e-66	1.3	Testis
ENSG00000262500.1	MAPK8IP1P1	9.1e-66	1.3	Testis
ENSG00000262539.1	RP11-259G18.3	1.0e-65	1.3	Stomach
ENSG00000264070.1	DND1P1	1.2e-65	1.3	Heart - Left Ventricle
ENSG00000262500.1	MAPK8IP1P1	2.9e-65	0.99	Esophagus - Muscularis
ENSG00000238083.7	LRRC37A2	4.7e-65	0.91	Skin - Not Sun Exposed (Suprapubic)
ENSG00000204650.14	LINC02210	8.9e-65	1.3	Artery - Coronary
ENSG00000262500.1	MAPK8IP1P1	2.5e-64	1.2	Colon - Sigmoid
ENSG00000263503.1	MAPK8IP1P2	2.9e-63	1.2	Stomach
ENSG00000261575.2	RP11-259G18.1	7.1e-63	1.2	Testis
ENSG00000263503.1	MAPK8IP1P2	9.6e-63	1.2	Esophagus - Gastroesophageal Junction

ENSG00000264070.1	DND1P1	3.5e-62	1.3	Colon - Sigmoid
ENSG00000280022.1	RP11-707O23.1	4.4e-61	1.0	Nerve - Tibial
ENSG00000238083.7	LRRC37A2	1.2e-60	1.0	Esophagus - Gastroesophageal Junction
ENSG00000238083.7	LRRC37A2	1.9e-60	0.84	Whole Blood
ENSG00000263503.1	MAPK8IP1P2	1.3e-59	1.2	Colon - Sigmoid
ENSG00000262500.1	MAPK8IP1P1	1.2e-58	1.1	Breast - Mammary Tissue
ENSG00000264070.1	DND1P1	2.6e-58	1.2	Artery - Aorta
ENSG00000262500.1	MAPK8IP1P1	4.3e-57	1.1	Esophagus - Gastroesophageal Junction
ENSG00000214425.7	LRRC37A4P	7.4e-57	-1.2	Spleen
ENSG00000238083.7	LRRC37A2	1.1e-56	1.0	Heart - Left Ventricle
ENSG00000214425.7	LRRC37A4P	6.7e-56	-1.0	Brain - Nucleus accumbens (basal ganglia)
ENSG00000280022.1	RP11-707O23.1	1.7e-55	1.1	Adipose - Visceral (Omentum)
ENSG00000264070.1	DND1P1	3.8e-55	0.99	Esophagus - Mucosa
ENSG00000280022.1	RP11-707O23.1	6.7e-55	1.1	Esophagus - Gastroesophageal Junction
ENSG00000238083.7	LRRC37A2	8.6e-54	1.1	Colon - Sigmoid
ENSG00000238083.7	LRRC37A2	1.8e-53	1.1	Colon - Transverse
ENSG00000238083.7	LRRC37A2	1.9e-53	0.98	Heart - Atrial Appendage
ENSG00000262539.1	RP11-259G18.3	1.9e-53	1.2	Liver
ENSG00000204650.14	LINC02210	2.5e-52	1.2	Ovary
ENSG00000204650.14	LINC02210	3.3e-52	0.87	Small Intestine - Terminal Ileum
ENSG00000262500.1	MAPK8IP1P1	3.5e-52	0.91	Nerve - Tibial
ENSG00000262539.1	RP11-259G18.3	4.0e-52	1.3	Pancreas
ENSG00000238083.7	LRRC37A2	1.2e-51	1.0	Stomach
ENSG00000238083.7	LRRC37A2	1.4e-51	1.2	Brain - Cerebellum
ENSG00000214425.7	LRRC37A4P	1.9e-51	-1.0	Brain - Caudate (basal ganglia)
ENSG00000264070.1	DND1P1	2.0e-51	1.2	Stomach
ENSG00000214425.7	LRRC37A4P	2.1e-51	-1.2	Brain - Cortex
ENSG00000214425.7	LRRC37A4P	1.7e-50	-1.2	Artery - Coronary
ENSG00000264070.1	DND1P1	2.2e-50	1.2	Esophagus - Gastroesophageal Junction
ENSG00000214425.7	LRRC37A4P	2.2e-50	-1.0	Prostate
ENSG00000214401.4	KANSL1-AS1	2.5e-50	1.2	Pituitary
ENSG00000264070.1	DND1P1	2.9e-50	1.2	Heart - Atrial Appendage
ENSG00000238083.7	LRRC37A2	4.5e-50	1.2	Pituitary
ENSG00000264070.1	DND1P1	6.3e-50	1.2	Prostate
ENSG00000214401.4	KANSL1-AS1	7.0e-50	1.2	Liver
ENSG00000261575.2	RP11-259G18.1	7.1e-50	1.2	Brain - Cerebellum
ENSG00000214401.4	KANSL1-AS1	1.3e-49	1.1	Pancreas
ENSG00000280022.1	RP11-707O23.1	3.2e-49	1.2	Colon - Sigmoid
ENSG00000214401.4	KANSL1-AS1	4.6e-49	1.2	Brain - Cortex
ENSG00000280022.1	RP11-707O23.1	1.4e-47	1.0	Lung
ENSG00000185829.17	ARL17A	2.1e-47	0.88	Thyroid
ENSG00000262539.1	RP11-259G18.3	2.3e-47	1.3	Pituitary

ENSG00000280022.1	RP11-707O23.1	2.7e-47	1.1	Artery - Aorta
ENSG00000238083.7	LRRC37A2	3.6e-47	0.93	Cells - Cultured fibroblasts
ENSG00000263503.1	MAPK8IP1P2	4.4e-47	1.2	Liver
ENSG00000214401.4	KANSL1-AS1	1.4e-46	1.3	Adrenal Gland
ENSG00000280022.1	RP11-707O23.1	1.6e-46	1.1	Breast - Mammary Tissue
ENSG00000214425.7	LRRC37A4P	1.6e-46	-1.2	Brain - Frontal Cortex (BA9)
ENSG00000214425.7	LRRC37A4P	3.9e-46	-1.2	Cells - EBV-transformed lymphocytes
ENSG00000262539.1	RP11-259G18.3	4.2e-46	1.3	Prostate
ENSG00000262539.1	RP11-259G18.3	5.0e-46	1.4	Spleen
ENSG00000214425.7	LRRC37A4P	1.2e-45	-1.2	Brain - Hypothalamus
ENSG00000262539.1	RP11-259G18.3	1.3e-45	1.3	Adrenal Gland
ENSG00000214425.7	LRRC37A4P	1.5e-45	-1.1	Brain - Cerebellum
ENSG00000214425.7	LRRC37A4P	2.7e-45	-0.92	Small Intestine - Terminal Ileum
ENSG00000262500.1	MAPK8IP1P1	4.8e-45	1.2	Spleen
ENSG00000261575.2	RP11-259G18.1	5.5e-45	0.78	Skin - Sun Exposed (Lower leg)
ENSG00000262539.1	RP11-259G18.3	8.0e-45	1.2	Artery - Coronary
ENSG00000204652.6	RPS26P8	1.0e-44	0.98	Nerve - Tibial
ENSG00000238083.7	LRRC37A2	2.0e-44	1.2	Brain - Nucleus accumbens (basal ganglia)
ENSG00000214425.7	LRRC37A4P	3.2e-44	-1.2	Brain - Cerebellar Hemisphere
ENSG00000262539.1	RP11-259G18.3	4.6e-44	1.2	Brain - Cerebellum
ENSG00000214401.4	KANSL1-AS1	5.3e-44	1.2	Prostate
ENSG00000280022.1	RP11-707O23.1	6.2e-44	1.1	Colon - Transverse
ENSG00000176681.14	LRRC37A	9.4e-44	0.95	Nerve - Tibial
ENSG00000214401.4	KANSL1-AS1	1.1e-43	1.1	Brain - Nucleus accumbens (basal ganglia)
ENSG00000176681.14	LRRC37A	1.1e-43	0.87	Artery - Tibial
ENSG00000214425.7	LRRC37A4P	1.4e-43	-1.2	Brain - Hippocampus
ENSG00000214401.4	KANSL1-AS1	1.6e-43	1.2	Artery - Coronary
ENSG00000263503.1	MAPK8IP1P2	4.0e-43	1.2	Pituitary
ENSG00000214425.7	LRRC37A4P	4.4e-43	-1.2	Adrenal Gland
ENSG00000280022.1	RP11-707O23.1	5.6e-43	0.82	Cells - Cultured fibroblasts
ENSG00000238083.7	LRRC37A2	2.1e-42	1.3	Brain - Cerebellar Hemisphere
ENSG00000214401.4	KANSL1-AS1	2.3e-42	1.2	Brain - Cerebellum
ENSG00000238083.7	LRRC37A2	6.8e-42	1.2	Brain - Cortex
ENSG00000264070.1	DND1P1	8.8e-42	1.2	Pituitary
ENSG00000108379.9	WNT3	1.7e-41	0.66	Thyroid
ENSG00000264070.1	DND1P1	2.6e-41	1.2	Small Intestine - Terminal Ileum
ENSG00000263503.1	MAPK8IP1P2	3.0e-41	1.2	Prostate
ENSG00000214401.4	KANSL1-AS1	3.4e-41	1.2	Brain - Putamen (basal ganglia)
ENSG00000238083.7	LRRC37A2	6.6e-41	1.3	Brain - Hypothalamus
ENSG00000238083.7	LRRC37A2	7.5e-41	1.2	Brain - Caudate (basal ganglia)
ENSG00000261575.2	RP11-259G18.1	9.8e-41	1.2	Brain - Cerebellar Hemisphere
ENSG00000264070.1	DND1P1	1.1e-40	1.3	Liver

ENSG00000214401.4	KANSL1-AS1	1.1e-40	1.3	Small Intestine - Terminal Ileum
ENSG00000264070.1	DND1P1	1.4e-40	1.3	Adrenal Gland
ENSG00000280022.1	RP11-707O23.1	1.8e-40	1.1	Heart - Atrial Appendage
ENSG00000263503.1	MAPK8IP1P2	2.6e-40	1.3	Adrenal Gland
ENSG00000225190.10	PLEKHM1	5.5e-40	-1.0	Brain - Cerebellum
ENSG00000263503.1	MAPK8IP1P2	8.4e-40	1.2	Spleen
ENSG00000262539.1	RP11-259G18.3	1.0e-39	1.2	Brain - Nucleus accumbens (basal ganglia)
ENSG00000262539.1	RP11-259G18.3	1.5e-39	1.3	Brain - Caudate (basal ganglia)
ENSG00000262539.1	RP11-259G18.3	1.6e-39	1.2	Brain - Cortex
ENSG00000204650.14	LINC02210	2.3e-39	1.1	Uterus
ENSG00000262500.1	MAPK8IP1P1	2.6e-39	1.2	Prostate
ENSG00000262500.1	MAPK8IP1P1	2.7e-39	1.2	Pituitary
ENSG00000280022.1	RP11-707O23.1	6.6e-39	1.0	Heart - Left Ventricle
ENSG00000176681.14	LRRC37A	6.8e-39	0.76	Skin - Sun Exposed (Lower leg)
ENSG00000262539.1	RP11-259G18.3	9.8e-39	1.3	Small Intestine - Terminal Ileum
ENSG00000263503.1	MAPK8IP1P2	1.1e-38	1.3	Small Intestine - Terminal Ileum
ENSG00000214425.7	LRRC37A4P	1.8e-38	-1.2	Brain - Anterior cingulate cortex (BA24)
ENSG00000280022.1	RP11-707O23.1	3.7e-38	1.1	Pancreas
ENSG00000280022.1	RP11-707O23.1	4.6e-38	0.80	Thyroid
ENSG00000214401.4	KANSL1-AS1	7.2e-38	1.4	Minor Salivary Gland
ENSG00000214425.7	LRRC37A4P	1.2e-37	-1.1	Liver
ENSG00000263503.1	MAPK8IP1P2	1.3e-37	1.2	Artery - Coronary
ENSG00000214401.4	KANSL1-AS1	1.4e-37	1.2	Spleen
ENSG00000280022.1	RP11-707O23.1	2.4e-37	1.0	Stomach
ENSG00000280022.1	RP11-707O23.1	2.5e-37	1.2	Liver
ENSG00000204650.14	LINC02210	4.8e-37	0.69	Brain - Nucleus accumbens (basal ganglia)
ENSG00000280022.1	RP11-707O23.1	5.1e-37	1.0	Testis
ENSG00000204650.14	LINC02210	5.3e-37	0.89	Brain - Caudate (basal ganglia)
ENSG00000262500.1	MAPK8IP1P1	6.2e-37	1.2	Brain - Cerebellum
ENSG00000238083.7	LRRC37A2	6.9e-37	1.1	Prostate
ENSG00000214425.7	LRRC37A4P	9.4e-37	-1.4	Ovary
ENSG00000214401.4	KANSL1-AS1	1.1e-36	1.1	Brain - Caudate (basal ganglia)
ENSG00000238083.7	LRRC37A2	1.6e-36	1.0	Artery - Coronary
ENSG00000185829.17	ARL17A	1.8e-36	1.1	Brain - Cerebellum
ENSG00000263503.1	MAPK8IP1P2	1.9e-36	1.2	Brain - Cerebellum
ENSG00000264070.1	DND1P1	1.9e-36	1.3	Spleen
ENSG00000264070.1	DND1P1	8.6e-36	1.1	Pancreas
ENSG00000238083.7	LRRC37A2	1.2e-35	1.0	Pancreas
ENSG00000263503.1	MAPK8IP1P2	1.3e-35	1.0	Pancreas
ENSG00000238083.7	LRRC37A2	1.5e-35	1.2	Brain - Putamen (basal ganglia)
ENSG00000262539.1	RP11-259G18.3	3.4e-35	1.2	Brain - Putamen (basal ganglia)
ENSG00000159314.11	ARHGAP27	3.9e-35	0.39	Skin - Not Sun Exposed (Suprapubic)

ENSG00000238083.7	LRRC37A2	4.3e-35	1.2	Brain - Frontal Cortex (BA9)
ENSG00000238083.7	LRRC37A2	5.5e-35	1.1	Adrenal Gland
ENSG00000264070.1	DND1P1	6.2e-35	1.3	Artery - Coronary
ENSG00000261575.2	RP11-259G18.1	6.9e-35	0.73	Skin - Not Sun Exposed (Suprapubic)
ENSG00000263503.1	MAPK8IP1P2	1.5e-34	1.2	Brain - Cortex
ENSG00000204650.14	LINC02210	1.6e-34	1.3	Brain - Spinal cord (cervical c-1)
ENSG00000176681.14	LRRC37A	2.6e-34	0.80	Adipose - Subcutaneous
ENSG00000176681.14	LRRC37A	3.3e-34	0.74	Skin - Not Sun Exposed (Suprapubic)
ENSG00000228696.8	ARL17B	5.5e-34	1.1	Testis
ENSG00000262539.1	RP11-259G18.3	5.7e-34	1.3	Brain - Cerebellar Hemisphere
ENSG00000262500.1	MAPK8IP1P1	7.2e-34	1.1	Liver
ENSG00000262539.1	RP11-259G18.3	7.9e-34	1.3	Cells - EBV-transformed lymphocytes
ENSG00000262500.1	MAPK8IP1P1	8.8e-34	1.1	Small Intestine - Terminal Ileum
ENSG00000264070.1	DND1P1	9.1e-34	1.2	Brain - Cortex
ENSG00000238083.7	LRRC37A2	9.3e-34	1.2	Brain - Hippocampus
ENSG00000214401.4	KANSL1-AS1	2.1e-33	1.1	Brain - Cerebellar Hemisphere
ENSG00000214401.4	KANSL1-AS1	2.8e-33	1.2	Vagina
ENSG00000225190.10	PLEKHM1	3.9e-33	0.31	Muscle - Skeletal
ENSG00000159314.11	ARHGAP27	4.3e-33	0.40	Skin - Sun Exposed (Lower leg)
ENSG00000185829.17	ARL17A	8.2e-33	1.1	Brain - Cerebellar Hemisphere
ENSG00000214401.4	KANSL1-AS1	8.3e-33	1.1	Brain - Frontal Cortex (BA9)
ENSG00000186868.15	MAPT	3.4e-32	0.59	Lung
ENSG00000214425.7	LRRC37A4P	3.4e-32	-1.3	Brain - Spinal cord (cervical c-1)
ENSG00000262539.1	RP11-259G18.3	4.1e-32	1.4	Minor Salivary Gland
ENSG00000204650.14	LINC02210	6.3e-32	0.81	Brain - Hypothalamus
ENSG00000263503.1	MAPK8IP1P2	1.4e-31	1.2	Brain - Nucleus accumbens (basal ganglia)
ENSG00000262500.1	MAPK8IP1P1	1.6e-31	1.1	Brain - Cortex
ENSG00000214425.7	LRRC37A4P	1.9e-31	-1.1	Brain - Putamen (basal ganglia)
ENSG00000280022.1	RP11-707O23.1	7.8e-31	0.72	Esophagus - Mucosa
ENSG00000262881.1	RP11-669E14.4	1.6e-30	-0.62	Liver
ENSG00000280022.1	RP11-707O23.1	1.8e-30	1.1	Adrenal Gland
ENSG00000262500.1	MAPK8IP1P1	2.2e-30	1.2	Brain - Cerebellar Hemisphere
ENSG00000204650.14	LINC02210	7.1e-30	0.79	Brain - Hippocampus
ENSG00000214425.7	LRRC37A4P	1.1e-29	-1.1	Brain - Amygdala
ENSG00000238083.7	LRRC37A2	1.2e-29	1.2	Brain - Anterior cingulate cortex (BA24)
ENSG00000262500.1	MAPK8IP1P1	1.5e-29	1.1	Brain - Caudate (basal ganglia)
ENSG00000204650.14	LINC02210	2.0e-29	0.85	Brain - Putamen (basal ganglia)
ENSG00000238083.7	LRRC37A2	2.2e-29	1.1	Spleen
ENSG00000176681.14	LRRC37A	2.5e-29	0.75	Esophagus - Muscularis
ENSG00000262539.1	RP11-259G18.3	2.8e-29	1.1	Brain - Hypothalamus
ENSG00000232300.1	FAM215B	4.5e-29	0.72	Thyroid
ENSG00000204650.14	LINC02210	4.5e-29	0.69	Vagina

ENSG00000262539.1	RP11-259G18.3	5.4e-29	1.3	Uterus
ENSG00000204650.14	LINC02210	1.0e-28	0.75	Brain - Anterior cingulate cortex (BA24)
ENSG00000204650.14	LINC02210	1.0e-28	0.91	Brain - Amygdala
ENSG00000262539.1	RP11-259G18.3	1.0e-28	1.2	Brain - Frontal Cortex (BA9)
ENSG00000204650.14	LINC02210	1.3e-28	1.1	Minor Salivary Gland
ENSG00000214425.7	LRRC37A4P	1.4e-28	-1.3	Uterus
ENSG00000214401.4	KANSL1-AS1	1.4e-28	1.1	Brain - Hippocampus
ENSG00000264070.1	DND1P1	2.1e-28	1.2	Brain - Putamen (basal ganglia)
ENSG00000263503.1	MAPK8IP1P2	3.2e-28	1.1	Brain - Putamen (basal ganglia)
ENSG00000204650.14	LINC02210	4.6e-28	0.60	Brain - Cortex
ENSG00000263503.1	MAPK8IP1P2	5.3e-28	1.1	Cells - EBV-transformed lymphocytes
ENSG00000185829.17	ARL17A	5.5e-28	0.61	Muscle - Skeletal
ENSG00000280022.1	RP11-707O23.1	5.8e-28	1.1	Artery - Coronary
ENSG00000214425.7	LRRC37A4P	6.2e-28	-1.3	Brain - Substantia nigra
ENSG00000263503.1	MAPK8IP1P2	8.2e-28	1.2	Brain - Hypothalamus
ENSG00000262500.1	MAPK8IP1P1	8.3e-28	1.1	Brain - Nucleus accumbens (basal ganglia)
ENSG00000185829.17	ARL17A	8.9e-28	1.1	Brain - Cortex
ENSG00000263503.1	MAPK8IP1P2	9.2e-28	1.3	Minor Salivary Gland
ENSG00000263503.1	MAPK8IP1P2	1.1e-27	1.2	Brain - Cerebellar Hemisphere
ENSG00000214401.4	KANSL1-AS1	1.2e-27	1.1	Brain - Hypothalamus
ENSG00000263503.1	MAPK8IP1P2	1.2e-27	1.1	Brain - Caudate (basal ganglia)
ENSG00000225190.10	PLEKHM1	1.3e-27	-0.84	Brain - Cerebellar Hemisphere
ENSG00000262539.1	RP11-259G18.3	2.0e-27	1.2	Brain - Anterior cingulate cortex (BA24)
ENSG00000238083.7	LRRC37A2	2.4e-27	1.1	Small Intestine - Terminal Ileum
ENSG00000214401.4	KANSL1-AS1	2.9e-27	1.2	Uterus
ENSG00000261575.2	RP11-259G18.1	3.2e-27	0.72	Esophagus - Mucosa
ENSG00000214401.4	KANSL1-AS1	3.8e-27	1.2	Ovary
ENSG00000214401.4	KANSL1-AS1	4.5e-27	1.1	Cells - EBV-transformed lymphocytes
ENSG00000176681.14	LRRC37A	6.3e-27	1.0	Brain - Cerebellum
ENSG00000263503.1	MAPK8IP1P2	9.5e-27	1.2	Brain - Frontal Cortex (BA9)
ENSG00000262500.1	MAPK8IP1P1	1.7e-26	1.2	Brain - Hippocampus
ENSG00000262539.1	RP11-259G18.3	1.9e-26	1.3	Ovary
ENSG00000185829.17	ARL17A	3.2e-26	1.1	Brain - Frontal Cortex (BA9)
ENSG00000262539.1	RP11-259G18.3	3.2e-26	1.2	Brain - Hippocampus
ENSG00000204650.14	LINC02210	4.8e-26	0.56	Brain - Frontal Cortex (BA9)
ENSG00000262539.1	RP11-259G18.3	6.0e-26	1.1	Vagina
ENSG00000263503.1	MAPK8IP1P2	8.3e-26	1.2	Brain - Hippocampus
ENSG00000238083.7	LRRC37A2	9.0e-26	1.1	Brain - Amygdala
ENSG00000214401.4	KANSL1-AS1	1.0e-25	1.1	Brain - Anterior cingulate cortex (BA24)
ENSG00000185829.17	ARL17A	1.1e-25	0.99	Pituitary
ENSG00000261575.2	RP11-259G18.1	2.1e-25	0.68	Pancreas
ENSG00000263503.1	MAPK8IP1P2	2.2e-25	1.2	Brain - Anterior cingulate cortex (BA24)

ENSG00000176681.14	LRRC37A	2.8e-25	1.1	Brain - Cerebellar Hemisphere
ENSG00000186868.15	MAPT	5.4e-25	-0.67	Colon - Sigmoid
ENSG00000262500.1	MAPK8IP1P1	6.9e-25	1.1	Brain - Putamen (basal ganglia)
ENSG00000262500.1	MAPK8IP1P1	8.1e-25	1.0	Vagina
ENSG00000214425.7	LRRC37A4P	8.1e-25	-1.0	Vagina
ENSG00000263503.1	MAPK8IP1P2	9.3e-25	1.2	Brain - Amygdala
ENSG00000264070.1	DND1P1	1.8e-24	1.0	Brain - Caudate (basal ganglia)
ENSG00000225190.10	PLEKHM1	2.1e-24	-0.31	Esophagus - Mucosa
ENSG00000262500.1	MAPK8IP1P1	2.4e-24	0.97	Adrenal Gland
ENSG00000204650.14	LINC02210	2.9e-24	1.1	Brain - Substantia nigra
ENSG00000262500.1	MAPK8IP1P1	3.6e-24	1.2	Brain - Frontal Cortex (BA9)
ENSG00000185294.6	SPPL2C	5.5e-24	0.76	Brain - Cerebellum
ENSG00000238083.7	LRRC37A2	5.7e-24	0.89	Liver
ENSG00000264070.1	DND1P1	8.6e-24	1.1	Vagina
ENSG00000262500.1	MAPK8IP1P1	1.8e-23	1.2	Brain - Hypothalamus
ENSG00000185829.17	ARL17A	2.6e-23	0.99	Brain - Nucleus accumbens (basal ganglia)
ENSG00000262539.1	RP11-259G18.3	2.8e-23	1.2	Brain - Spinal cord (cervical c-1)
ENSG00000262500.1	MAPK8IP1P1	3.7e-23	1.2	Brain - Anterior cingulate cortex (BA24)
ENSG00000264070.1	DND1P1	5.2e-23	1.2	Ovary
ENSG00000120088.14	CRHR1	5.3e-23	0.44	Breast - Mammary Tissue
ENSG00000262539.1	RP11-259G18.3	6.8e-23	1.1	Brain - Amygdala
ENSG00000264070.1	DND1P1	7.4e-23	1.0	Brain - Nucleus accumbens (basal ganglia)
ENSG00000266918.1	RP11-798G7.8	8.9e-23	0.56	Muscle - Skeletal
ENSG00000262500.1	MAPK8IP1P1	1.3e-22	1.1	Ovary
ENSG00000184922.13	FMNL1	1.6e-22	-0.70	Brain - Cerebellum
ENSG00000261575.2	RP11-259G18.1	1.9e-22	0.70	Esophagus - Muscularis
ENSG00000263503.1	MAPK8IP1P2	2.8e-22	1.1	Vagina
ENSG00000232300.1	FAM215B	3.8e-22	0.85	Brain - Cerebellum
ENSG00000120071.13	KANSL1	3.9e-22	0.40	Muscle - Skeletal
ENSG00000214401.4	KANSL1-AS1	4.9e-22	1.1	Brain - Spinal cord (cervical c-1)
ENSG00000261575.2	RP11-259G18.1	1.1e-21	0.67	Nerve - Tibial
ENSG00000186868.15	MAPT	1.1e-21	0.49	Skin - Sun Exposed (Lower leg)
ENSG00000238083.7	LRRC37A2	1.1e-21	1.1	Brain - Spinal cord (cervical c-1)
ENSG00000214425.7	LRRC37A4P	1.1e-21	-1.0	Minor Salivary Gland
ENSG00000261575.2	RP11-259G18.1	1.4e-21	0.85	Brain - Nucleus accumbens (basal ganglia)
ENSG00000263503.1	MAPK8IP1P2	2.0e-21	1.1	Uterus
ENSG00000280022.1	RP11-707O23.1	2.1e-21	1.2	Ovary
ENSG00000176681.14	LRRC37A	2.6e-21	0.60	Adipose - Visceral (Omentum)
ENSG00000238083.7	LRRC37A2	2.8e-21	0.92	Ovary
ENSG00000214401.4	KANSL1-AS1	3.9e-21	1.0	Brain - Amygdala
ENSG00000264070.1	DND1P1	4.6e-21	1.0	Brain - Frontal Cortex (BA9)
ENSG00000185829.17	ARL17A	7.9e-21	1.0	Brain - Hypothalamus

ENSG00000264070.1	DND1P1	9.3e-21	1.0	Brain - Hippocampus
ENSG00000264070.1	DND1P1	9.3e-21	1.0	Brain - Hypothalamus
ENSG00000214425.7	LRRC37A4P	1.3e-20	-1.2	Kidney - Cortex
ENSG00000238083.7	LRRC37A2	1.4e-20	0.91	Uterus
ENSG00000264070.1	DND1P1	1.5e-20	1.1	Cells - EBV-transformed lymphocytes
ENSG00000120088.14	CRHR1	1.6e-20	0.51	Adipose - Visceral (Omentum)
ENSG00000238083.7	LRRC37A2	2.5e-20	1.2	Brain - Substantia nigra
ENSG00000264589.2	MAPT-AS1	2.9e-20	-0.70	Brain - Nucleus accumbens (basal ganglia)
ENSG00000185829.17	ARL17A	3.3e-20	0.69	Cells - Cultured fibroblasts
ENSG00000263503.1	MAPK8IP1P2	5.3e-20	1.1	Ovary
ENSG00000262500.1	MAPK8IP1P1	6.6e-20	1.1	Brain - Amygdala
ENSG00000185294.6	SPPL2C	8.3e-20	0.68	Brain - Cerebellar Hemisphere
ENSG00000264070.1	DND1P1	1.0e-19	-0.51	Testis
ENSG00000261575.2	RP11-259G18.1	1.4e-19	0.58	Thyroid
ENSG00000280022.1	RP11-707O23.1	1.5e-19	1.1	Uterus
ENSG00000261575.2	RP11-259G18.1	2.3e-19	0.77	Esophagus - Gastroesophageal Junction
ENSG00000238083.7	LRRC37A2	2.6e-19	0.89	Vagina
ENSG00000261575.2	RP11-259G18.1	3.6e-19	0.96	Brain - Cortex
ENSG00000238083.7	LRRC37A2	3.9e-19	1.1	Minor Salivary Gland
ENSG00000261575.2	RP11-259G18.1	4.5e-19	0.93	Brain - Caudate (basal ganglia)
ENSG00000184922.13	FMNL1	5.1e-19	-0.64	Brain - Cerebellar Hemisphere
ENSG00000176681.14	LRRC37A	5.8e-19	0.56	Lung
ENSG00000264070.1	DND1P1	6.1e-19	1.1	Brain - Anterior cingulate cortex (BA24)
ENSG00000280022.1	RP11-707O23.1	6.4e-19	0.78	Small Intestine - Terminal Ileum
ENSG00000262500.1	MAPK8IP1P1	1.8e-18	0.99	Uterus
ENSG00000176681.14	LRRC37A	2.9e-18	0.64	Artery - Aorta
ENSG00000159314.11	ARHGAP27	4.1e-18	0.43	Brain - Nucleus accumbens (basal ganglia)
ENSG00000176681.14	LRRC37A	4.2e-18	0.68	Breast - Mammary Tissue
ENSG00000120071.13	KANSL1	5.0e-18	0.35	Esophagus - Mucosa
ENSG00000204650.14	LINC02210	5.8e-18	1.1	Kidney - Cortex
ENSG00000204650.14	LINC02210	9.7e-18	0.72	Cells - EBV-transformed lymphocytes
ENSG00000108379.9	WNT3	1.1e-17	0.72	Pancreas
ENSG00000176681.14	LRRC37A	1.6e-17	0.65	Esophagus - Gastroesophageal Junction
ENSG00000214401.4	KANSL1-AS1	1.7e-17	1.0	Brain - Substantia nigra
ENSG00000274883.1	Metazoa_SRP	1.9e-17	-0.57	Liver
ENSG00000185829.17	ARL17A	2.0e-17	0.59	Esophagus - Muscularis
ENSG00000185829.17	ARL17A	2.2e-17	0.86	Brain - Caudate (basal ganglia)
ENSG00000120088.14	CRHR1	2.2e-17	0.50	Adipose - Subcutaneous
ENSG00000264070.1	DND1P1	3.0e-17	1.1	Brain - Spinal cord (cervical c-1)
ENSG00000185829.17	ARL17A	3.1e-17	0.88	Adrenal Gland
ENSG00000159314.11	ARHGAP27	3.6e-17	-0.17	Esophagus - Mucosa
ENSG00000264070.1	DND1P1	4.0e-17	0.71	Brain - Cerebellum

ENSG00000185829.17	ARL17A	1.1e-16	0.58	Adipose - Visceral (Omentum)
ENSG00000120071.13	KANSL1	1.6e-16	0.27	Cells - Cultured fibroblasts
ENSG00000280022.1	RP11-707O23.1	1.8e-16	0.76	Pituitary
ENSG00000263503.1	MAPK8IP1P2	2.0e-16	1.1	Brain - Spinal cord (cervical c-1)
ENSG00000264070.1	DND1P1	2.3e-16	0.97	Brain - Amygdala
ENSG00000186868.15	MAPT	2.3e-16	-0.48	Heart - Left Ventricle
ENSG00000214401.4	KANSL1-AS1	2.6e-16	1.1	Kidney - Cortex
ENSG00000185829.17	ARL17A	2.6e-16	0.53	Adipose - Subcutaneous
ENSG00000108379.9	WNT3	2.9e-16	0.54	Pituitary
ENSG00000266918.1	RP11-798G7.8	3.0e-16	0.55	Thyroid
ENSG00000261575.2	RP11-259G18.1	4.1e-16	0.74	Pituitary
ENSG00000120071.13	KANSL1	4.1e-16	0.31	Skin - Sun Exposed (Lower leg)
ENSG00000108379.9	WNT3	4.9e-16	0.34	Lung
ENSG00000176681.14	LRRC37A	5.0e-16	0.54	Thyroid
ENSG00000264070.1	DND1P1	5.4e-16	1.0	Uterus
ENSG00000176681.14	LRRC37A	8.1e-16	0.78	Artery - Coronary
ENSG00000185829.17	ARL17A	8.6e-16	0.69	Colon - Transverse
ENSG00000262500.1	MAPK8IP1P1	9.4e-16	1.2	Brain - Substantia nigra
ENSG00000261575.2	RP11-259G18.1	1.0e-15	0.68	Artery - Aorta
ENSG00000261575.2	RP11-259G18.1	1.4e-15	0.95	Brain - Putamen (basal ganglia)
ENSG00000261575.2	RP11-259G18.1	2.0e-15	0.89	Spleen
ENSG00000280022.1	RP11-707O23.1	2.1e-15	0.65	Prostate
ENSG00000176681.14	LRRC37A	2.6e-15	0.67	Colon - Sigmoid
ENSG00000280022.1	RP11-707O23.1	3.4e-15	0.92	Vagina
ENSG00000261575.2	RP11-259G18.1	4.7e-15	0.48	Adipose - Subcutaneous
ENSG00000185829.17	ARL17A	5.2e-15	0.55	Lung
ENSG00000108379.9	WNT3	6.2e-15	0.44	Esophagus - Muscularis
ENSG00000262500.1	MAPK8IP1P1	6.3e-15	1.1	Brain - Spinal cord (cervical c-1)
ENSG00000185829.17	ARL17A	7.5e-15	0.56	Esophagus - Mucosa
ENSG00000176681.14	LRRC37A	8.3e-15	0.47	Whole Blood
ENSG00000185829.17	ARL17A	9.2e-15	0.74	Pancreas
ENSG00000185829.17	ARL17A	9.4e-15	0.67	Stomach
ENSG00000261575.2	RP11-259G18.1	9.6e-15	0.68	Colon - Sigmoid
ENSG00000232300.1	FAM215B	1.2e-14	0.84	Brain - Cerebellar Hemisphere
ENSG00000261575.2	RP11-259G18.1	1.4e-14	0.60	Cells - Cultured fibroblasts
ENSG00000264589.2	MAPT-AS1	1.7e-14	-0.55	Brain - Hypothalamus
ENSG00000108379.9	WNT3	2.1e-14	0.50	Artery - Aorta
ENSG00000120071.13	KANSL1	2.1e-14	0.32	Skin - Not Sun Exposed (Suprapubic)
ENSG00000266918.1	RP11-798G7.8	2.4e-14	0.84	Brain - Cerebellar Hemisphere
ENSG00000176681.14	LRRC37A	3.0e-14	0.48	Muscle - Skeletal
ENSG00000264070.1	DND1P1	3.5e-14	1.1	Brain - Substantia nigra
ENSG00000261575.2	RP11-259G18.1	4.2e-14	0.83	Brain - Frontal Cortex (BA9)

ENSG00000185829.17	ARL17A	4.2e-14	0.64	Heart - Left Ventricle
ENSG00000185829.17	ARL17A	4.4e-14	0.66	Heart - Atrial Appendage
ENSG00000261575.2	RP11-259G18.1	6.5e-14	0.81	Prostate
ENSG00000262539.1	RP11-259G18.3	7.3e-14	1.1	Brain - Substantia nigra
ENSG00000185829.17	ARL17A	8.0e-14	0.65	Colon - Sigmoid
ENSG00000225190.10	PLEKHM1	9.2e-14	0.44	Brain - Cortex
ENSG00000176681.14	LRRC37A	1.1e-13	0.78	Brain - Cortex
ENSG00000261575.2	RP11-259G18.1	1.1e-13	0.64	Heart - Atrial Appendage
ENSG00000279685.2	MAPT-IT1	1.2e-13	0.49	Testis
ENSG00000225190.10	PLEKHM1	1.5e-13	0.41	Brain - Nucleus accumbens (basal ganglia)
ENSG00000261575.2	RP11-259G18.1	1.8e-13	0.84	Brain - Anterior cingulate cortex (BA24)
ENSG00000238083.7	LRRC37A2	2.0e-13	0.88	Cells - EBV-transformed lymphocytes
ENSG00000264589.2	MAPT-AS1	2.0e-13	-0.37	Muscle - Skeletal
ENSG00000185829.17	ARL17A	2.2e-13	0.54	Breast - Mammary Tissue
ENSG00000108379.9	WNT3	3.0e-13	0.43	Nerve - Tibial
ENSG00000120088.14	CRHR1	3.9e-13	0.50	Nerve - Tibial
ENSG00000185829.17	ARL17A	4.6e-13	0.89	Brain - Anterior cingulate cortex (BA24)
ENSG00000261575.2	RP11-259G18.1	5.1e-13	0.48	Artery - Tibial
ENSG00000264070.1	DND1P1	6.1e-13	0.76	Brain - Cerebellar Hemisphere
ENSG00000108379.9	WNT3	6.4e-13	0.36	Artery - Tibial
ENSG00000263503.1	MAPK8IP1P2	7.0e-13	1.1	Kidney - Cortex
ENSG00000108379.9	WNT3	7.1e-13	0.51	Esophagus - Gastroesophageal Junction
ENSG00000176681.14	LRRC37A	7.3e-13	0.89	Brain - Hypothalamus
ENSG00000238083.7	LRRC37A2	9.7e-13	1.0	Kidney - Cortex
ENSG00000185829.17	ARL17A	9.9e-13	0.51	Nerve - Tibial
ENSG00000261575.2	RP11-259G18.1	1.1e-12	0.57	Heart - Left Ventricle
ENSG00000261575.2	RP11-259G18.1	1.6e-12	0.60	Colon - Transverse
ENSG00000176681.14	LRRC37A	1.7e-12	0.70	Uterus
ENSG00000185829.17	ARL17A	2.0e-12	0.44	Skin - Sun Exposed (Lower leg)
ENSG00000185829.17	ARL17A	2.2e-12	0.78	Brain - Putamen (basal ganglia)
ENSG00000176681.14	LRRC37A	2.2e-12	0.75	Prostate
ENSG00000236234.1	AC091132.1	2.6e-12	-0.63	Testis
ENSG00000159314.11	ARHGAP27	2.6e-12	-0.52	Cells - EBV-transformed lymphocytes
ENSG00000263503.1	MAPK8IP1P2	2.7e-12	1.1	Brain - Substantia nigra
ENSG00000176681.14	LRRC37A	3.0e-12	0.73	Pituitary
ENSG00000261575.2	RP11-259G18.1	4.0e-12	0.82	Brain - Amygdala
ENSG00000185829.17	ARL17A	4.4e-12	0.60	Testis
ENSG00000232300.1	FAM215B	5.0e-12	0.52	Esophagus - Muscularis
ENSG00000232300.1	FAM215B	5.2e-12	0.48	Nerve - Tibial
ENSG00000236234.1	AC091132.1	5.9e-12	-0.26	Esophagus - Mucosa
ENSG00000280022.1	RP11-707O23.1	6.3e-12	0.88	Cells - EBV-transformed lymphocytes
ENSG00000108379.9	WNT3	6.8e-12	0.61	Adrenal Gland

ENSG00000120071.13	KANSL1	8.9e-12	0.22	Whole Blood
ENSG00000232300.1	FAM215B	1.4e-11	0.49	Lung
ENSG00000108379.9	WNT3	2.0e-11	0.38	Adipose - Subcutaneous
ENSG00000185829.17	ARL17A	3.1e-11	0.44	Skin - Not Sun Exposed (Suprapubic)
ENSG00000225190.10	PLEKHM1	3.6e-11	0.42	Adrenal Gland
ENSG00000185829.17	ARL17A	3.9e-11	0.44	Artery - Aorta
ENSG00000261575.2	RP11-259G18.1	4.5e-11	0.47	Adipose - Visceral (Omentum)
ENSG00000185829.17	ARL17A	4.7e-11	0.69	Prostate
ENSG00000176681.14	LRRC37A	5.1e-11	0.77	Brain - Frontal Cortex (BA9)
ENSG00000262500.1	MAPK8IP1P1	6.5e-11	1.0	Kidney - Cortex
ENSG00000261575.2	RP11-259G18.1	7.2e-11	0.85	Ovary
ENSG00000280022.1	RP11-707O23.1	7.7e-11	0.84	Brain - Spinal cord (cervical c-1)
ENSG00000236234.1	AC091132.1	9.4e-11	-0.61	Brain - Cerebellum
ENSG00000185829.17	ARL17A	1.0e-10	0.40	Artery - Tibial
ENSG00000280022.1	RP11-707O23.1	1.1e-10	0.71	Spleen
ENSG00000261575.2	RP11-259G18.1	1.1e-10	0.71	Small Intestine - Terminal Ileum
ENSG00000261575.2	RP11-259G18.1	1.2e-10	0.86	Uterus
ENSG00000266918.1	RP11-798G7.8	1.5e-10	0.46	Nerve - Tibial
ENSG00000264070.1	DND1P1	2.0e-10	1.1	Kidney - Cortex
ENSG00000108379.9	WNT3	2.0e-10	0.48	Colon - Sigmoid
ENSG00000264589.2	MAPT-AS1	2.1e-10	-0.60	Brain - Caudate (basal ganglia)
ENSG00000261575.2	RP11-259G18.1	2.1e-10	0.77	Vagina
ENSG00000204652.6	RPS26P8	2.1e-10	0.24	Testis
ENSG00000262539.1	RP11-259G18.3	2.3e-10	1.0	Kidney - Cortex
ENSG00000232300.1	FAM215B	2.9e-10	0.61	Pituitary
ENSG00000120071.13	KANSL1	3.5e-10	0.67	Cells - EBV-transformed lymphocytes
ENSG00000185829.17	ARL17A	3.8e-10	0.68	Brain - Hippocampus
ENSG00000261575.2	RP11-259G18.1	3.8e-10	0.46	Breast - Mammary Tissue
ENSG00000279685.2	MAPT-IT1	4.4e-10	-0.43	Liver
ENSG00000176681.14	LRRC37A	4.8e-10	0.72	Brain - Caudate (basal ganglia)
ENSG00000159314.11	ARHGAP27	4.9e-10	-0.34	Testis
ENSG00000267121.5	CTD-2020K17.1	5.4e-10	-0.55	Brain - Cerebellum
ENSG00000185829.17	ARL17A	6.2e-10	0.82	Brain - Amygdala
ENSG00000108379.9	WNT3	6.9e-10	0.49	Heart - Left Ventricle
ENSG00000176681.14	LRRC37A	7.2e-10	0.91	Brain - Spinal cord (cervical c-1)
ENSG00000120071.13	KANSL1	7.6e-10	0.29	Adipose - Subcutaneous
ENSG00000267198.1	RP11-798G7.6	9.9e-10	-0.49	Esophagus - Mucosa
ENSG00000225190.10	PLEKHM1	1.0e-9	-0.35	Testis
ENSG00000204650.14	LINC02210	1.1e-9	0.31	Brain - Cerebellar Hemisphere
ENSG00000120088.14	CRHR1	1.1e-9	0.38	Esophagus - Muscularis
ENSG00000073969.18	NSF	1.1e-9	-0.16	Esophagus - Mucosa
ENSG00000185829.17	ARL17A	1.2e-9	0.72	Ovary

ENSG00000225190.10	PLEKHM1	1.5e-9	0.16	Esophagus - Muscularis
ENSG00000266918.1	RP11-798G7.8	1.6e-9	0.40	Artery - Tibial
ENSG00000176681.14	LRRC37A	1.7e-9	0.49	Heart - Left Ventricle
ENSG00000186868.15	MAPT	2.1e-9	0.29	Skin - Not Sun Exposed (Suprapubic)
ENSG00000264589.2	MAPT-AS1	2.4e-9	-0.65	Brain - Spinal cord (cervical c-1)
ENSG00000108379.9	WNT3	2.6e-9	0.44	Colon - Transverse
ENSG00000185829.17	ARL17A	2.7e-9	0.65	Liver
ENSG00000176681.14	LRRC37A	3.2e-9	0.72	Brain - Hippocampus
ENSG00000186868.15	MAPT	3.7e-9	-0.32	Artery - Aorta
ENSG00000073969.18	NSF	3.8e-9	0.22	Testis
ENSG00000159314.11	ARHGAP27	4.8e-9	0.24	Brain - Caudate (basal ganglia)
ENSG00000176681.14	LRRC37A	4.8e-9	0.64	Ovary
ENSG00000185829.17	ARL17A	5.4e-9	0.60	Artery - Coronary
ENSG00000266918.1	RP11-798G7.8	5.5e-9	0.39	Adipose - Subcutaneous
ENSG00000108379.9	WNT3	6.1e-9	0.52	Artery - Coronary
ENSG00000266918.1	RP11-798G7.8	6.4e-9	0.57	Brain - Cerebellum
ENSG00000261575.2	RP11-259G18.1	6.8e-9	0.71	Brain - Hypothalamus
ENSG00000176681.14	LRRC37A	7.6e-9	0.65	Brain - Nucleus accumbens (basal ganglia)
ENSG00000279685.2	MAPT-IT1	7.8e-9	0.50	Brain - Cerebellum
ENSG00000204650.14	LINC02210	1.1e-8	0.37	Brain - Cerebellum
ENSG00000266918.1	RP11-798G7.8	1.3e-8	0.49	Colon - Sigmoid
ENSG00000225190.10	PLEKHM1	1.3e-8	0.17	Adipose - Subcutaneous
ENSG00000225190.10	PLEKHM1	1.9e-8	0.11	Cells - Cultured fibroblasts
ENSG00000176681.14	LRRC37A	2.0e-8	0.91	Brain - Substantia nigra
ENSG00000185829.17	ARL17A	2.2e-8	0.46	Esophagus - Gastroesophageal Junction
ENSG00000280022.1	RP11-707O23.1	2.6e-8	-0.52	Brain - Cerebellar Hemisphere
ENSG00000267344.1	CTB-39G8.3	2.9e-8	0.24	Skin - Not Sun Exposed (Suprapubic)
ENSG00000185829.17	ARL17A	3.2e-8	0.69	Spleen
ENSG00000185829.17	ARL17A	3.6e-8	0.70	Uterus
ENSG00000225190.10	PLEKHM1	3.7e-8	0.18	Lung
ENSG00000185829.17	ARL17A	4.2e-8	0.77	Minor Salivary Gland
ENSG00000073969.18	NSF	4.4e-8	0.19	Cells - Cultured fibroblasts
ENSG00000261575.2	RP11-259G18.1	4.4e-8	0.36	Lung
ENSG00000120071.13	KANSL1	4.5e-8	0.37	Colon - Sigmoid
ENSG00000176681.14	LRRC37A	4.7e-8	0.40	Esophagus - Mucosa
ENSG00000225190.10	PLEKHM1	4.7e-8	0.31	Brain - Caudate (basal ganglia)
ENSG00000262372.1	RP11-669E14.6	5.1e-8	-0.39	Testis
ENSG00000261575.2	RP11-259G18.1	5.4e-8	0.72	Cells - EBV-transformed lymphocytes
ENSG00000266918.1	RP11-798G7.8	6.1e-8	0.49	Colon - Transverse
ENSG00000225190.10	PLEKHM1	6.2e-8	0.39	Brain - Anterior cingulate cortex (BA24)
ENSG00000266918.1	RP11-798G7.8	6.7e-8	0.73	Uterus
ENSG00000108379.9	WNT3	6.8e-8	0.31	Testis

ENSG00000266918.1	RP11-798G7.8	7.3e-8	0.47	Esophagus - Gastroesophageal Junction
ENSG00000108379.9	WNT3	7.8e-8	0.40	Heart - Atrial Appendage
ENSG00000261575.2	RP11-259G18.1	7.9e-8	0.24	Muscle - Skeletal
ENSG00000176681.14	LRRC37A	8.6e-8	0.68	Brain - Putamen (basal ganglia)
ENSG00000176681.14	LRRC37A	1.2e-7	0.67	Brain - Anterior cingulate cortex (BA24)
ENSG00000176681.14	LRRC37A	1.3e-7	0.47	Stomach
ENSG00000236234.1	AC091132.1	1.3e-7	-0.56	Brain - Cerebellar Hemisphere
ENSG00000225190.10	PLEKHM1	1.3e-7	0.19	Breast - Mammary Tissue
ENSG00000120071.13	KANSL1	1.7e-7	0.36	Colon - Transverse
ENSG00000120071.13	KANSL1	1.8e-7	0.23	Adipose - Visceral (Omentum)
ENSG00000267344.1	CTB-39G8.3	1.8e-7	-0.17	Esophagus - Mucosa
ENSG00000108379.9	WNT3	2.0e-7	0.33	Stomach
ENSG00000225190.10	PLEKHM1	2.3e-7	0.34	Brain - Putamen (basal ganglia)
ENSG00000186868.15	MAPT	2.3e-7	-0.17	Colon - Transverse
ENSG00000073969.18	NSF	2.4e-7	-0.19	Esophagus - Muscularis
ENSG00000120088.14	CRHR1	2.4e-7	0.25	Muscle - Skeletal
ENSG00000266918.1	RP11-798G7.8	2.6e-7	0.38	Adipose - Visceral (Omentum)
ENSG00000108379.9	WNT3	2.7e-7	0.30	Breast - Mammary Tissue
ENSG00000264589.2	MAPT-AS1	3.0e-7	-0.62	Brain - Putamen (basal ganglia)
ENSG00000232300.1	FAM215B	3.3e-7	0.59	Prostate
ENSG00000185829.17	ARL17A	3.3e-7	0.54	Small Intestine - Terminal Ileum
ENSG00000176681.14	LRRC37A	4.9e-7	0.46	Colon - Transverse
ENSG00000176681.14	LRRC37A	5.1e-7	0.56	Vagina
ENSG00000120071.13	KANSL1	5.4e-7	0.24	Artery - Tibial
ENSG00000176681.14	LRRC37A	6.3e-7	0.68	Brain - Amygdala
ENSG00000266918.1	RP11-798G7.8	6.3e-7	0.39	Artery - Aorta
ENSG00000186868.15	MAPT	6.8e-7	-0.19	Artery - Tibial
ENSG00000261575.2	RP11-259G18.1	7.0e-7	0.56	Brain - Hippocampus
ENSG00000186868.15	MAPT	7.9e-7	-0.29	Brain - Cerebellum
ENSG00000225190.10	PLEKHM1	8.0e-7	0.32	Brain - Frontal Cortex (BA9)
ENSG00000266918.1	RP11-798G7.8	8.1e-7	0.46	Stomach
ENSG00000073969.18	NSF	8.3e-7	-0.20	Heart - Atrial Appendage
ENSG00000204652.6	RPS26P8	9.4e-7	0.72	Ovary
ENSG00000267121.5	CTD-2020K17.1	9.8e-7	-0.44	Brain - Cerebellar Hemisphere
ENSG00000186868.15	MAPT	0.0000010	0.17	Adipose - Visceral (Omentum)
ENSG00000073969.18	NSF	0.0000012	-0.17	Artery - Tibial
ENSG00000073969.18	NSF	0.0000013	-0.17	Artery - Aorta
ENSG00000228696.8	ARL17B	0.0000014	0.36	Skin - Not Sun Exposed (Suprapubic)
ENSG00000176681.14	LRRC37A	0.0000014	0.55	Adrenal Gland
ENSG00000176681.14	LRRC37A	0.0000015	0.62	Small Intestine - Terminal Ileum
ENSG00000176681.14	LRRC37A	0.0000018	0.54	Spleen
ENSG00000073969.18	NSF	0.0000025	-0.25	Ovary

ENSG00000264589.2	MAPT-AS1	0.0000025	-0.23	Brain - Cerebellar Hemisphere
ENSG00000136448.11	NMT1	0.0000026	-0.14	Cells - Cultured fibroblasts
ENSG00000159314.11	ARHGAP27	0.0000028	0.32	Brain - Cortex
ENSG00000225190.10	PLEKHM1	0.0000028	0.18	Adipose - Visceral (Omentum)
ENSG00000120071.13	KANSL1	0.0000028	0.28	Lung
ENSG00000266918.1	RP11-798G7.8	0.0000029	0.49	Pituitary
ENSG00000185829.17	ARL17A	0.0000031	0.28	Whole Blood
ENSG00000261575.2	RP11-259G18.1	0.0000033	0.50	Artery - Coronary
ENSG00000280022.1	RP11-707O23.1	0.0000045	-0.55	Brain - Hypothalamus
ENSG00000120088.14	CRHR1	0.0000045	0.37	Esophagus - Gastroesophageal Junction
ENSG00000136448.11	NMT1	0.0000045	-0.14	Thyroid
ENSG00000232300.1	FAM215B	0.0000046	0.41	Esophagus - Gastroesophageal Junction
ENSG00000267344.1	CTB-39G8.3	0.0000049	-0.39	Testis
ENSG00000232300.1	FAM215B	0.0000056	0.35	Esophagus - Mucosa
ENSG00000176681.14	LRRC37A	0.0000057	0.37	Heart - Atrial Appendage
ENSG00000261575.2	RP11-259G18.1	0.0000065	0.60	Minor Salivary Gland
ENSG00000266918.1	RP11-798G7.8	0.0000079	0.33	Skin - Not Sun Exposed (Suprapubic)
ENSG00000186868.15	MAPT	0.0000082	-0.28	Prostate
ENSG00000266918.1	RP11-798G7.8	0.0000085	0.33	Lung
ENSG00000236234.1	AC091132.1	0.0000090	-0.57	Cells - EBV-transformed lymphocytes
ENSG00000185294.6	SPPL2C	0.0000090	0.37	Brain - Frontal Cortex (BA9)
ENSG00000204650.14	LINC02210	0.0000098	-0.31	Liver
ENSG00000261575.2	RP11-259G18.1	0.000010	0.67	Brain - Substantia nigra
ENSG00000120071.13	KANSL1	0.000011	0.33	Brain - Cerebellum
ENSG00000186868.15	MAPT	0.000011	-0.21	Esophagus - Muscularis
ENSG00000186868.15	MAPT	0.000013	0.46	Spleen
ENSG00000120071.13	KANSL1	0.000013	0.35	Brain - Cerebellar Hemisphere
ENSG00000225190.10	PLEKHM1	0.000014	-0.066	Whole Blood
ENSG00000280022.1	RP11-707O23.1	0.000014	0.47	Brain - Cortex
ENSG00000228696.8	ARL17B	0.000014	0.31	Skin - Sun Exposed (Lower leg)
ENSG00000264589.2	MAPT-AS1	0.000014	-0.67	Brain - Substantia nigra
ENSG00000108379.9	WNT3	0.000015	0.24	Muscle - Skeletal
ENSG00000232300.1	FAM215B	0.000015	0.61	Ovary
ENSG00000265964.1	RP11-293E1.1	0.000015	-0.28	Testis
ENSG00000225190.10	PLEKHM1	0.000017	0.34	Brain - Amygdala
ENSG00000120071.13	KANSL1	0.000018	0.30	Small Intestine - Terminal Ileum
ENSG00000266918.1	RP11-798G7.8	0.000022	0.43	Pancreas
ENSG00000185829.17	ARL17A	0.000026	0.58	Cells - EBV-transformed lymphocytes
ENSG00000172992.11	DCAKD	0.000029	0.19	Artery - Tibial
ENSG00000261575.2	RP11-259G18.1	0.000030	0.63	Brain - Spinal cord (cervical c-1)
ENSG00000228696.8	ARL17B	0.000032	-0.27	Muscle - Skeletal
ENSG00000264589.2	MAPT-AS1	0.000032	-0.31	Brain - Hippocampus

ENSG00000266918.1	RP11-798G7.8	0.000035	0.28	Skin - Sun Exposed (Lower leg)
ENSG00000186868.15	MAPT	0.000035	0.12	Adipose - Subcutaneous
ENSG00000232300.1	FAM215B	0.000039	0.27	Skin - Sun Exposed (Lower leg)
ENSG00000267198.1	RP11-798G7.6	0.000041	-0.29	Skin - Sun Exposed (Lower leg)
ENSG00000232300.1	FAM215B	0.000042	0.34	Breast - Mammary Tissue
ENSG00000120071.13	KANSL1	0.000043	0.37	Pituitary
ENSG00000266918.1	RP11-798G7.8	0.000044	0.42	Brain - Cortex
ENSG00000266918.1	RP11-798G7.8	0.000049	0.37	Heart - Atrial Appendage
ENSG00000185829.17	ARL17A	0.000050	0.48	Vagina
ENSG00000186868.15	MAPT	0.000054	-0.19	Thyroid
ENSG00000266918.1	RP11-798G7.8	0.000054	0.56	Ovary
ENSG00000232300.1	FAM215B	0.000055	0.46	Artery - Coronary
ENSG00000172992.11	DCAKD	0.000057	0.16	Adipose - Subcutaneous
ENSG00000108379.9	WNT3	0.000063	0.24	Adipose - Visceral (Omentum)
ENSG00000120088.14	CRHR1	0.000066	-0.26	Brain - Caudate (basal ganglia)
ENSG00000267198.1	RP11-798G7.6	0.000067	-0.46	Brain - Cerebellum
ENSG00000228696.8	ARL17B	0.000071	-0.27	Whole Blood
ENSG00000120088.14	CRHR1	0.000074	-0.23	Brain - Hippocampus
ENSG00000136448.11	NMT1	0.000079	-0.23	Brain - Cerebellar Hemisphere
ENSG00000120088.14	CRHR1	0.000095	-0.26	Brain - Nucleus accumbens (basal ganglia)
ENSG00000172992.11	DCAKD	0.00011	0.19	Nerve - Tibial
ENSG00000159314.11	ARHGAP27	0.00012	0.12	Artery - Aorta
ENSG00000225190.10	PLEKHM1	0.00015	0.090	Artery - Tibial
ENSG00000266918.1	RP11-798G7.8	0.00017	0.28	Esophagus - Muscularis
ENSG00000260075.1	NSFP1	0.00017	-0.36	Esophagus - Gastroesophageal Junction
ENSG00000073969.18	NSF	0.00017	-0.15	Brain - Cerebellum
ENSG00000185294.6	SPPL2C	0.00020	0.11	Testis
ENSG00000120088.14	CRHR1	0.00021	-0.18	Thyroid
ENSG00000181513.14	ACBD4	0.00021	-0.12	Nerve - Tibial
ENSG00000172992.11	DCAKD	0.00027	0.16	Esophagus - Mucosa
ENSG00000131484.4	RP11-798G7.5	0.00027	-0.25	Thyroid

p-value, eQTL association p value; NES, normalized effect size.

Table S22. sQTL statistics for rs2106786 from GTEx v8 portal.

Gencode Id	Gene Symbol	Phenotype Id	Intron Id	P-Value	NES	Tissue
ENSG00000120071.13	KANSL1	chr17:46094701:46170855:clu_13592:ENSG00000120071.13	46094701:46170855:clu_13592	1.1e-197	-1.8	Artery - Tibial
ENSG00000120071.13	KANSL1	chr17:46094701:46170855:clu_15040:ENSG00000120071.13	46094701:46170855:clu_15040	1.4e-193	-1.7	Skin - Sun Exposed (Lower leg)
ENSG00000120071.13	KANSL1	chr17:46094701:46170855:clu_12990:ENSG00000120071.13	46094701:46170855:clu_12990	6.9e-189	-1.6	Muscle - Skeletal
ENSG00000120071.13	KANSL1	chr17:46094701:46170855:clu_14811:ENSG00000120071.13	46094701:46170855:clu_14811	1.7e-180	-1.7	Adipose - Subcutaneous
ENSG00000120071.13	KANSL1	chr17:46094701:46170855:clu_14811:ENSG00000120071.13	46094701:46170855:clu_14811	1.7e-180	-1.7	Adipose - Subcutaneous
ENSG00000120071.13	KANSL1	chr17:46094701:46170855:clu_15568:ENSG00000120071.13	46094701:46170855:clu_15568	2.2e-177	-1.7	Thyroid
ENSG00000120071.13	KANSL1	chr17:46094701:46170855:clu_14690:ENSG00000120071.13	46094701:46170855:clu_14690	3.0e-170	-1.7	Skin - Not Sun Exposed (Suprapubic)
ENSG00000120071.13	KANSL1	chr17:46094701:46170855:clu_15235:ENSG00000120071.13	46094701:46170855:clu_15235	3.4e-160	-1.8	Nerve - Tibial
ENSG00000120071.13	KANSL1	chr17:46094701:46170855:clu_11699:ENSG00000120071.13	46094701:46170855:clu_11699	4.3e-158	-1.6	Whole Blood
ENSG00000120071.13	KANSL1	chr17:46094701:46170855:clu_14343:ENSG00000120071.13	46094701:46170855:clu_14343	1.5e-150	-1.7	Adipose - Visceral (Omentum)
ENSG00000120071.13	KANSL1	chr17:46094701:46170855:clu_15467:ENSG00000120071.13	46094701:46170855:clu_15467	5.2e-144	-1.7	Lung
ENSG00000120071.13	KANSL1	chr17:46094701:46170855:clu_13509:ENSG00000120071.13	46094701:46170855:clu_13509	2.3e-141	-1.6	Esophagus - Muscularis
ENSG00000186868.15	MAPT	chr17:45991586:45996399:clu_12984:ENSG00000186868.15	45991586:45996399:clu_12984	9.9e-141	-1.4	Muscle - Skeletal
ENSG00000120071.13	KANSL1	chr17:46094701:46170855:clu_13547:ENSG00000120071.13	46094701:46170855:clu_13547	8.6e-135	-1.5	Esophagus - Mucosa
ENSG00000120071.13	KANSL1	chr17:46094701:46170855:clu_14805:ENSG00000120071.13	46094701:46170855:clu_14805	1.7e-126	-1.8	Breast - Mammary Tissue
ENSG00000120071.13	KANSL1	chr17:46094701:46170855:clu_14256:ENSG00000120071.13	46094701:46170855:clu_14256	4.5e-114	-1.7	Colon - Transverse
ENSG00000120071.13	KANSL1	chr17:46094701:46170855:clu_14256:ENSG00000120071.13	46094701:46170855:clu_14256	4.5e-114	-1.7	Colon - Transverse
ENSG00000120071.13	KANSL1	chr17:46094701:46170855:clu_13119:ENSG00000120071.13	46094701:46170855:clu_13119	3.1e-113	-1.6	Artery - Aorta
ENSG00000120071.13	KANSL1	chr17:46094701:46170855:clu_13238:ENSG00000120071.13	46094701:46170855:clu_13238	1.3e-110	-1.7	Colon - Sigmoid
ENSG00000120071.13	KANSL1	chr17:46094701:46170855:clu_12921:ENSG00000120071.13	46094701:46170855:clu_12921	1.8e-109	-1.6	Esophagus - Gastroesophageal Junction
ENSG00000120071.13	KANSL1	chr17:46094701:46170855:clu_12921:ENSG00000120071.13	46094701:46170855:clu_12921	1.8e-109	-1.6	Esophagus - Gastroesophageal Junction
ENSG00000120071.13	KANSL1	chr17:46094701:46170855:clu_11609:ENSG00000120071.13	46094701:46170855:clu_11609	1.2e-93	-1.5	Heart - Left Ventricle
ENSG00000120071.13	KANSL1	chr17:46094701:46170855:clu_13190:ENSG00000120071.13	46094701:46170855:clu_13190	8.1e-85	-1.5	Stomach
ENSG00000186868.15	MAPT	chr17:45985744:45987040:clu_25872:ENSG00000186868.15	45985744:45987040:clu_25872	6.3e-84	-1.5	Testis
ENSG00000120071.13	KANSL1	chr17:46094701:46170855:clu_12954:ENSG00000120071.13	46094701:46170855:clu_12954	1.7e-83	-1.5	Heart - Atrial Appendage
ENSG00000120071.13	KANSL1	chr17:46094701:46170855:clu_13961:ENSG00000120071.13	46094701:46170855:clu_13961	1.6e-76	-1.7	Pituitary
ENSG00000120071.13	KANSL1	chr17:46094701:46170855:clu_12402:ENSG00000120071.13	46094701:46170855:clu_12402	1.3e-74	-1.6	Brain - Cerebellum
ENSG00000204650.14	LINC02210	chr17:45630158:45645901:clu_15027:ENSG00000204650.14	45630158:45645901:clu_15027	2.0e-72	1.1	Skin - Sun Exposed (Lower leg)
ENSG00000120071.13	KANSL1	chr17:46094701:46170855:clu_13420:ENSG00000120071.13	46094701:46170855:clu_13420	2.1e-70	-1.7	Prostate
ENSG00000120071.13	KANSL1	chr17:46094701:46170855:clu_13420:ENSG00000120071.13	46094701:46170855:clu_13420	2.1e-70	-1.7	Prostate
ENSG00000204650.14	LINC02210	chr17:45630158:45645901:clu_14678:ENSG00000204650.14	45630158:45645901:clu_14678	6.1e-70	1.1	Skin - Not Sun Exposed (Suprapubic)
ENSG00000120071.13	KANSL1	chr17:46094701:46170855:clu_25893:ENSG00000120071.13	46094701:46170855:clu_25893	6.0e-69	-1.4	Testis

ENSG00000204650.14	LINC02210	chr17:45630158:45645901:clu_25826:ENSG00000204650.14	45630158:45645901:clu_25826	9.3e-65	1.3	Testis
ENSG00000120071.13	KANSL1	chr17:46094701:46170855:clu_11713:ENSG00000120071.13	46094701:46170855:clu_11713	3.5e-64	-1.6	Adrenal Gland
ENSG00000214425.7	LRRC37A4P	chr17:45510293:45513185:clu_15554:ENSG00000214425.7	45510293:45513185:clu_15554	3.3e-62	-1.2	Thyroid
ENSG00000266504.1	RP11-798G7.4	chr17:45510293:45513185:clu_15554:ENSG00000266504.1	45510293:45513185:clu_15554	3.3e-62	-1.2	Thyroid
ENSG00000120071.13	KANSL1	chr17:46094701:46170855:clu_12290:ENSG00000120071.13	46094701:46170855:clu_12290	5.2e-61	-1.6	Artery - Coronary
ENSG00000120071.13	KANSL1	chr17:46094701:46170855:clu_11789:ENSG00000120071.13	46094701:46170855:clu_11789	3.7e-60	-1.5	Pancreas
ENSG00000120071.13	KANSL1	chr17:46094701:46170855:clu_12631:ENSG00000120071.13	46094701:46170855:clu_12631	1.3e-56	-1.7	Spleen
ENSG00000120071.13	KANSL1	chr17:46094701:46170855:clu_11885:ENSG00000120071.13	46094701:46170855:clu_11885	1.3e-56	-1.7	Brain - Cerebellar Hemisphere
ENSG00000120071.13	KANSL1	chr17:46094701:46170855:clu_11869:ENSG00000120071.13	46094701:46170855:clu_11869	3.2e-54	-1.1	Cells - Cultured fibroblasts
ENSG00000214425.7	LRRC37A4P	chr17:45545676:45584572:clu_15168:ENSG00000214425.7	45545676:45584572:clu_15168	1.8e-53	-1.2	Nerve - Tibial
ENSG00000120071.13	KANSL1	chr17:46094701:46170855:clu_11793:ENSG00000120071.13	46094701:46170855:clu_11793	6.6e-51	-1.5	Brain - Cortex
ENSG00000120071.13	KANSL1	chr17:46094701:46170855:clu_11869:ENSG00000120071.13	46094701:46170855:clu_11869	3.5e-48	-1.9	Minor Salivary Gland
ENSG00000120071.13	KANSL1	chr17:46094701:46170855:clu_11840:ENSG00000120071.13	46094701:46170855:clu_11840	6.2e-48	-1.7	Ovary
ENSG00000204650.14	LINC02210	chr17:45630158:45636283:clu_13534:ENSG00000204650.14	45630158:45636283:clu_13534	5.1e-47	-1.0	Esophagus - Mucosa
ENSG00000120071.13	KANSL1	chr17:46094701:46170855:clu_11396:ENSG00000120071.13	46094701:46170855:clu_11396	6.5e-46	-1.5	Brain - Caudate (basal ganglia)
ENSG00000120071.13	KANSL1	chr17:46094701:46170855:clu_13074:ENSG00000120071.13	46094701:46170855:clu_13074	7.7e-46	-1.6	Small Intestine - Terminal Ileum
ENSG00000214425.7	LRRC37A4P	chr17:45482525:45490652:clu_12383:ENSG00000214425.7	45482525:45490652:clu_12383	1.1e-45	1.2	Brain - Cerebellum
ENSG00000225190.10	PLEKHM1	chr17:45482525:45490652:clu_12383:ENSG00000225190.10	45482525:45490652:clu_12383	1.1e-45	1.2	Brain - Cerebellum
ENSG00000120071.13	KANSL1	chr17:46094701:46170855:clu_11663:ENSG00000120071.13	46094701:46170855:clu_11663	4.6e-45	-1.5	Brain - Nucleus accumbens (basal ganglia)
ENSG00000120071.13	KANSL1	chr17:46094701:46170855:clu_10441:ENSG00000120071.13	46094701:46170855:clu_10441	6.8e-45	-1.6	Cells - EBV-transformed lymphocytes
ENSG00000214425.7	LRRC37A4P	chr17:45510293:45513185:clu_15024:ENSG00000214425.7	45510293:45513185:clu_15024	8.1e-43	-0.84	Skin - Sun Exposed (Lower leg)
ENSG00000214425.7	LRRC37A4P	chr17:45510293:45513185:clu_15454:ENSG00000214425.7	45510293:45513185:clu_15454	1.3e-42	-1.0	Lung
ENSG00000214425.7	LRRC37A4P	chr17:45545676:45584572:clu_12976:ENSG00000214425.7	45545676:45584572:clu_12976	7.0e-41	-0.94	Muscle - Skeletal
ENSG00000120071.13	KANSL1	chr17:46094701:46170855:clu_11124:ENSG00000120071.13	46094701:46170855:clu_11124	1.7e-40	-1.6	Uterus
ENSG00000120071.13	KANSL1	chr17:46094701:46170855:clu_10194:ENSG00000120071.13	46094701:46170855:clu_10194	1.5e-38	-1.4	Brain - Putamen (basal ganglia)
ENSG00000120071.13	KANSL1	chr17:46094701:46170855:clu_10194:ENSG00000120071.13	46094701:46170855:clu_10194	1.5e-38	-1.4	Brain - Putamen (basal ganglia)
ENSG00000120071.13	KANSL1	chr17:46094701:46170855:clu_11684:ENSG00000120071.13	46094701:46170855:clu_11684	1.9e-38	-1.6	Vagina
ENSG00000120071.13	KANSL1	chr17:46094701:46170855:clu_10943:ENSG00000120071.13	46094701:46170855:clu_10943	3.2e-37	-1.4	Brain - Frontal Cortex (BA9)
ENSG00000214425.7	LRRC37A4P	chr17:45545676:45584572:clu_13578:ENSG00000214425.7	45545676:45584572:clu_13578	1.0e-36	-0.98	Artery - Tibial
ENSG00000204650.14	LINC02210	chr17:45630158:45645901:clu_13498:ENSG00000204650.14	45630158:45645901:clu_13498	4.0e-34	0.87	Esophagus - Muscularis
ENSG00000214425.7	LRRC37A4P	chr17:45482525:45490652:clu_11865:ENSG00000214425.7	45482525:45490652:clu_11865	4.2e-34	1.2	Brain - Cerebellar Hemisphere
ENSG00000214425.7	LRRC37A4P	chr17:45482525:45490652:clu_11865:ENSG00000214425.7	45482525:45490652:clu_11865	4.2e-34	1.2	Brain - Cerebellar Hemisphere
ENSG00000225190.10	PLEKHM1	chr17:45482525:45490652:clu_11865:ENSG00000225190.10	45482525:45490652:clu_11865	4.2e-34	1.2	Brain - Cerebellar Hemisphere

ENSG00000214425.7	LRRC37A4P	chr17:45551537:45584572:clu_11854:ENSG00000214425.7	45551537:45584572:clu_11854	2.7e-33	-0.94	Cells - Cultured fibroblasts
ENSG00000214425.7	LRRC37A4P	chr17:45550732:45551440:clu_13531:ENSG00000214425.7	45550732:45551440:clu_13531	5.7e-33	-0.88	Esophagus - Mucosa
ENSG00000266504.1	RP11-798G7.4	chr17:45550732:45551440:clu_13531:ENSG00000266504.1	45550732:45551440:clu_13531	5.7e-33	-0.88	Esophagus - Mucosa
ENSG00000204650.14	LINC02210	chr17:45630158:45636283:clu_11857:ENSG00000204650.14	45630158:45636283:clu_11857	7.4e-33	-0.94	Cells - Cultured fibroblasts
ENSG00000204650.14	LINC02210	chr17:45630158:45645901:clu_11870:ENSG00000204650.14	45630158:45645901:clu_11870	1.3e-32	1.2	Brain - Cerebellar Hemisphere
ENSG00000214425.7	LRRC37A4P	chr17:45510293:45513185:clu_14675:ENSG00000214425.7	45510293:45513185:clu_14675	2.8e-32	-0.80	Skin - Not Sun Exposed (Suprapubic)
ENSG00000214425.7	LRRC37A4P	chr17:45510293:45513185:clu_14675:ENSG00000214425.7	45510293:45513185:clu_14675	2.8e-32	-0.80	Skin - Not Sun Exposed (Suprapubic)
ENSG00000120071.13	KANSL1	chr17:46094701:46170855:clu_11097:ENSG00000120071.13	46094701:46170855:clu_11097	3.1e-32	-1.4	Brain - Hypothalamus
ENSG00000204650.14	LINC02210	chr17:45630158:45636283:clu_15557:ENSG00000204650.14	45630158:45636283:clu_15557	2.2e-31	-0.82	Thyroid
ENSG00000120071.13	KANSL1	chr17:46094701:46170855:clu_10078:ENSG00000120071.13	46094701:46170855:clu_10078	3.8e-31	-1.4	Brain - Anterior cingulate cortex (BA24)
ENSG00000214425.7	LRRC37A4P	chr17:45510293:45513185:clu_14332:ENSG00000214425.7	45510293:45513185:clu_14332	4.0e-31	-0.91	Adipose - Visceral (Omentum)
ENSG00000120071.13	KANSL1	chr17:46094701:46170855:clu_10089:ENSG00000120071.13	46094701:46170855:clu_10089	3.0e-29	-1.3	Liver
ENSG00000120071.13	KANSL1	chr17:46094701:46170855:clu_9945:ENSG00000120071.13	46094701:46170855:clu_9945	3.3e-29	-1.4	Brain - Spinal cord (cervical c-1)
ENSG00000214425.7	LRRC37A4P	chr17:45545676:45584572:clu_12906:ENSG00000214425.7	45545676:45584572:clu_12906	3.7e-29	-1.1	Esophagus - Gastroesophageal Junction
ENSG00000204650.14	LINC02210	chr17:45630158:45636283:clu_13945:ENSG00000204650.14	45630158:45636283:clu_13945	8.2e-29	-1.0	Pituitary
ENSG00000214425.7	LRRC37A4P	chr17:45510293:45513185:clu_13105:ENSG00000214425.7	45510293:45513185:clu_13105	8.4e-28	-0.92	Artery - Aorta
ENSG00000120071.13	KANSL1	chr17:46094701:46170855:clu_10387:ENSG00000120071.13	46094701:46170855:clu_10387	1.0e-27	-1.3	Brain - Hippocampus
ENSG00000120071.13	KANSL1	chr17:46094701:46170855:clu_9365:ENSG00000120071.13	46094701:46170855:clu_9365	1.4e-25	-1.3	Brain - Amygdala
ENSG00000186868.15	MAPT	chr17:45974471:45978375:clu_13537:ENSG00000186868.15	45974471:45978375:clu_13537	1.5e-25	-0.69	Esophagus - Mucosa
ENSG00000225190.10	PLEKHM1	chr17:45458439:45475100:clu_15014:ENSG00000225190.10	45458439:45475100:clu_15014	1.2e-24	-0.69	Skin - Sun Exposed (Lower leg)
ENSG00000204650.14	LINC02210	chr17:45630158:45645901:clu_12910:ENSG00000204650.14	45630158:45645901:clu_12910	1.5e-23	0.82	Esophagus - Gastroesophageal Junction
ENSG00000204650.14	LINC02210	chr17:45630158:45636283:clu_15224:ENSG00000204650.14	45630158:45636283:clu_15224	1.6e-23	-0.80	Nerve - Tibial
ENSG00000120088.14	CRHR1	chr17:45630158:45645901:clu_12388:ENSG00000120088.14	45630158:45645901:clu_12388	9.5e-23	0.83	Brain - Cerebellum
ENSG00000204650.14	LINC02210	chr17:45630158:45645901:clu_12388:ENSG00000204650.14	45630158:45645901:clu_12388	9.5e-23	0.83	Brain - Cerebellum
ENSG00000204650.14	LINC02210	chr17:45630158:45645901:clu_13227:ENSG00000204650.14	45630158:45645901:clu_13227	1.4e-22	0.81	Colon - Sigmoid
ENSG00000120088.14	CRHR1	chr17:45630158:45645901:clu_10929:ENSG00000120088.14	45630158:45645901:clu_10929	1.7e-21	1.1	Brain - Frontal Cortex (BA9)
ENSG00000204650.14	LINC02210	chr17:45630158:45645901:clu_10929:ENSG00000204650.14	45630158:45645901:clu_10929	1.7e-21	1.1	Brain - Frontal Cortex (BA9)
ENSG00000214425.7	LRRC37A4P	chr17:45545676:45584572:clu_25854:ENSG00000214425.7	45545676:45584572:clu_25854	3.3e-21	0.85	Testis
ENSG00000204650.14	LINC02210	chr17:45630158:45636283:clu_14335:ENSG00000204650.14	45630158:45636283:clu_14335	6.9e-21	-0.80	Adipose - Visceral (Omentum)
ENSG00000225190.10	PLEKHM1	chr17:45458439:45475100:clu_14793:ENSG00000225190.10	45458439:45475100:clu_14793	2.6e-20	-0.63	Adipose - Subcutaneous
ENSG00000204650.14	LINC02210	chr17:45630158:45636283:clu_13582:ENSG00000204650.14	45630158:45636283:clu_13582	3.1e-20	-0.71	Artery - Tibial
ENSG00000214425.7	LRRC37A4P	chr17:45551537:45584572:clu_13405:ENSG00000214425.7	45551537:45584572:clu_13405	2.5e-18	-0.98	Prostate
ENSG00000214425.7	LRRC37A4P	chr17:45545676:45584572:clu_11685:ENSG00000214425.7	45545676:45584572:clu_11685	3.5e-18	0.62	Whole Blood

ENSG00000225190.10	PLEKHM1	chr17:45458439:45475100:clu_15163:ENSG00000225190.10	45458439:45475100:clu_15163	3.7e-18	-0.63	Nerve - Tibial
ENSG00000186868.15	MAPT	chr17:45974471:45978375:clu_12394:ENSG00000186868.15	45974471:45978375:clu_12394	6.5e-18	0.93	Brain - Cerebellum
ENSG00000204650.14	LINC02210	chr17:45630158:45636283:clu_11830:ENSG00000204650.14	45630158:45636283:clu_11830	8.4e-18	-1.3	Ovary
ENSG00000120088.14	CRHR1	chr17:45630158:45645901:clu_11779:ENSG00000120088.14	45630158:45645901:clu_11779	1.7e-17	0.86	Brain - Cortex
ENSG00000204650.14	LINC02210	chr17:45630158:45645901:clu_11779:ENSG00000204650.14	45630158:45645901:clu_11779	1.7e-17	0.86	Brain - Cortex
ENSG00000214425.7	LRRC37A4P	chr17:45545676:45584572:clu_12941:ENSG00000214425.7	45545676:45584572:clu_12941	2.7e-17	-0.85	Heart - Atrial Appendage
ENSG00000238083.7	LRRC37A2	chr17:46487140:46517362:clu_13004:ENSG00000238083.7	46487140:46517362:clu_13004	6.6e-17	0.56	Muscle - Skeletal
ENSG00000204650.14	LINC02210	chr17:45630158:45636283:clu_14788:ENSG00000204650.14	45630158:45636283:clu_14788	7.1e-17	-0.77	Breast - Mammary Tissue
ENSG00000214425.7	LRRC37A4P	chr17:45551537:45584572:clu_13942:ENSG00000214425.7	45551537:45584572:clu_13942	8.0e-17	-0.89	Pituitary
ENSG00000120088.14	CRHR1	chr17:45630158:45645901:clu_11650:ENSG00000120088.14	45630158:45645901:clu_11650	4.5e-16	0.95	Brain - Nucleus accumbens (basal ganglia)
ENSG00000204650.14	LINC02210	chr17:45630158:45645901:clu_11650:ENSG00000204650.14	45630158:45645901:clu_11650	4.5e-16	0.95	Brain - Nucleus accumbens (basal ganglia)
ENSG00000120088.14	CRHR1	chr17:45630158:45636283:clu_11383:ENSG00000120088.14	45630158:45636283:clu_11383	1.4e-15	-0.92	Brain - Caudate (basal ganglia)
ENSG00000120088.14	CRHR1	chr17:45630158:45636283:clu_11383:ENSG00000120088.14	45630158:45636283:clu_11383	1.4e-15	-0.92	Brain - Caudate (basal ganglia)
ENSG00000204650.14	LINC02210	chr17:45630158:45636283:clu_11383:ENSG00000204650.14	45630158:45636283:clu_11383	1.4e-15	-0.92	Brain - Caudate (basal ganglia)
ENSG00000186868.15	MAPT	chr17:45978440:45987040:clu_13230:ENSG00000186868.15	45978440:45987040:clu_13230	1.7e-15	0.66	Colon - Sigmoid
ENSG00000185829.17	ARL17A	chr17:46528859:46570759:clu_12932:ENSG00000185829.17	46528859:46570759:clu_12932	2.3e-15	0.83	Esophagus - Gastroesophageal Junction
ENSG00000186868.15	MAPT	chr17:45987095:45991460:clu_11659:ENSG00000186868.15	45987095:45991460:clu_11659	4.1e-15	-0.57	Brain - Nucleus accumbens (basal ganglia)
ENSG00000120071.13	KANSL1	chr17:46094701:46170855:clu_8959:ENSG00000120071.13	46094701:46170855:clu_8959	6.8e-15	-1.2	Brain - Substantia nigra
ENSG00000214425.7	LRRC37A4P	chr17:45545676:45584572:clu_14798:ENSG00000214425.7	45545676:45584572:clu_14798	7.6e-15	-0.60	Adipose - Subcutaneous
ENSG00000204650.14	LINC02210	chr17:45630158:45645901:clu_13407:ENSG00000204650.14	45630158:45645901:clu_13407	1.2e-14	0.77	Prostate
ENSG00000225190.10	PLEKHM1	chr17:45458439:45475100:clu_12900:ENSG00000225190.10	45458439:45475100:clu_12900	2.3e-14	-0.69	Esophagus - Gastroesophageal Junction
ENSG00000204650.14	LINC02210	chr17:45630158:45636283:clu_14245:ENSG00000204650.14	45630158:45636283:clu_14245	2.7e-14	-0.71	Colon - Transverse
ENSG00000120088.14	CRHR1	chr17:45630158:45645901:clu_11085:ENSG00000120088.14	45630158:45645901:clu_11085	3.0e-14	0.89	Brain - Hypothalamus
ENSG00000204650.14	LINC02210	chr17:45630158:45645901:clu_11085:ENSG00000204650.14	45630158:45645901:clu_11085	3.0e-14	0.89	Brain - Hypothalamus
ENSG00000204650.14	LINC02210	chr17:45630158:45636283:clu_15457:ENSG00000204650.14	45630158:45636283:clu_15457	5.0e-14	-0.63	Lung
ENSG00000120071.13	KANSL1	chr17:46094701:46170855:clu_8625:ENSG00000120071.13	46094701:46170855:clu_8625	5.7e-14	-1.3	Kidney - Cortex
ENSG00000225190.10	PLEKHM1	chr17:45458439:45475100:clu_15548:ENSG00000225190.10	45458439:45475100:clu_15548	1.3e-13	-0.52	Thyroid
ENSG00000214425.7	LRRC37A4P	chr17:45545676:45584572:clu_11064:ENSG00000214425.7	45545676:45584572:clu_11064	1.4e-13	-1.2	Uterus
ENSG00000204650.14	LINC02210	chr17:45639520:45640389:clu_12979:ENSG00000204650.14	45639520:45640389:clu_12979	1.4e-13	-0.49	Muscle - Skeletal
ENSG00000159314.11	ARHGAP27	chr17:45398047:45402714:clu_15006:ENSG00000159314.11	45398047:45402714:clu_15006	1.9e-13	-0.53	Skin - Sun Exposed (Lower leg)
ENSG00000159314.11	ARHGAP27	chr17:45398047:45402714:clu_15006:ENSG00000159314.11	45398047:45402714:clu_15006	1.9e-13	-0.53	Skin - Sun Exposed (Lower leg)
ENSG00000204650.14	LINC02210	chr17:45630158:45636283:clu_12944:ENSG00000204650.14	45630158:45636283:clu_12944	2.0e-13	-0.67	Heart - Atrial Appendage

ENSG00000204650.14	LINC02210	chr17:45630158:45636283:clu_13179:ENSG00000204650.14	45630158:45636283:clu_13179	2.3e-13	-0.72	Stomach
ENSG00000204650.14	LINC02210	chr17:45630158:45636283:clu_10067:ENSG00000204650.14	45630158:45636283:clu_10067	4.7e-13	-0.94	Brain - Anterior cingulate cortex (BA24)
ENSG00000204650.14	LINC02210	chr17:45630158:45636283:clu_9935:ENSG00000204650.14	45630158:45636283:clu_9935	6.1e-13	-1.0	Brain - Spinal cord (cervical c-1)
ENSG00000204650.14	LINC02210	chr17:45630158:45636283:clu_14801:ENSG00000204650.14	45630158:45636283:clu_14801	6.2e-13	-0.55	Adipose - Subcutaneous
ENSG00000225190.10	PLEKHM1	chr17:45458439:45475100:clu_12973:ENSG00000225190.10	45458439:45475100:clu_12973	7.2e-13	-0.42	Muscle - Skeletal
ENSG00000225190.10	PLEKHM1	chr17:45458439:45475100:clu_11680:ENSG00000225190.10	45458439:45475100:clu_11680	8.4e-13	-0.34	Whole Blood
ENSG00000214425.7	LRRC37A4P	chr17:45545676:45584572:clu_11827:ENSG00000214425.7	45545676:45584572:clu_11827	2.0e-12	-1.1	Ovary
ENSG00000204650.14	LINC02210	chr17:45630158:45636283:clu_11703:ENSG00000204650.14	45630158:45636283:clu_11703	2.3e-12	-0.86	Adrenal Gland
ENSG00000120088.14	CRHR1	chr17:45630158:45636283:clu_10184:ENSG00000120088.14	45630158:45636283:clu_10184	3.9e-12	-0.81	Brain - Putamen (basal ganglia)
ENSG00000204650.14	LINC02210	chr17:45630158:45636283:clu_10184:ENSG00000204650.14	45630158:45636283:clu_10184	3.9e-12	-0.81	Brain - Putamen (basal ganglia)
ENSG00000204650.14	LINC02210	chr17:45630158:45636283:clu_13108:ENSG00000204650.14	45630158:45636283:clu_13108	4.4e-12	-0.63	Artery - Aorta
ENSG00000225190.10	PLEKHM1	chr17:45458439:45475100:clu_14781:ENSG00000225190.10	45458439:45475100:clu_14781	4.6e-12	-0.63	Breast - Mammary Tissue
ENSG00000185829.17	ARL17A	chr17:46528859:46570759:clu_15246:ENSG00000185829.17	46528859:46570759:clu_15246	5.3e-12	0.59	Nerve - Tibial
ENSG00000204650.14	LINC02210	chr17:45630158:45636283:clu_11111:ENSG00000204650.14	45630158:45636283:clu_11111	9.7e-12	-1.0	Uterus
ENSG00000204650.14	LINC02210	chr17:45630158:45636283:clu_10436:ENSG00000204650.14	45630158:45636283:clu_10436	1.0e-11	-1.0	Cells - EBV-transformed lymphocytes
ENSG00000204650.14	LINC02210	chr17:45630158:45645901:clu_11672:ENSG00000204650.14	45630158:45645901:clu_11672	1.6e-11	0.85	Vagina
ENSG00000186868.15	MAPT	chr17:45987095:45991460:clu_11391:ENSG00000186868.15	45987095:45991460:clu_11391	1.6e-11	-0.66	Brain - Caudate (basal ganglia)
ENSG00000225190.10	PLEKHM1	chr17:45482525:45490652:clu_25849:ENSG00000225190.10	45482525:45490652:clu_25849	1.7e-11	0.64	Testis
ENSG00000225190.10	PLEKHM1	chr17:45458439:45475100:clu_13489:ENSG00000225190.10	45458439:45475100:clu_13489	3.4e-11	-0.46	Esophagus - Muscularis
ENSG00000186868.15	MAPT	chr17:45971945:45974385:clu_14682:ENSG00000186868.15	45971945:45974385:clu_14682	3.4e-11	-0.48	Skin - Not Sun Exposed (Suprapubic)
ENSG00000225190.10	PLEKHM1	chr17:45458439:45475100:clu_13572:ENSG00000225190.10	45458439:45475100:clu_13572	4.0e-11	-0.45	Artery - Tibial
ENSG00000185829.17	ARL17A	chr17:46528859:46570759:clu_15480:ENSG00000185829.17	46528859:46570759:clu_15480	4.2e-11	0.57	Lung
ENSG00000214425.7	LRRC37A4P	chr17:45545676:45584572:clu_14785:ENSG00000214425.7	45545676:45584572:clu_14785	8.3e-11	-0.62	Breast - Mammary Tissue
ENSG00000185829.17	ARL17A	chr17:46528859:46570759:clu_14827:ENSG00000185829.17	46528859:46570759:clu_14827	1.3e-10	0.51	Adipose - Subcutaneous
ENSG00000186868.15	MAPT	chr17:45971945:45974385:clu_15031:ENSG00000186868.15	45971945:45974385:clu_15031	1.4e-10	-0.45	Skin - Sun Exposed (Lower leg)
ENSG00000159314.11	ARHGAP27	chr17:45398047:45402714:clu_14658:ENSG00000159314.11	45398047:45402714:clu_14658	1.6e-10	-0.51	Skin - Not Sun Exposed (Suprapubic)
ENSG00000186868.15	MAPT	chr17:45974471:45978375:clu_13952:ENSG00000186868.15	45974471:45978375:clu_13952	2.4e-10	-0.67	Pituitary
ENSG00000185829.17	ARL17A	chr17:46528859:46570759:clu_15580:ENSG00000185829.17	46528859:46570759:clu_15580	2.6e-10	0.51	Thyroid
ENSG00000238083.7	LRRC37A2	chr17:46487140:46511511:clu_11877:ENSG00000238083.7	46487140:46511511:clu_11877	3.7e-10	-0.55	Cells - Cultured fibroblasts
ENSG00000120088.14	CRHR1	chr17:45630158:45645901:clu_10376:ENSG00000120088.14	45630158:45645901:clu_10376	5.8e-10	0.76	Brain - Hippocampus
ENSG00000120088.14	CRHR1	chr17:45630158:45645901:clu_10376:ENSG00000120088.14	45630158:45645901:clu_10376	5.8e-10	0.76	Brain - Hippocampus
ENSG00000204650.14	LINC02210	chr17:45630158:45645901:clu_10376:ENSG00000204650.14	45630158:45645901:clu_10376	5.8e-10	0.76	Brain - Hippocampus
ENSG00000185829.17	ARL17A	chr17:46528859:46570759:clu_11132:ENSG00000185829.17	46528859:46570759:clu_11132	5.9e-10	0.96	Uterus

ENSG00000225190.10	PLEKHM1	chr17:45458439:45475100:clu_11693:ENSG00000225190.10	45458439:45475100:clu_11693	8.7e-10	-0.60	Adrenal Gland
ENSG00000225190.10	PLEKHM1	chr17:45458439:45475100:clu_13101:ENSG00000225190.10	45458439:45475100:clu_13101	1.0e-9	-0.52	Artery - Aorta
ENSG00000204650.14	LINC02210	chr17:45630158:45636283:clu_11859:ENSG00000204650.14	45630158:45636283:clu_11859	1.4e-9	-0.98	Minor Salivary Gland
ENSG00000185829.17	ARL17A	chr17:46528859:46570759:clu_14265:ENSG00000185829.17	46528859:46570759:clu_14265	1.6e-9	0.62	Colon - Transverse
ENSG00000204650.14	LINC02210	chr17:45630158:45636283:clu_11689:ENSG00000204650.14	45630158:45636283:clu_11689	1.8e-9	-0.43	Whole Blood
ENSG00000186868.15	MAPT	chr17:45971945:45974385:clu_11787:ENSG00000186868.15	45971945:45974385:clu_11787	2.0e-9	0.70	Brain - Cortex
ENSG00000214425.7	LRRC37A4P	chr17:45545676:45584572:clu_11700:ENSG00000214425.7	45545676:45584572:clu_11700	2.1e-9	-0.76	Adrenal Gland
ENSG00000225190.10	PLEKHM1	chr17:45458439:45475100:clu_15449:ENSG00000225190.10	45458439:45475100:clu_15449	2.3e-9	-0.42	Lung
ENSG00000120088.14	CRHR1	chr17:45630158:45645901:clu_9353:ENSG00000120088.14	45630158:45645901:clu_9353	2.3e-9	0.84	Brain - Amygdala
ENSG00000204650.14	LINC02210	chr17:45630158:45645901:clu_9353:ENSG00000204650.14	45630158:45645901:clu_9353	2.3e-9	0.84	Brain - Amygdala
ENSG00000204650.14	LINC02210	chr17:45630158:45636283:clu_11777:ENSG00000204650.14	45630158:45636283:clu_11777	3.5e-9	-0.61	Pancreas
ENSG00000225190.10	PLEKHM1	chr17:45458439:45475100:clu_11850:ENSG00000225190.10	45458439:45475100:clu_11850	4.0e-9	-0.45	Cells - Cultured fibroblasts
ENSG00000185829.17	ARL17A	chr17:46528859:46570759:clu_13603:ENSG00000185829.17	46528859:46570759:clu_13603	6.5e-9	0.47	Artery - Tibial
ENSG00000238083.7	LRRC37A2	chr17:46487140:46511511:clu_10450:ENSG00000238083.7	46487140:46511511:clu_10450	6.9e-9	-0.97	Cells - EBV-transformed lymphocytes
ENSG00000159314.11	ARHGAP27	chr17:45398047:45402714:clu_15213:ENSG00000159314.11	45398047:45402714:clu_15213	9.3e-9	-0.48	Nerve - Tibial
ENSG00000186868.15	MAPT	chr17:45971945:45974385:clu_10937:ENSG00000186868.15	45971945:45974385:clu_10937	9.3e-9	0.57	Brain - Frontal Cortex (BA9)
ENSG00000225190.10	PLEKHM1	chr17:45458439:45475100:clu_14666:ENSG00000225190.10	45458439:45475100:clu_14666	1.1e-8	-0.41	Skin - Not Sun Exposed (Suprapubic)
ENSG00000204650.14	LINC02210	chr17:45630158:45636283:clu_12279:ENSG00000204650.14	45630158:45636283:clu_12279	1.3e-8	-0.71	Artery - Coronary
ENSG00000185829.17	ARL17A	chr17:46528859:46570759:clu_13248:ENSG00000185829.17	46528859:46570759:clu_13248	1.4e-8	0.61	Colon - Sigmoid
ENSG00000185829.17	ARL17A	chr17:46528859:46570759:clu_14811:ENSG00000185829.17	46528859:46570759:clu_14811	1.6e-8	0.56	Breast - Mammary Tissue
ENSG00000185829.17	ARL17A	chr17:46528859:46570759:clu_13129:ENSG00000185829.17	46528859:46570759:clu_13129	1.7e-8	0.54	Artery - Aorta
ENSG00000159314.11	ARHGAP27	chr17:45398047:45402714:clu_12371:ENSG00000159314.11	45398047:45402714:clu_12371	2.2e-8	-0.65	Brain - Cerebellum
ENSG00000225190.10	PLEKHM1	chr17:45458439:45475100:clu_14232:ENSG00000225190.10	45458439:45475100:clu_14232	2.9e-8	-0.46	Colon - Transverse
ENSG00000186868.15	MAPT	chr17:45987095:45991460:clu_10074:ENSG00000186868.15	45987095:45991460:clu_10074	3.0e-8	-0.51	Brain - Anterior cingulate cortex (BA24)
ENSG00000186868.15	MAPT	chr17:45978440:45987040:clu_14248:ENSG00000186868.15	45978440:45987040:clu_14248	3.7e-8	0.49	Colon - Transverse
ENSG00000185829.17	ARL17A	chr17:46528859:46570759:clu_14355:ENSG00000185829.17	46528859:46570759:clu_14355	4.8e-8	0.47	Adipose - Visceral (Omentum)
ENSG00000186868.15	MAPT	chr17:45962470:45964397:clu_11877:ENSG00000186868.15	45962470:45964397:clu_11877	5.2e-8	-0.57	Brain - Cerebellar Hemisphere
ENSG00000185829.17	ARL17A	chr17:46528859:46570759:clu_13199:ENSG00000185829.17	46528859:46570759:clu_13199	5.5e-8	0.59	Stomach
ENSG00000186868.15	MAPT	chr17:45987095:45991460:clu_12948:ENSG00000186868.15	45987095:45991460:clu_12948	7.1e-8	-0.56	Heart - Atrial Appendage
ENSG00000225190.10	PLEKHM1	chr17:45458439:45475100:clu_14328:ENSG00000225190.10	45458439:45475100:clu_14328	7.2e-8	-0.43	Adipose - Visceral (Omentum)
ENSG00000120088.14	CRHR1	chr17:45630158:45645901:clu_8948:ENSG00000120088.14	45630158:45645901:clu_8948	7.6e-8	0.99	Brain - Substantia nigra
ENSG00000204650.14	LINC02210	chr17:45630158:45645901:clu_8948:ENSG00000204650.14	45630158:45645901:clu_8948	7.6e-8	0.99	Brain - Substantia nigra
ENSG00000159314.11	ARHGAP27	chr17:45398047:45402714:clu_11854:ENSG00000159314.11	45398047:45402714:clu_11854	1.1e-7	-0.73	Brain - Cerebellar Hemisphere

ENSG00000214425.7	LRRC37A4P	chr17:45545676:45584572:clu_11379:ENSG00000214425.7	45545676:45584572:clu_11379	1.5e-7	-0.72	Brain - Caudate (basal ganglia)
ENSG00000238083.7	LRRC37A2	chr17:46487140:46517362:clu_13966:ENSG00000238083.7	46487140:46517362:clu_13966	1.5e-7	0.63	Pituitary
ENSG00000185829.17	ARL17A	chr17:46528859:46570759:clu_13519:ENSG00000185829.17	46528859:46570759:clu_13519	1.6e-7	0.45	Esophagus - Muscularis
ENSG00000225190.10	PLEKHM1	chr17:45458439:45475100:clu_11078:ENSG00000225190.10	45458439:45475100:clu_11078	1.8e-7	-0.65	Brain - Hypothalamus
ENSG00000185829.17	ARL17A	chr17:46528859:46570759:clu_12639:ENSG00000185829.17	46528859:46570759:clu_12639	1.9e-7	0.75	Spleen
ENSG00000185829.17	ARL17A	chr17:46528859:46570759:clu_12414:ENSG00000185829.17	46528859:46570759:clu_12414	2.0e-7	0.65	Brain - Cerebellum
ENSG00000214425.7	LRRC37A4P	chr17:45545676:45584572:clu_13175:ENSG00000214425.7	45545676:45584572:clu_13175	2.5e-7	0.54	Stomach
ENSG00000265547.1	RP11-293E1.2	chr17:45731944:45734284:clu_25866:ENSG00000265547.1	45731944:45734284:clu_25866	2.7e-7	-0.49	Testis
ENSG00000265964.1	RP11-293E1.1	chr17:45731944:45734284:clu_25866:ENSG00000265964.1	45731944:45734284:clu_25866	2.7e-7	-0.49	Testis
ENSG00000120088.14	CRHR1	chr17:45821440:45829215:clu_11872:ENSG00000120088.14	45821440:45829215:clu_11872	3.3e-7	-0.59	Brain - Cerebellar Hemisphere
ENSG00000225190.10	PLEKHM1	chr17:45458439:45475100:clu_13521:ENSG00000225190.10	45458439:45475100:clu_13521	3.8e-7	-0.32	Esophagus - Mucosa
ENSG00000225190.10	PLEKHM1	chr17:45458439:45475100:clu_13521:ENSG00000225190.10	45458439:45475100:clu_13521	3.8e-7	-0.32	Esophagus - Mucosa
ENSG00000159314.11	ARHGAP27	chr17:45401668:45402714:clu_25836:ENSG00000159314.11	45401668:45402714:clu_25836	4.6e-7	-0.51	Testis
ENSG00000185829.17	ARL17A	chr17:46528859:46570759:clu_14701:ENSG00000185829.17	46528859:46570759:clu_14701	5.0e-7	0.43	Skin - Not Sun Exposed (Suprapubic)
ENSG00000176681.14	LRRC37A	chr17:46331981:46332552:clu_25905:ENSG00000176681.14	46331981:46332552:clu_25905	5.6e-7	0.51	Testis
ENSG00000238083.7	LRRC37A2	chr17:46553449:46555152:clu_15053:ENSG00000238083.7	46553449:46555152:clu_15053	5.8e-7	-0.25	Skin - Sun Exposed (Lower leg)
ENSG00000185829.17	ARL17A	chr17:46528859:46570759:clu_13429:ENSG00000185829.17	46528859:46570759:clu_13429	6.2e-7	0.63	Prostate
ENSG00000159314.11	ARHGAP27	chr17:45398047:45402714:clu_14770:ENSG00000159314.11	45398047:45402714:clu_14770	6.6e-7	-0.45	Breast - Mammary Tissue
ENSG00000185829.17	ARL17A	chr17:46528859:46570759:clu_13973:ENSG00000185829.17	46528859:46570759:clu_13973	0.0000012	0.60	Pituitary
ENSG00000159314.11	ARHGAP27	chr17:45397074:45397949:clu_13509:ENSG00000159314.11	45397074:45397949:clu_13509	0.0000018	0.39	Esophagus - Mucosa
ENSG00000159314.11	ARHGAP27	chr17:45397074:45397949:clu_11669:ENSG00000159314.11	45397074:45397949:clu_11669	0.0000020	0.33	Whole Blood
ENSG00000186868.15	MAPT	chr17:45987095:45991460:clu_14798:ENSG00000186868.15	45987095:45991460:clu_14798	0.0000024	-0.34	Breast - Mammary Tissue
ENSG00000186868.15	MAPT	chr17:45990075:45991460:clu_11603:ENSG00000186868.15	45990075:45991460:clu_11603	0.0000026	0.43	Heart - Left Ventricle
ENSG00000214425.7	LRRC37A4P	chr17:45545676:45584572:clu_14241:ENSG00000214425.7	45545676:45584572:clu_14241	0.0000044	0.46	Colon - Transverse
ENSG00000233175.2	CTD-2020K17.3	chr17:45240604:45241111:clu_11351:ENSG00000233175.2	45240604:45241111:clu_11351	0.0000052	0.55	Brain - Caudate (basal ganglia)
ENSG00000204650.14	LINC02210	chr17:45630158:45636283:clu_12620:ENSG00000204650.14	45630158:45636283:clu_12620	0.0000055	-0.60	Spleen
ENSG00000204650.14	LINC02210	chr17:45630158:45636283:clu_12620:ENSG00000204650.14	45630158:45636283:clu_12620	0.0000055	-0.60	Spleen
ENSG00000073969.18	NSF	chr17:46704854:46710963:clu_13008:ENSG00000073969.18	46704854:46710963:clu_13008	0.0000065	0.27	Muscle - Skeletal
ENSG00000238083.7	LRRC37A2	chr17:46517433:46520212:clu_13522:ENSG00000238083.7	46517433:46520212:clu_13522	0.0000067	-0.41	Esophagus - Muscularis

p-value, eQTL association p value; NES, normalized effect size.

Table S23. MR results using BAG as exposure.

outcome	effect	se	pval	padj	Model
AD	-0.0034	0.0062	0.59	0.74	IVW
BIP	0.0467	0.0276	9.04x10-2	0.15	IVW
MDD	-0.0063	0.0212	0.77	0.77	IVW
PD	-0.4012	0.1413	**4.54x10-3**	**2.27x10-2**	IVW
SCZ	-0.0854	0.0372	**2.18x10-2**	5.46x10-2	IVW
AD	-0.0542	0.0172	**1.96x10-2**	**4.9x10-2**	Egger
BIP	0.0765	0.1162	0.53	0.53	Egger
MDD	0.1092	0.0874	0.26	0.32	Egger
PD	-1.4666	0.4074	**1.14x10-2**	**4.9x10-2**	Egger
SCZ	-0.3272	0.1254	**4.02x10-2**	6.7x10-2	Egger
AD	-0.0098	0.0061	0.11	0.14	wMed
BIP	0.0661	0.0272	**1.51x10-2**	**3.77x10-2**	wMed
MDD	-0.0207	0.02485	0.41	0.41	wMed
PD	-0.2741	0.0865	**1.53x10-3**	**7.65x10-3**	wMed
SCZ	-0.0537	0.0308	8.11x10-2	0.14	wMed
AD	-0.0034	0.0062	0.61	0.76	PRESSO
BIP	0.0467	0.0276	0.13	0.22	PRESSO
MDD	-0.0063	0.0212	0.77	0.77	PRESSO
PD	-0.2371	0.0861	7.06x10-2	0.22	PRESSO
SCZ	-0.073	0.0344	8.72x10-2	0.22	PRESSO
AD	0.0018	0.0011	0.11	0.197	RAPS
BIP	0.0127	0.0051	**1.27x10-2**	**4.87x10-2**	RAPS
MDD	0.004	0.0042	0.34	0.43	RAPS
PD	-0.0237	0.0101	**1.95x10-2**	**4.88x1-0-2**	RAPS
SCZ	0.0017	0.0051	0.74	0.738	RAPS
AD	2.14729	1	0.98	0.98	CAUSE
BIP	0.8014	1	0.79	0.98	CAUSE
MDD	1.9085	1	0.97	0.98	CAUSE
PD	-0.1551	1	0.44	0.98	CAUSE
SCZ	0.4667	1	0.68	0.98	CAUSE

The six MR models are: IVW, Inverse variance weighted model; Egger, Egger regression; wMed, weighted median; PRESSO, MR-PRESSO; RAPS, MR-RAPS and CAUSE. causal effect size padj, FDR corrected p value; AD, Alzheimer's disease; BIP, bipolar disorder; MDD, major depression disorder; PD, Parkinson's disease; SCZ, schizophrenia. Note, negative CAUSE estimates indicate the existence of causal relations.

Table S24. MR results using disorders as exposure.

exposure	effect	se	pval	padj	Model
AD	0.69	0.21	**7.9x10-4**	**3.95x10-3**	IVW
BIP	0.15	0.06	**1.35x10-2**	**3.37x10-2**	IVW
MDD	0.32	0.17	6.48x10-2	8.1x10-2	IVW
PD	-0.25	0.12	3.35x10-2	5.59x10-2	IVW
SCZ	0.06	0.04	0.14	0.14	IVW
AD	0.48	0.30	0.13	0.16	Egger
BIP	0.68	0.22	**3.67x10-3**	**9.18x10-3**	Egger
MDD	0.29	0.91	0.76	0.76	Egger
PD	-1.11	0.31	**1.96x10-3**	**9.18x10-3**	Egger
SCZ	0.39	0.14	**5.66x10-3**	**9.44x10-3**	Egger
AD	0.67	0.22	**2.40x10-3**	**1.2x10-2**	wMed
BIP	0.14	0.08	9.71x10-2	0.18	wMed
MDD	0.33	0.23	0.16	0.19	wMed
PD	-0.02	0.07	0.75	0.75	wMed
SCZ	0.08	0.05	0.11	0.18	wMed
AD	0.69	0.21	**2.22x10-3**	**1.11x10-2**	PRESSO
BIP	0.15	0.06	**1.67x10-2**	**4.11x10-2**	PRESSO
MDD	0.32	0.14	5.87x10-2	0.07.34x10-2	PRESSO
PD	-0.05	0.06	0.36	0.36	PRESSO
SCZ	0.08	0.04	**2.46x10-2**	**04.11x10-2**	PRESSO
AD	0.50	0.11	**6.97x10-6**	**3.485x10-5**	RAPS
BIP	0.05	0.02	**9.05x10-3**	**0.01.8x10-2**	RAPS
MDD	0.02	0.03	0.56	0.56	RAPS
PD	-0.01	0.01	0.451	0.556	RAPS
SCZ	0.04	0.02	**1.08x10-2**	**1.8x10-2**	RAPS
AD	-0.59	1	0.28	0.99	CAUSE
BIP	1.01	1	0.84	0.99	CAUSE
MDD	3.18	1	0.99	0.99	CAUSE
PD	1.44	1	0.93	0.99	CAUSE
SCZ	-0.09	1	0.47	0.99	CAUSE

The six MR models are: IVW, Inverse variance weighted model; Egger, Egger regression; wMed, weighted median; PRESSO, MR-PRESSO; RAPS, MR-RAPS and CAUSE. causal effect size padj, FDR corrected p value; AD, Alzheimer's disease; BIP, bipolar disorder; MDD, major depression disorder; PD, Parkinson's disease; SCZ, schizophrenia. Note, negative CAUSE estimates indicate the existence of causal relations.

References

1. Consortium, S.W.G.o.t.P.G. Biological insights from 108 schizophrenia-associated genetic loci. *Nature*, 421–427 (2014).
2. Ripke, S. Ricopili Pipeline And Standards of GWAS Analyses. *European Neuropsychopharmacology* **29**, S713-S714 (2019).
3. Lemieux Perreault, L.-P., Legault, M.-A., Asselin, G. & Dubé, M.-P. genipe: an automated genome-wide imputation pipeline with automatic reporting and statistical tools. *Bioinformatics* **32**, 3661-3663 (2016).
4. Rimol, L.M. *et al.* Cortical thickness and subcortical volumes in schizophrenia and bipolar disorder. *Biological psychiatry* **68**, 41-50 (2010).
5. Tønnesen, S. *et al.* White matter aberrations and age-related trajectories in patients with schizophrenia and bipolar disorder revealed by diffusion tensor imaging. *Scientific Reports* **8**, 14129 (2018).
6. Kelly, S. *et al.* Widespread white matter microstructural differences in schizophrenia across 4322 individuals: results from the ENIGMA Schizophrenia DTI Working Group. *Molecular Psychiatry* **23**, 1261-1269 (2018).
7. Watanabe, K., Taskesen, E., van Bochoven, A. & Posthuma, D. Functional mapping and annotation of genetic associations with FUMA. *Nat Commun* **8**, 1826 (2017).
8. Iotchkova, V. *et al.* GARFIELD classifies disease-relevant genomic features through integration of functional annotations with association signals. *Nature Genetics* **51**, 343-353 (2019).
9. Sul, J.H., Han, B., Ye, C., Choi, T. & Eskin, E. Effectively Identifying eQTLs from Multiple Tissues by Combining Mixed Model and Meta-analytic Approaches. *PLOS Genetics* **9**, e1003491 (2013).
